# Permittivity tensor imaging: modular label-free imaging of 3D dry mass and 3D orientation at high resolution

**DOI:** 10.1038/s41592-024-02291-w

**Published:** 2024-06-18

**Authors:** Li-Hao Yeh, Ivan E. Ivanov, Talon Chandler, Janie R. Byrum, Bryant B. Chhun, Syuan-Ming Guo, Cameron Foltz, Ezzat Hashemi, Juan A. Perez-Bermejo, Huijun Wang, Yanhao Yu, Peter G. Kazansky, Bruce R. Conklin, May H. Han, Shalin B. Mehta

**Affiliations:** 1 Chan Zuckerberg Biohub, San Francisco, CA USA; 2 Stanford University, Palo Alto, CA USA; 3 Gladstone Institutes, San Francisco, CA USA; 4 University of Southampton, Southampton, UK; 5 University of California San Francisco, San Francisco, CA USA; 6 Present Address: ASML, San Jose, CA USA; 7 Present Address: California’s Stem Cell Agency, South San Francisco, CA USA; 8 Present Address: Eikon Therapeutics, Hayward, CA USA; 9 Present Address: Insitro, South San Francisco, CA USA; 10 Present Address: Quantinuum, Broomfield, CO USA; 11 Present Address: Genentech, South San Francisco, CA USA

**Keywords:** Optical imaging, Polarization microscopy, Software, Phase-contrast microscopy

## Abstract

The dry mass and the orientation of biomolecules can be imaged without a label by measuring their permittivity tensor (PT), which describes how biomolecules affect the phase and polarization of light. Three-dimensional (3D) imaging of PT has been challenging. We present a label-free computational microscopy technique, PT imaging (PTI), for the 3D measurement of PT. PTI encodes the invisible PT into images using oblique illumination, polarization-sensitive detection and volumetric sampling. PT is decoded from the data with a vectorial imaging model and a multi-channel inverse algorithm, assuming uniaxial symmetry in each voxel. We demonstrate high-resolution imaging of PT of isotropic beads, anisotropic glass targets, mouse brain tissue, infected cells and histology slides. PTI outperforms previous label-free imaging techniques such as vector tomography, ptychography and light-field imaging in resolving the 3D orientation and symmetry of organelles, cells and tissue. We provide open-source software and modular hardware to enable the adoption of the method.

## Abstract (web summary)

Permittivity tensor imaging is a label-free computational microscopy approach that enables the three-dimensional measurement of molecular permittivity tensors, revealing information about a biomolecule’s dry mass and orientation in cells and tissues.

## Main

Label-free imaging of biomolecules with electrons, light and radio waves has enabled multiple biological discoveries. Electron microscopy (EM) reports the charge distribution in fixed specimens and provides structural insights with a spatial resolution of around 1 nm but is currently limited to about 1 mm^3^-sized tissues^1^ despite time and labor-intensive effort. Magnetic resonance imaging (MRI) reports the distribution of hydrogen density and can image the dynamic architecture of organs deep into the body but is currently limited to a resolution of 100 μm (ref. ^2^). Label-free light microscopy can report the density and anisotropy of biomolecules with a spatial resolution of 250 nm and bridges the resolution gap between EM and MRI. Label-free imaging across spatial scales of 250 nm to 1 cm reveals the architecture of organelles, cells and tissues. Measurement of these material properties complements the measurement of molecular distribution with fluorescent or histology labels. Quantitative label-free imaging can enable new investigations in biology and pathology, for example, the discovery of cellular impacts of infections, mechanical properties of cytoskeleton and extracellular matrix, image-based fingerprinting of cell types and cell states, measurement of myelination and mesoscale connectivity in brain tissue and rapid diagnosis of pathology in histological sections.

Biomolecules (nucleic acids, proteins, lipids and carbohydrates) form ordered assemblies that underpin the anisotropic functions of organelles, cells and tissues. Their architectural order is described by tensor quantities such as diffusion, elasticity and permittivity. Biomolecules are dielectric at visible wavelengths (the electrons bound to biomolecules are displaced in response to an applied electric field), but they do not conduct electric current. The relative permittivity of a dielectric material quantifies how much the bound electrons are polarized by an applied electric field. The polarization of material refers to the displacement of bound electrons in an external field, whereas the polarization of light is the orientation of the electric field of the light wave. The more easily a material is polarized, the more it delays the phase of an electromagnetic wave traveling through it and the higher the permittivity of the material. If the bound electrons resonate with the incident optical frequency, the material absorbs the light. At visible wavelengths, cells and tissues mostly alter the phase of light but do not absorb it, which makes them transparent. The permittivity of an anisotropic structure depends on the direction and the polarization of incident light^3^. The anisotropic architecture is described succinctly by the PT^4^, a 3 × 3 matrix that describes the permittivity of a material at every point in space.

The PT consists of isotropic and anisotropic components that report the dry mass and orientation of the biomolecules. The isotropic PT and anisotropic PT are intrinsic properties of the biological material. Microscopes record properties of light, such as intensity, phase and polarization state. The isotropic component of PT is encoded by the polarization-independent absorption and phase delay of light, whereas the anisotropic component of PT is encoded by the polarization-dependent absorption (diattenuation) and phase delay (retardance) of light. In conventional microscopy, the information diversity in the acquired data and the image information models are often inadequate to untangle the intrinsic material properties from the properties of light. We jointly designed an acquisition scheme that encodes the invisible PT into the image data and an inverse algorithm that decodes the PT from image data using an accurate imaging model. More precisely, our method reveals the relative PT. The refractive index (RI) of biomolecules is the square root of their isotropic permittivity.

Many quantitative label-free light microscopy methods image either the isotropic component or the anisotropic component of PT projected on the focus plane. Quantitative phase-microscopy images the isotropic component of PT in terms of the distribution of RI^5,6^, which is proportional to the density of biomolecules with the scaling factor of specific refractive index increment^7^. Optical diffraction tomography^8,9^ and shearing interferometry methods^10^ that account for diffraction effects also measure distribution of RI. Quantitative polarization microscopy, on the other hand, encodes the anisotropic component of PT in terms of the retardance of light and has been used to study microtubule spindles^11,12^, white matter in human brain tissue slices^13–16^ and collagen architecture in eye tissues^17^. Phase and polarization imaging is also used to quantify the optical properties of fabricated materials^18,19^. Although the isotropic and anisotropic components of the PT are induced simultaneously when light interacts with the matter, they are commonly not measured simultaneously.

The anisotropic angular distribution of biomolecular assemblies can give rise to distinct permittivity (or RI) along the three principal axes of the material’s symmetry at each point in space; however, many biological structures, such as axon bundles, collagen fibers, filaments of cytoskeletal and motor proteins, plasma membrane, nuclear envelope and mitochondria, have a single symmetry axis that results in two distinct RIs, ordinary index perpendicular to the axis of symmetry and extraordinary index parallel to the axis of symmetry. Such structures can be described by a uniaxial PT, which is a second-order tensor with two of the three eigenvalues being equal. Throughout this paper, we assume that the biological material has uniaxial symmetry, which is a correct assumption for a large range of structures with isotropic, linear or planar symmetries. When the PT is biaxial, for example, when collagen fibers cross the same resolved volume at diverse orientations, our method measures the uniaxial component.

Imaging the biological structures in terms of its PT is an active area of research. As we are discussing the distribution of biomolecules in both spatial and angular dimensions, we use the following terminology to clarify the spatial and angular dimensions: two-dimensional (2D) plane and 3D volume imply spatial dimensions, 3D anisotropy implies angular distribution of biomolecules and 2D anisotropy is the angular projection of 3D anisotropy on the image plane. A complete description of PT consists of mean permittivity (reports the isotropic dry mass), differential permittivity (reports the anisotropic dry mass), 3D orientation and material symmetry (optic sign) throughout 3D space. These physical properties are different channels of information measured in 3D space.

Currently reported methods measure PT with various degrees of completeness. Several methods employ geometric imaging models and do not achieve diffraction-limited resolution. Shribak et al. reported a method combining orientation-independent differential interference contrast microscopy (OI-DIC) and orientation-independent polarization microscopy that reports density and 2D anisotropy in a 2D plane, without considering diffraction effects^20^. Our recent work, quantitative label-free imaging with phase and polarization (QLIPP), combines phase from defocus^21–23^ with quantitative polarization microscopy^13,24–26^ to phase and retardance^27^ in a 3D volume. Saba et al.^28^ reported a polarization-sensitive coherent optical diffraction tomography analogous to QLIPP. Vector ptychography methods that account for diffraction effects enable 2D imaging^29,30^ and 3D imaging^31^ of dry mass and 2D anisotropy. QLIPP^27^, vector ptychography^29,31^ and polarization-sensitive ODT^28^ report the 2D anisotropy projected on the imaging plane.

We report PTI, a computational microscopy method for diffraction-limited measurements of uniaxial PT, consisting of mean permittivity, differential permittivity, 3D orientation and optic sign (symmetry), in 3D volume and 2D planes. PTI captures these properties of the specimen by combining oblique illumination^32–34^ with polarization-sensitive detection^13,25,35–39^. We implement this design as an inexpensive add-on module on a standard wide-field microscope. We develop a vectorial imaging model and the corresponding multi-channel inverse algorithm to extract the spatial distribution of the components of the uniaxial PT. Our work advances the field of computational label-free imaging as follows: (1) PTI enables volumetric measurements of dry mass and 3D orientation of biological materials at diffraction-limited resolution for the first time. This enables imaging of biological architecture that has been challenging to image with earlier methods. After we preprinted our work on PTI^40^, Shin et al.^41^ reported a holographic approach to measure PT to analyze the material properties of liquid crystals. (2) We report direct measurements of symmetry (optic sign) of biological specimens. (3) Our vector diffraction model and inverse algorithm balance the tradeoff between accuracy and computational complexity. They can be adapted to improve the accuracy and resolution of emerging non-holographic label-free vector imaging systems.

We validate our vectorial imaging model with rigorous electromagnetic simulations using the finite-difference time-domain (FDTD) algorithm. We illustrate the key measurements and test the accuracy of the inverse algorithm with simulated specimens of various optical properties. We validate the accuracy and resolution with isotropic polystyrene beads and anisotropic laser-written glass targets. We demonstrate that PTI allows analysis of the architecture of the mouse brain at scales of the whole slice, axon bundles and single axons. We show that PTI measurements can be multiplexed with fluorescence deconvolution microscopy to image the physical and molecular architecture of the organelles in SARS-CoV-2-infected induced pluripotent stem (iPS) cell-derived cardiomyocytes (CMs) and respiratory syncytial virus (RSV)-infected A549 cells. Finally, we show that PTI can be multiplexed with hematoxylin and eosin (H&E) imaging for histological analysis. These data establish a new label-free measurement technology for comprehensive high-resolution imaging of biological architecture. With our modular and inexpensive optical design and open-source software, we aim to enable rapid adoption and refinement.

## Results

### Computational imaging concept

#### Light path

**Fig. 1:**
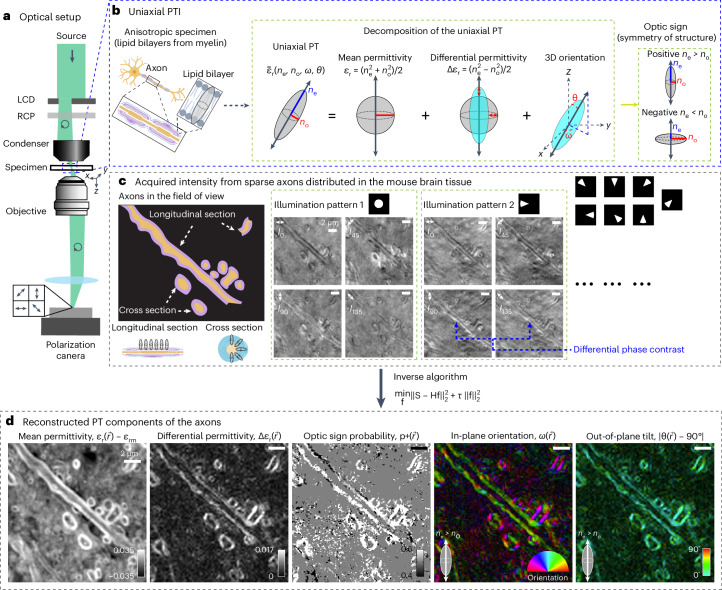
Concept and process of measuring the uniaxial PT. **a**, Light path of the microscope, including an LCD for generating oblique illumination, an RCP and a four-channel polarization-sensitive camera. **b**, Illustration of the components of the PT mean permittivity (isotropic component), differential permittivity (anisotropic component), 3D orientation and optic sign. The expected permittivity distribution of an ordered lipid bilayer in the myelinated axons is illustrated. Like most biomolecular assemblies, the angular distribution of PT is uniaxially symmetric, represented by an ellipsoid. The optic sign indicates a prolate (positive sign) or an oblate (negative sign) distribution around the axis of symmetry. **c**, An example field of view with longitudinal sections and cross-sections of myelinated axons illustrates how image contrast varies with the PT of the specimen, the polarization orientation of the detector and the illumination angle. The illumination angle is controlled by transparent sectors of the LCD shown as bright disks and sectors. **d**, Using an inverse algorithm based on convex optimization, we reconstruct 3D mean permittivity, 3D differential permittivity, 3D optic sign probability and 3D orientation of the axons in the example field of view from intensities. We report the 3D orientation of the symmetry axis of the PT, independent of its optic sign. The 3D orientation consists of two components rendered in two false-color images: the in-plane orientation (*ω*) and out-of-plane tilt (∣*θ* − 90^∘^∣) are shown by the color wheel and the color bar, respectively. The brightness of the color reports the differential permittivity.

**Extended Data Fig. 1:**
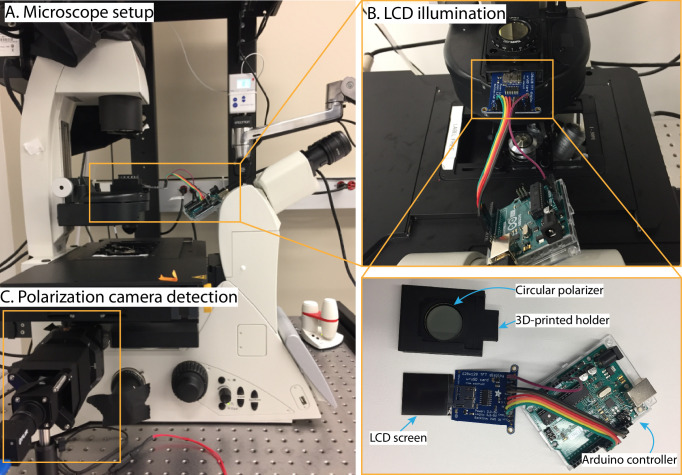
Photographs of the components of the PTI system. **(A)** The microscope setup. **(B)** The oblique illumination module composed of adafruit LCD screen (Adafruit, ST7735R) and the circular polarizer (Thorlabs, CP1R532). **(C)** The polarization camera (FLIR, BFS-U3-51S5P-C) for detection.

Figure 1a and Extended Data Fig. 1 show the optical layout and the components needed to implement PTI on a standard research microscope. We implemented PTI on a Leica DMi8 inverted microscope with two add-on modules, an oblique illuminator and a polarization imaging module. The oblique illuminator is composed of a green color filter, a linear polarizer, a programmable amplitude modulator, a right-hand circular polarizer (RCP) and a condenser lens. The light from an LED source is first filtered by a green filter and the linear polarizer before passing through the amplitude modulator placed in the front focal plane of the condenser lens. The amplitude modulator is constructed from a low-cost liquid crystal display (LCD; Adafruit, ST7735R) with its backlight removed. The RCP (Thorlabs, CP1R532) is placed after the amplitude modulator. This module enables computer-controlled oblique illumination with circularly polarized light. It is compact enough to be placed at the front focal plane of a high-numerical aperture (NA) condenser. The oblique illuminator can illuminate the specimen with an NA as high as 1.4 with high light-coupling efficiency. The oblique circularly polarized light interacts with the specimen and is collected by the polarization imaging module. The polarization imaging module consists of the microscope objective, tube lens and a four-channel polarization-sensitive camera (FLIR, BFS-U3-51S5P-C). The polarization camera has a patterned grid of linear polarizers on top of pixels, with transmission axes along 0°, 45°, 90° and 135°. The camera images four linearly polarized light states with a single exposure. Using on-axis illumination, a microscope equipped with this camera can capture the projected retardance and 2D orientation of material’s slow axis similar to other polarized light microscopes^13,26,27,35,38,39^. The high-quality oblique illuminator enables the acquisition of 3D orientation. Our modular design enables tomographic polarization imaging of specimens with diverse oblique illumination patterns. As shown in Supplementary Video 1, the components of the PTI module can be readily added to an existing microscope.

#### Interpretation of permittivity tensor

The PT of a complex specimen can be decomposed into the isotropic component and the anisotropic component at each point in space. To build intuition by analogy, we compare the PT with diffusion tensor (DT) that is commonly measured with DT imaging (DTI). The PT is mathematically analogous to, but physically distinct from the DT. PT reports the architectural symmetries of biomolecules in cells and tissues, just as DT reports the symmetries of diffusion pathways. DTI is widely used to map the spatio-angular architecture of white matter in the brain. The isotropic component of PT is similar to the mean diffusivity component of DT, the anisotropy of PT is similar to the fractional anisotropy component of DT and the 3D orientation of anisotropy of PT is similar to the 3D orientation of the axial diffusivity of DT^42^. Both PTs and DTs are measured with finite spatial resolution (the measured tensor properties are integrated over the spatial resolution of the instrument). We report PTI at the diffraction-limited 3D spatial resolution of 0.25 × 0.25 × 0.8 = 0.05 μm^3^. DTI is suitable for organ-to-tissue-level imaging deep inside the specimen’s body, whereas PTI is suitable for organelle, cell and tissue-level imaging up to 100 μm deep.

Figure 1b illustrates how 3D PT of a lipid bilayer can be described by an ellipsoidal surface parameterized by: 3D orientation (in-plane orientation, *ω* and inclination, *θ*) of the symmetry axis, the ordinary RI (*n*_o_) experienced by the electric field polarized perpendicular to the symmetry axis and the extraordinary RI (*n*_e_) experienced by the electric field polarized parallel to the symmetry axis. The spatial and angular resolution of spatio-angular imaging methods are fundamentally limited by the diffraction of light. Therefore, any far-field imaging method, including PTI measures a blurred PT of the material.

Among multiple equivalent decompositions of uniaxial PT (Supplementary Note 1), we chose to reconstruct a decomposition that facilitates the interpretation of biological architecture: (1) the mean permittivity, which reports the dry mass of biomolecules and is related to phase measurements; (2) the differential permittivity, which reports anisotropy of biomolecules and is related to polarization measurements; (3) the 3D orientation of the symmetry axis; and (4) the optic sign, which reports the type of symmetry of PT.

**Fig. 2:**
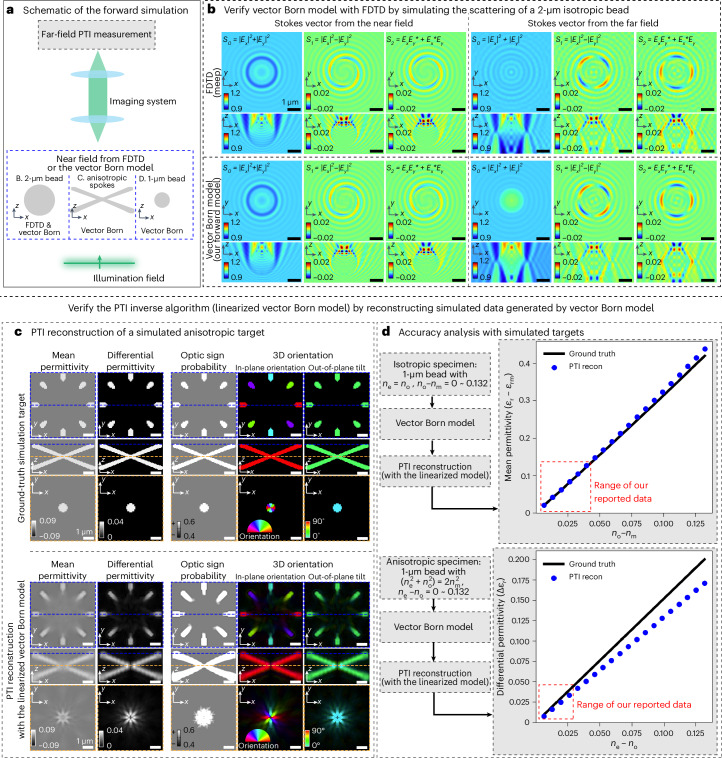
Verification of PTI with simulations. We benchmarked the accuracy of the imaging model and the inverse algorithm by comparing reconstructed properties with the simulated ground truth. **a**, Schematic of the forward simulations reported in **b**–**d** illustrates locations and shapes of the simulated targets, imaging models used for simulation and the distinction between near-field and far-field measurements. **b**, Comparison of Stokes vectors simulated by our vector Born model with the FDTD simulator using 2-μm isotropic bead illuminated with RCP light. Simulations of the polarization state of scattered light are shown for both near-field and in far-field. RI of the bead is *n*_bead_ = 1.59 and of surrounding medium is *n*_m_ = 1.58. The agreement of the Stokes vectors from both models indicates that our imaging model’s accuracy is comparable to the gold-standard FDTD simulator in weak-scattering specimens. Vector Born and FDTD simulations show that the light scattered by isotropic edge acquires polarization-dependent contrast in *S*_1_ and *S*_2_ channels, similar to an anisotropic edge. This phenomenon is called edge birefringence. **c**, Ground truth and PTI reconstructed 3D mean permittivity, 3D differential permittivity, optic sign probability and 3D orientation of a simulated target. The 3D orientation is rendered in two separate images: in-plane orientation and out-of-plane tilt. **d**, Analysis of the accuracy of the 3D mean permittivity and 3D differential permittivity reconstructed from simulated PTI measurements of the targets of varying average RI and varying birefringence. The boxes outlined with the dashed red lines mark the range of the physical quantities reported in our experimental data, where PTI reports accurate measurements.

**Fig. 3:**
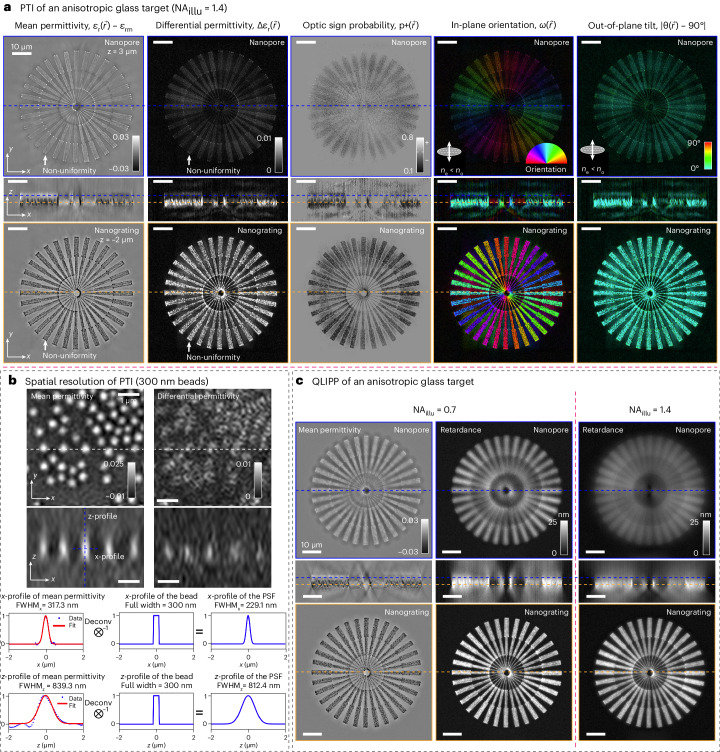
3D spatial resolution of PTI. **a**, *x*–*y* and *x*–*z* sections of 3D mean permittivity, 3D differential permittivity, optic sign probability and 3D orientation (in-plane orientation and out-of-plane tilt) volumes of a laser-written anisotropic glass target enable identification of nanograting and nanopore structures at different depths. The nanopore layer is shown at the top and indicated by blue dashed line in *x*–*z* sections. The nanograting layer is shown at the bottom and indicated by orange dashed line in *x*–*z* sections. The elliptical legend in the 3D orientation maps indicates that the orientation is reconstructed assuming negative uniaxial material. **b**, Characterization of 3D spatial resolution by imaging 300-nm polystyrene beads with RI of 1.5956 immersed in oil with RI of 1.5536. The mean permittivity images of beads show dense center and differential permittivity images resolve edges of the beads. The mean permittivity of the center bead is selected for Gaussian fits in *x* and *z* directions. The Gaussian fits are deconvolved with the physical size of the bead to measure the FWHM of the PSF in *x* and *z*. **c**, *x*–*y* and *x*–*z* sections of the 3D phase and retardance of the same target measured using QLIPP with two different illumination NAs (0.7 and 1.4) show spatial resolution and contrast lower than in PTI measurements. The 3D orientation and optic sign are not accessible with QLIPP. Source data

**Fig. 4:**
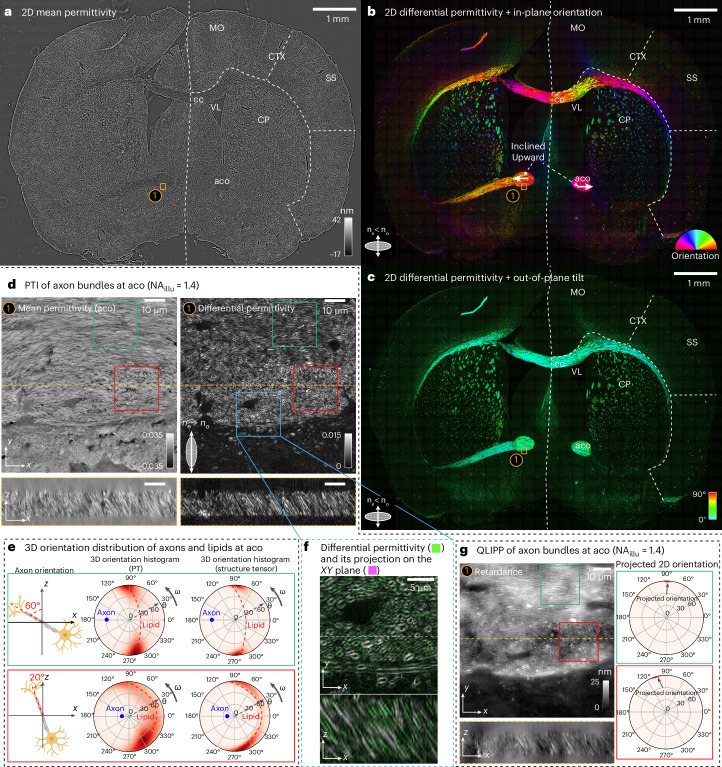
Multi-scale imaging of the architecture of an adult mouse brain section with PTI. **a**, 2D mean permittivity. **b**, 2D differential permittivity and in-plane orientation. **c**, Out-of-plane orientation images of the whole brain section show key anatomical landmarks. At the imaging and illumination NA of 0.55, axons are negative uniaxial structures with symmetry axes along the length of axons. We label anatomical landmarks using the coronal section at level 51 of the Allen brain reference atlas. MO, motor cortex; SS, somatosensory area. **d**, aco area marked with orange boxes (labeled with (1) in **a**–**c**) is imaged in 3D with imaging NA of 1.47 and illumination NA of 1.4. The orthogonal sections of 3D mean and differential permittivity show complex axon networks. We resolve the boundaries of individual axons, which are positive uniaxial structures with symmetry axes perpendicular to the membrane. **e**, We assess the 3D orientation distribution of the measured PT and the structure tensor of two volumes identified with green and red boxes (see text). The blue dot in each histogram indicates the corresponding axon orientation in the selected volume and the red dashed lines indicate the orientations perpendicular to the axon. **f**, The overlay of the angularly projected differential permittivity (magenta) and the differential permittivity (green) from the blue box in **d** shows that differential permittivity shows more axons. **g**, Orthogonal sections of the retardance of the same volume at aco measured using QLIPP with the same NA show spatial resolution and contrast worse than PTI measurements. Source data

Materials are considered to have a positive optic sign or negative optic sign depending on their symmetry^4^ as illustrated in Fig. 1b and simulated in Fig. 2. For example, the lipid bilayer is a positive uniaxial material, whereas the anisotropic glass targets reported later (Fig. 3) and axons (Fig. 4) are negative uniaxial materials. The 3D orientation reports the symmetry axis of the material in each voxel. 3D orientation aligns with the slow axis of the material (axis with higher RI), when the material is positive uniaxial. The 3D orientation aligns with the fast axis of the material (axis with lower RI), when the material is negative uniaxial. The polarized light imaging methods reported earlier do not measure the optic sign of the material and only report the orientation of the symmetry axis.

#### Encoding PT in images

**Extended Data Fig. 2:**
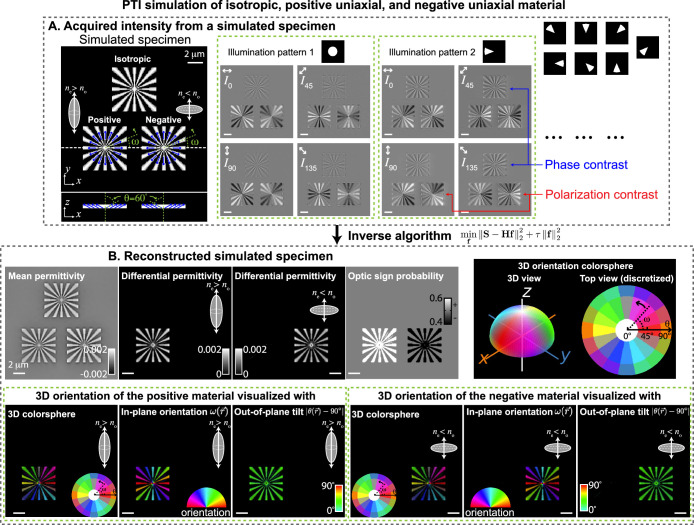
PTI simulation and reconstruction of isotropic, positive uniaxial, and negative uniaxial test targets. **(A)** Simulated raw images of a structure consisting of isotropic material (*n*_e_ = *n*_o_ = 1.52), positive uniaxial material (*n*_e_ = 1.521, *n*_o_ = 1.519), and negative uniaxial material (*n*_e_ = 1.519, *n*_o_ = 1.521) oriented radially (along the spokes) and inclined with *θ* = 60^∘^ illustrate dependence of intensity contrast on the polarization and the illumination pattern. The RI of the surrounding medium is set to 1.518. Each target is 5.7 × 5.7 μm^2^ in size. We set the NA of illumination to 1.4 and the NA of the objective to be 1.47 to mimic the experimental conditions. Simulated images capture specimen properties. Variations in mean permittivity are visible with off-axis illumination (illumination pattern 2), while variations in differential permittivity are better observed across polarization channels. We also observe contrast variations across on-axis and off-axis illumination caused by the out-of-plane orientation and optic sign. **(B)** Using an inverse algorithm based on convex optimization, we reconstruct 3D mean permittivity, 3D differential permittivity, optic sign probability, and 3D orientation of the simulated target from intensities. The 3D orientation is rendered with two color schemes: The first scheme (‘3D color sphere‘) is to render 3D orientation by a single false-color image in which the 3D orientations is shown by the color and the differential permittivity is shown by the brightness of the color. The second scheme is to separate 3D orientation into in-plane orientation and out-of-plane tilt as shown in Fig. 1d). For color sphere visualization, we adopted spherical colormap from^78^ (for example *ω* = 0^∘^ and *θ* = 45^∘^ for red, *ω* = 180^∘^ and *θ* = 45^∘^ for yellow). The 3D view and the projection of the top view of the color sphere are shown in B for ease of reference.

We encode invisible PT into visible intensities using diverse illumination angles and polarization states of light. Experimental data in Fig. 1 and wave optical simulations of images of a target in Extended Data Fig. 2 illustrate how components of PT are encoded by the variations in the image contrast.

Figure 1c shows raw images from an example field of view containing longitudinal sections and cross-sections of axons in the mouse brain tissue section. The sample is illuminated with 1.4 NA and imaged with an objective of 1.47 NA. Under both the circular (illumination pattern 1) and the sector (illumination pattern 2) illumination patterns on the LCD, we see strong intensity modulations across polarization channels due to the anisotropy of myelin sheath consisting of multiple lipid bilayers. When the illumination pattern is a sector (illumination pattern 2), edges of the middle longitudinal axon (indicated by blue arrows) show an intensity gradient perpendicular to the axon in addition to the polarization intensity modulation, demonstrating the multiplexing of differential phase contrast^32–34^ and the polarization contrast.

The simulated target in Extended Data Fig. 2 consists of an isotropic spoke pattern and two anisotropic spoke patterns with defined 3D orientation, uniaxial symmetry and opposite optic signs. The intensity modulations induced by the variations in the phase of the isotropic material and the anisotropic materials is visible under a sector illumination. We also observe that the anisotropic spoke patterns cause differential intensity modulations across the four polarization channels when the on-axis (brightfield) and the off-axis (sector) illuminations are used, which are caused by the difference in the optic sign and the 3D orientation of the symmetry axis^36^.

**Extended Data Fig. 3:**
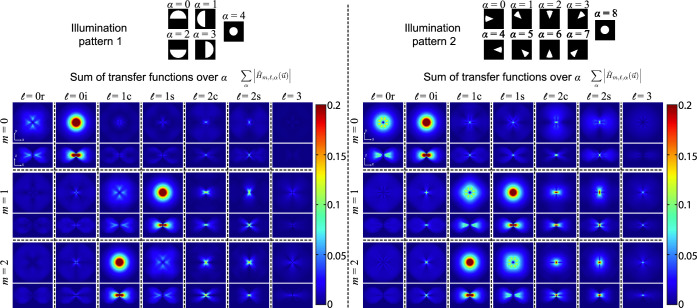
Comparison of transfer functions for two possible sets of illumination patterns. We show transverse (*x* − *y*) and axial (*x* − *z*) cross-sections of the transfer functions that map the *ℓ*-th individual scattering potential tensor component to the *m*-th Stokes parameter. We show the magnitudes of the transfer functions summed over all illumination patterns (*α*) for two sets of illumination patterns: (left) semi-circular patterns typically used for differential phase contrast, combined with lower-NA brightfield illumination, (right) the sector illumination combined with lower-NA brightfield illumination. We adopt the right set of illumination pattern, which more uniformly transfers 3D spatial frequencies of all tensor components.

For a 2D specimen thinner than the depth of field of the microscope, we acquire 36 2D images (nine oblique illuminations with four polarization channels) for data reconstruction. If the specimen is 3D (the thickness of the specimen is larger than the depth of field of the microscope), we collect 36 3D *z*-stacks (nine oblique illuminations with four polarization channels at each plane) with a *z*-step of half of the depth of field (typically 250–300 nm in our 3D experiments). To account for background polarization effects introduced by components in the optical path other than the specimen, we also collect a dataset (36 2D images from nine oblique illuminations with four polarization channels) at an empty field of view, which is used in the reconstruction of the physical properties of the specimen. The choice of illumination patterns projected on the LCD is discussed in Supplementary Note 5 and Extended Data Fig. 3.

Accurate reconstruction of components of PT from measured intensities at diffraction-limited resolution requires a vectorial partially coherent imaging model that expresses intensities in terms of the components of PT. We report a vector Born model that expresses the specimen’s relative permittivity as the scattering potential tensor and vector properties of light in the image plane as the Stokes vector. The model is widely useful for partially coherent vector imaging systems. The model is summarized in Methods (‘Imaging model’) and derived in Supplementary Note 1. Supplementary Note 2 describes the measurement of the Stokes vector in the imaging volume. The Stokes vector, which is defined in equation (5), describes the polarization state of the scattered light with *S*_0_ describing the total intensity, *S*_1_ and *S*_2_ describing how much the electric field is linearly polarized.

Scalar scattering potential is a key concept employed in the diffraction tomography of 3D RI (density of bound electrons)^43^. The scalar scattering potential has been extended to a 2 × 2 scattering potential tensor^28,44^ to enable volumetric reconstruction of 2D anisotropy (anisotropy projected on the imaging plane). Our work generalizes the concept to measure a more complete 3 × 3 scattering potential tensor, which allows reconstruction of volumetric distribution of density, 3D anisotropy and material symmetry with diffraction-limited resolution. The key assumption underlying vector Born model is that the specimen scatters light weakly^4^ such that measured intensities are dominated by the light that is scattered only once. This assumption is widely referred to as first Born approximation. The relationship between the Stokes vector and the scattering potential tensor is nonlinear even after the first Born approximation. Reconstruction of uniaxial PT with vector Born model requires a computationally expensive iterative algorithm. We make a further approximation of weak object to arrive at linearized vector Born model that leads us to an efficient inverse algorithm for reconstruction of scattering potential tensor. The linearized vector Born model consists of a set of optical transfer functions (OTFs) that relate 3D Fourier transforms of the Stokes volumes with the 3D Fourier transforms of the scattering potential tensor components. We evaluate the regime of validity of first Born approximation and weak object approximations in the context of PTI through simulations (Fig. 2) and experiments.

The above assumptions are typically valid for cells and tissues ~50–200-μm thick depending on the scattering properties of the specimen and the wavelength of light. This imaging model enables the development of an inverse algorithm that retrieves the uniaxial PT from high-dimensional acquisition.

#### Reconstruction of PT

Before reconstructing PT, we calibrate the instrument matrix that relates Stokes parameters of light and the measured intensities, convert the measured intensities into the Stokes parameters and perform a background correction (Supplementary Note 2). The inverse algorithm (Supplementary Note 3) converts the Stokes parameters into optical properties shown in in Fig. 1b. This computational framework allows us to transform the input intensities from Fig. 1c into diffraction-limited measurements of mean permittivity, differential permittivity, optic sign probability and 3D orientation of the specimen as shown in Fig. 1d.

Specifically, we reconstruct:

1. Mean permittivity, which is relative permittivity (*ϵ*_r_ − *ϵ*_rm_) integrated over resolved volume.
2. Differential permittivity, which is the difference between ordinary and extraordinary permittivity (Δ*ϵ*_*r*_) integrated over a resolved volume. The ordinary and extraordinary RI are square roots of respective permittivities. As the measurement of differential permittivity is noisier than mean permittivity, we developed various denoising methods for PT as described in Supplementary Note 4.2.
3. 3D orientation of the symmetry axis (in-plane orientation, *ω* and inclination, *θ*). The 3D orientation is rendered with two images, the in-plane orientation, *ω* and the out-of-plane tilt, ∣*θ* − 90°∣, which are introduced in Fig. 1d. Note that the inclination, *θ*, is the polar angle in the spherical coordinates, relative to the *z* axis, whereas the out-of-plane tilt, ∣*θ* − 90°∣, is the absolute tilt angle relative to the *x*–*y* plane.
4. Optic sign, which reports the type of symmetry around the symmetry axis (*p*_+_ = *n*_e_ ≷ *n*_o_).

When the specimen is thicker than the z-resolution of the PTI setup, we acquire 3D data. The 3D mean and differential permittivity are reported in identical units of relative permittivity, enabling quantitative comparison of the isotropic and anisotropic dry mass of the material across the volume. When the specimen is thinner than the z-resolution of the PTI setup (for example Fig. 4a–c), the permittivity is integrated along the depth of field, resulting in a 2D permittivity.

The Python software that implements the imaging model, the inverse algorithm, the simulations and the reconstruction of experimental data are available at https://github.com/mehta-lab/waveorder.

### Verification of the imaging model and the inverse algorithm

Before reporting experimental PTI measurements, we verify the accuracy of the vector Born model (equation (5)), linearized vector Born model (equation (6)) and our multi-channel inverse algorithm (Supplementary Note 3). Figure 2a summarizes the geometry, type of specimens and the models used for the simulations. The vector Born model is first verified with the rigorous and computationally expensive solver of Maxwell’s equation. Once verified, we use the vector Born model for accurate and fast simulations of PTI measurements. To verify the inverse algorithm of PTI, based on the more approximate linearized vector Born model, we reconstruct PT from simulated data and compare the reconstructed physical properties with the ground truth optical properties that were inputs for simulations.

#### Verification of the vector Born model

The vector Born model accurately describes vectorial light-matter interaction under the assumption of single scattering. As this model is employed for computational microscopy for the first time, we verified its accuracy by comparing the scattered vector fields simulated using this model and simulated using a rigorous solver for Maxwell’s equation, FDTD algorithm (meep)^45^. We simulated an RCP wave propagating along the *z* axis. To keep the simulation computationally efficient, we chose a 2-μm isotropic bead (refractive indices of the bead and the surrounding medium are *n*_bead_ = 1.59 and *n*_m_ = 1.58) as the simulated target. Figure 2b shows the near-field and far-field Stokes vectors of the scattered vector fields using the FDTD algorithm (top) and the vector Born model (bottom). The comparison of near-field and far-field results allows us to evaluate which information about the specimen properties is lost due to the propagation of light and therefore inaccessible to far-field microscopes, such as PTI.

Even though the vector Born model only accounts for single-scattered photons, it recapitulates the interference patterns in the near-field Stokes vectors seen with the FDTD simulation. In the far field, the vector Born model captures most of the features observed in the FDTD simulation, except that the *S*_0_ component differs between the two simulations. We see fewer interference fringes in the *S*_0_ predicted by the vector Born model than in the *S*_0_ predicted by the FDTD. This difference is because the vector Born model does not account for the multiple scattered photons.

**Extended Data Fig. 4:**
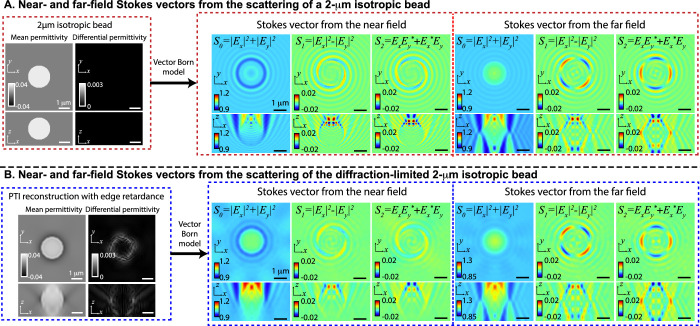
The ambiguity in the far-field measurements caused by the diffraction limit. We illustrate the fundamental ambiguity in the measurement of mean and differential permittivity caused by the diffraction limit by simulating the electric field scattered by two objects: **(A)** a 2-μm isotropic sphere refractive indices of the bead and the surrounding media are *n*_bead_ = 1.59 and *n*_m_ = 1.58) and **(B)** an object with edge birefringence, which is actually the PTI reconstruction of the isotropic bead. We used our vector Born model to simulate the electric field in the near- and far-field. We assumed that the objects were illuminated with right-hand circular polarization. We report the scattered fields in terms of the Stokes parameters. The far-field Stokes vectors in (A) and (B) are quite similar, whereas the near-field Stokes vectors in (A) show finer interference patterns and edges of the bead than the corresponding results in (B). This clarifies that the far-field (diffraction-limited) measurements cannot distinguish an isotropic edge and a diffraction-limited anisotropic edge.

Even though the specimen is isotropic, far-field *S*_1_ and *S*_2_ components simulated by FDTD and vector Born simulations show orientation-dependent modulations around the boundary of the bead. This weak modulation suggests that an isotropic bead can change the polarization state of the incident light similar to an anisotropic material. This phenomenon is called the edge birefringence and is caused by the polarization-dependent Fresnel reflection at the interface of two different isotropic materials^46^. Due to the diffraction-limited resolution of the far-field measurement, there is an inherent ambiguity in distinguishing a weak birefringence due to the shape of molecular assembly (form birefringence) from weak birefringence due to an isotropic edge (edge birefringence). This is a source of ambiguity in angular measurements. PTI reconstruction estimates that the specimen has a weak differential permittivity at the edge as seen in Extended Data Fig. 4b. To evaluate whether this ambiguity can be resolved, we conducted a forward simulation using the permittivity of the reconstructed bead (a blurry isotropic bead with an anisotropic edge). The far-field Stokes vector of this forward simulation was similar to the one generated with the isotropic bead, whereas the near-field Stokes vector was very different. These data illustrate that these two types of specimens can be distinguished from the information contained in the near-field region, but not from the information contained in the far-field region. In other words, this ambiguity in the reconstruction of the differential permittivity arises fundamentally from the diffraction limit. Fortunately, the edge birefringence is usually weak and can be made weaker by matching the RI of the surrounding medium to the RI of the imaged specimen^39^. Edge birefringence also has a distinct feature of fast-varying orientation at the interface of two materials. Recognizing this, we computed the orientation continuity map described in Supplementary Note 4.2 to suppress this effect, as shown in Supplementary Fig. 1.

#### Verification of the PTI inverse algorithm

Next, we verified the PTI inverse algorithm by reconstructing the simulated Stokes volumes generated by the vector Born model (Fig. 2c,d). If the inverse algorithm is accurate, we would expect the reconstructed physical properties of various simulations to match the ground-truth physical properties in 3D space. We chose to generate the simulated data with the vector Born model instead of the most accurate FDTD simulator, because the vector Born model is >10,000× faster than FDTD and is almost as accurate as the FDTD simulator in the weakly scattering specimens as shown in Fig. 2b. We chose the simulation parameters that matched our experiments with an illumination NA of 1.4, objective NA of 1.47 and RI of the immersion medium (*n*_m_) of 1.515.

We first examined the reconstruction of PTI using a 3D spoke target with constant permittivity (*n*_o_ = 1.525 and *n*_e_ = 1.55, positive uniaxial) and varying 3D orientation as shown in Fig. 2c (top). Figure 2c (bottom) shows that the PTI inverse algorithm based on the linearized vector Born model works as expected. The reconstructed 3D mean permittivity and 3D differential permittivity show a blurred 3D spoke with slightly weaker mean and differential permittivity relative to the ground truth due to the diffraction-limited spatial resolution. The optic sign probability was accurate throughout the 3D spoke target. The 3D orientation of the ground truth target and the reconstruction was visualized in two separate images, in-plane orientation and out-of-plane tilt, as shown in Fig. 1d. According to the color of the spokes in these visualizations, the reconstruction shows consistent in-plane and out-of-plane orientation along each spoke as shown in the ground truth images. One major difference in the reconstruction was the hole at the center of the volume in the differential permittivity channel, where the ground truth image shows constant differential permittivity. This is because of the compensation of anisotropy within the diffraction-limited region, where the differential permittivity from the anisotropic material of varying orientation angles cancels out. The agreement between these two sets of images indicates that the inverse algorithm of PTI is accurate. Our simulation reproduces the compensation of anisotropy within the diffraction-limited region at the center of the volume in the differential permittivity channel, where the differential permittivity at varying orientations is superimposed, resulting in isotropic permittivity.

We verified the accuracy of the inverse algorithm by simulating 1-μm isotropic beads with varying average refractive indices and 1-μm anisotropic beads with varying differential permittivity with the vector Born model (Fig. 2d). The simulated data are processed with the PTI inverse algorithm based on the linearized vector Born model. We then plotted the reconstructed 3D mean permittivity and 3D differential permittivity against their ground-truth counterparts in the top and bottom of Fig. 2d. The reconstructions of 3D mean permittivity matched accurately with the ground-truth values, whereas the reconstructions of 3D differential permittivity slightly underestimated the correct values. The underestimation of the differential permittivity was due to the mismatch between the imaging model (vector Born model) and the inverse algorithm (linearized vector Born model), specifically due to the additional weak-object approximation in the inverse algorithm. In both figures, squares in red dashed lines indicate the range of the 3D mean permittivity and 3D differential permittivity reported in the experiments of this paper.

The above rigorous simulations establish quantitative bounds on the validity of our imaging model and inverse algorithm and clarify that the edge birefringence and compensation of differential permittivity arise from the diffraction of light.

### Evaluation of spatial resolution and accuracy

We verified the accuracy of four distinct volumetric measurements provided by PTI: mean permittivity, differential permittivity, 3D orientation and the optic sign in simulations shown in Fig. 2c,d. PTI is designed to achieve confocal-like depth sectioning in these measurements by using high NA partially coherent illumination and high-NA imaging. In this section, we verify the accuracy of PTI experimentally and characterize the diffraction-limited volumetric resolution achievable by PTI for these channels of information. We image three types of isotropic and anisotropic test targets. All measurements reported in this section are acquired with a 1.4-NA (NA_c_) oil immersion condenser and a 1.47-NA (NA_o_) oil immersion objective.

#### 3D imaging of anisotropic glass target

First, we imaged a laser-written anisotropic glass target shown in Fig. 3a (through-focus video is shown in Supplementary Video 2) to characterize the 3D orientation, verify estimation of optic sign and demonstrate the utility of PTI for metrology. The anisotropic target was made of fused silica modified with a polarized femtosecond laser focused with a 0.55-NA lens as noted in Methods (‘Specimen preparation’)^47^. With PTI, we identified two distinct laser-induced modifications: nanograting^48^ and nanopore^19^ at different axial layers of the material. Reading these two types of modifications along the depth was challenging with current methods, including QLIPP (Fig. 3c). According to previous work^19^, nanograting modification of the material generates negative mean permittivity and stronger differential permittivity, whereas nanopore modification generates positive mean permittivity and weaker differential permittivity. PTI estimates the target to have a high probability of being negative uniaxial material, which agrees with past observations^18,19^. The *x*–*y* and *x*–*z* sections through mean permittivity, differential permittivity and optic sign volumes matched the expected optical properties. As this is a negative uniaxial material, the 3D orientation of the symmetry axis reports the fast axis of the material. We show two components of the 3D orientation, in-plane orientation and the out-of-plane tilt, separately in Fig. 3a. The orientation of the symmetry axis in each spoke aligns with the *x*–*y* plane and is orthogonal to the direction of the spokes, which matches with the axis of symmetry expected from the state of laser polarization used in the writing process. In addition, we measured subtle non-uniformity in the mean and differential permittivity at the ends of the line features along each spoke (shown with arrows) caused by the variable dwell times used in the writing process.

**Extended Data Fig. 5:**
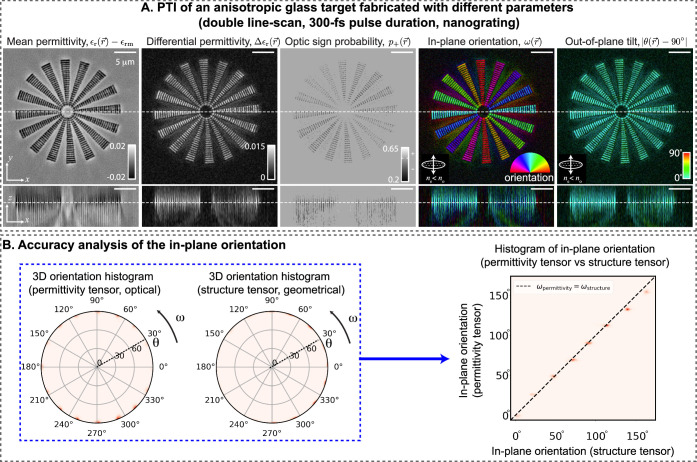
PTI can read the properties of anisotropic glass targets written with lasers with high resolution and sensitivity. **(A)** 3D mean permittivity, 3D differential permittivity, optic sign probability and 3D orientation (in-plane orientation and out-of-plane tilt) of a nanograting birefringent target that was written with 300-fs laser pulse duration, 515 nm wavelength, and illumination NA of 0.55 using a double line scan process. This process writes a feature by scanning the laser two times in opposing directions. Differences in the laser parameters relative to the target shown in Fig. 3 prevent the induction of two layers of modification as shown in Fig. 3a. In addition, the double line scan creates more uniform line features compared to Fig. 3a. Specifically, mean and differential permittivity are higher at the end of each spoke of the target shown in Fig. 3a, but not in this target. **(B)** We validate the accuracy of measured 3D orientation of PT by comparing it to the 3D orientation of the structure tensor computed from the variations in the differential permittivity volume. The structure tensor reports the 3D geometrical orientation of fine lines seen within spokes. The geometric orientation and the symmetry axis orientation of these lines co-align due to the writing process. This co-alignment is confirmed from the histograms of 3D orientation of permittivity tensor and structure tensor. Further, 2D histogram of the orientations of permittivity and structure tensors show linear dependence between these measurements. Source data

Another anisotropic target fabricated with different writing parameters is shown in Extended Data Fig. 5a (through-focus video is shown in Supplementary Video 3). This target has only one layer of nanograting modification. The uniform dwell times used for writing this target eliminated the non-uniformity. We segmented this target and compared the 3D orientation of PT with the 3D orientation of the local structure. Extended Data Fig. 5b shows the histograms of the 3D orientation of this anisotropic target from the PT and the structure tensor (Supplementary Note 4.3). The structure tensor captures the geometrical orientation of individual lines in each spoke, which should align with the symmetry axis of PT. We observed this match by plotting the histogram of the in-plane orientation from both tensors. As the spokes of both anisotropic targets have optic axes aligned in the *x*–*y* plane, we tilted the targets to characterize the accuracy of the out-of-plane tilt. Supplementary Fig. 2 shows the PTI reconstruction of the flat and the tilted anisotropic target in Extended Data Fig. 5. From the 3D orientation histogram of the orange box region, we observed that the tilt angle of the target estimated by PTI matched the tilt angle measured from the *x*–*z* section of the differential permittivity image.

The above measurements show that PTI can be a valuable technology for optical metrology and new optical storage, in addition to enabling new bioimaging.

#### Spatial resolution

**Fig. 5:**
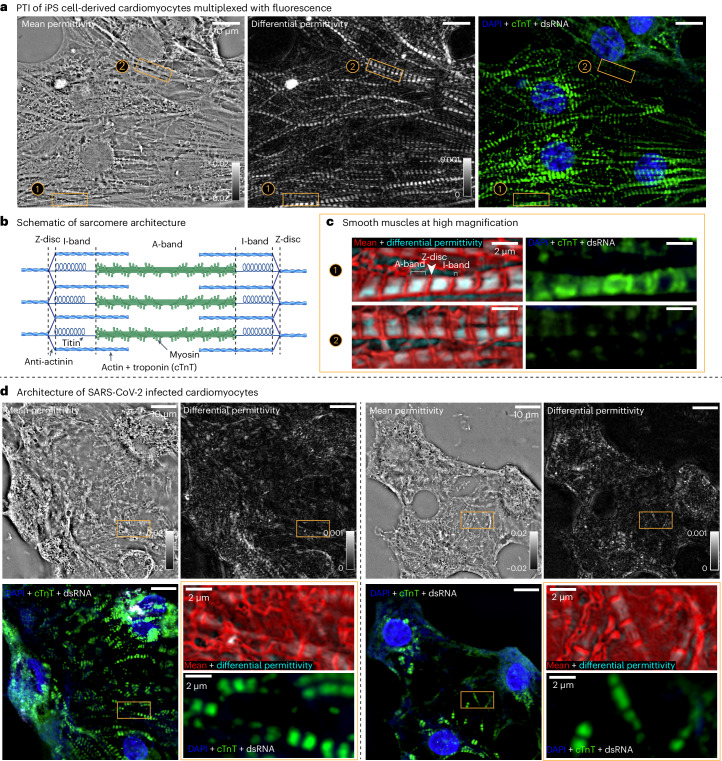
Imaging physical and molecular changes in architecture of iPS cell-derived CMs due to infection by SARS-CoV-2. **a**, 3D mean permittivity, 3D differential permittivity and fluorescence images (overlay shows DAPI stain in blue, cTnT stain in green and dsRNA stain in white) of the uninfected iPS cell-derived CMs. **b**, A schematic of the sarcomere architecture (created with BioRender.com) shows its key molecular components and their organization to enable interpretation of the images. **c**, Two zoomed regions of the iPS cell-derived CMs are shown with label-free channel (overlay of mean permittivity in red and differential permittivity in cyan) and the fluorescence channel. Z-disc, A-band and I-band can be identified in the label-free overlay. A-band and I-band are visible from variations in mean permittivity and differential permittivity and Z-disc is visible due to high mean permittivity and low retardance. In zoom (1), the cTnT label shows troponin in the actin-rich regions of the sarcomere, overlapping with both I-band and A-band. The zoom (2) shows weak fluorescence signal due to labeling stochasticity, but the sarcomeric architecture is visible in the label-free imaging. **d**, Two FOVs showing the same information as in **a** and **c** but for CMs infected with SARS-COV-2. The zooms of both FOVs show broken sarcomeres with label-free overlay and fluorescence. Relative to mock infection, the mean permittivity and fluorescence images show explosion of nuclei and the reduction in the differential permittivity in A-band indicates loss of myosin thick filaments. Source data

**Fig. 6:**
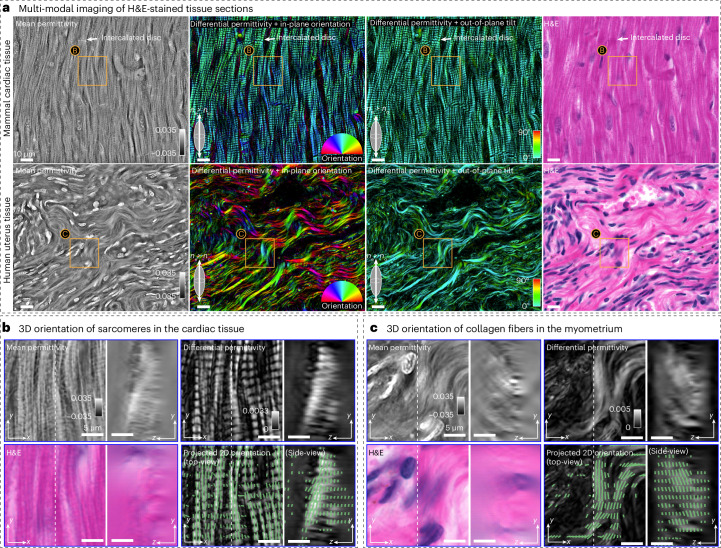
Imaging histological sections with PTI and H&E stain. **a**, 3D mean permittivity, 3D differential permittivity (color-coded with in-plane orientation and out-of-plane tilt) and H&E images of (top) the mammal cardiac tissue and (bottom) the myometrium region of the human uterus tissue. We used 770 nm wavelength for imaging H&E sections to avoid the strong absorption from the H&E stains. H&E images show histological structures such as nuclei in both tissues, collagen fibers in the uterus tissue, sarcomeres (z-discs, A-bands and I-bands) and intercalated discs (arrows). These structures are visible in mean permittivity and differential permittivity images at higher contrast and quantitative precision. The differential permittivity images specifically highlight anisotropic structures such as A-band of the sarcomere in the cardiac tissue and the collagen fibers in the uterus tissue. The 3D orientation (encoded by the colors in the differential permittivity images) clarifies how collagen fibers and sarcomeres are arranged. **b**, The orthogonal slices (*xy* and *yz*) of mean permittivity, differential permittivity, H&E and projected 2D orientation of the FOV indicated by the orange box in the cardiac tissue. The 3D orientation of the sarcomeres cannot be observed from the geometry of sarcomeres in H&E or mean permittivity images but is visible with PTI measurements of differential permittivity and projected orientation. **c**, The same information as in **b** is shown for the FOV indicated by the orange box in the uterus tissue. The 3D orientation of the collagen fibers cannot be observed through the geometry from the *y*–*z* sections but is measured and shown in the projected 2D orientation channel from PTI. Source data

Next, we characterize the spatial resolution of PTI by imaging 300-nm polystyrene beads with an RI of 1.5956 embedded in oil with an RI of 1.5536 (Fig. 3b). In the 3D mean permittivity image, we can resolve individual beads. In the 3D differential permittivity image, we can resolve the edge retardance of the beads in the form of small rings. We quantified the resolution using Gaussian fits in *x* and *z* directions to the mean permittivity of the beads in the center. Deconvolving the physical size of the bead from the fitted Gaussians, we obtained the shape of the point spread functions (PSFs) in the *x* and *z* directions. The full width at half maximum (FWHM) of the PSFs in the *x* and *z* directions show that we achieve a transverse FWHM of 230 nm and an axial FWHM of 810 nm. We used FWHM of a theoretical image of a point^4^ with a lens of 1.4 NA to benchmark the transverse and axial resolutions. The theoretical transverse FWHM was 190 nm (0.5 *λ*/NA) and the axial FWHM was 543 nm (2 *λ*/NA^2^). The theoretical axial resolution is a function of the transverse spatial frequency of the specimen^21^. For a spherical bead of finite spatial frequency, the FWHM is broader than the infinitesimal point. Our measured transverse FWHM and axial FWHM compared well with the theoretical estimates. These results also illustrate that our inverse algorithm and parameters do not introduce artifacts. As illustrated by Figs. 4–6, our measurements provided confocal-like 3D resolution that allows us to resolve cross-sections of single axons, bands of sarcomeres and intracellular features.

**Extended Data Fig. 6:**
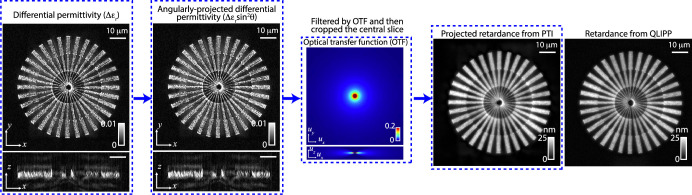
Computation of projected retardance from differential permittivity. The projected retardance is computed by projecting the differential permittivity on the focal plane (angular projection) and filtering the projected differential permittivity with the optical transfer function. The computed projected retardance matches with the retardance measured with QLIPP method. Source data

We compared the resolution of PTI with our previous method, QLIPP^27^. The 3D phase and projected retardance of the anisotropic glass target measured with QLIPP are shown in Fig. 3c with illumination NA of 0.7 and 1.4. The QLIPP’s circularly symmetric illumination aperture leads to weak phase contrast when the illumination NA matches the imaging NA. Therefore, Fig. 3c only shows QLIPP phase image of the target with illumination NA of 0.7. PTI mean permittivity image has a higher *xyz* resolution than QLIPP phase image as evident from the lines within each spoke of the target and the sharper features in the *x*–*z* section. The *x*–*z* sections of the 3D differential permittivity measured with PTI show a higher resolution than the projected retardance measured with QLIPP at both illumination NAs. PTI provides optical sectioning that distinguishes two layers of material modifications separated by 1 μm. The fine spacing (300 nm) inside individual spokes is better resolved in PTI than in QLIPP. Thus, anisotropy measurements with PTI approach the diffraction limit. It is worth noting that the 3D differential permittivity from PTI is dimensionless (Δ*ϵ*_r_) and the projected retardance from QLIPP has the dimension of a length. When projecting the reconstructed 3D differential permittivity angularly and spatially as shown in Extended Data Fig. 6, we can convert it into the projected retardance measured by QLIPP. Thus, 3D differential permittivity from PTI is a more accurate measure of material permittivity than achievable by QLIPP.

#### Accuracy

**Extended Data Fig. 7:**
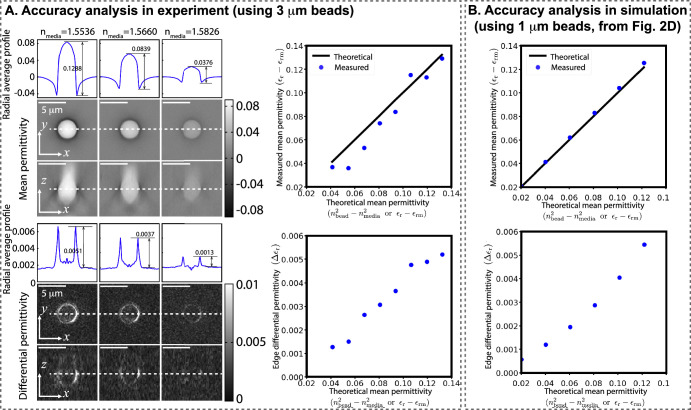
Experimental evaluation of the accuracy of mean and differential permittivity measurements. **(A)** We experimentally characterized the accuracy of mean and differential permittivity measurements by imaging 3 μm polystyrene beads immersed in oils with varying refractive indices (RI). The cross-sections through the volumes of mean and differential permittivity, and corresponding radial average profiles show that the measured permittivity decreases as the RI of the immersion oil approaches the RI of the bead. Plots of measured mean permittivity and measured differential permittivity versus the theoretical mean permittivity (*n*_beads_ = 1.5956 at 532 nm) show that the measured permittivity follows the expected trend. **(B)** Corresponding plots of measured mean permittivity and differential permittivity at the edge vs. theoretical mean permittivity from the simulation of 1 μm beads shown in Fig. 2d. The comparison of the measured and theoretical mean permittivities establish the accuracy of the measurement, whereas comparison of the differential permittivity and mean permittivity establishes relative strengths of differential permittivity of the isotropic edge.

Finally, we characterized the accuracy of mean and differential permittivity by imaging isotropic 3-μm polystyrene beads embedded in oils of varied RIs as shown in Extended Data Fig. 7a. Embedding the beads (*n*_beads_ = 1.5956) in the medium of varying RI (*n*_media_ ranges from 1.5536 to 1.5826) changes the accumulated optical path length (theoretical phase) of the light as well as the amount of edge retardance^46^ linearly. When the RI of the surrounding medium is the same as the RI of the beads, there will be no accumulated phase and edge retardance. Such an embedding series allows us to characterize the linearity of phase and differential permittivity measured with PTI. In the mean permittivity images of the beads, 3-μm spheres are well reconstructed to have the expected shapes except for the elongation in the *z* direction and the negative bias around spheres. The elongation in *z* arises from the non-isotropic 3D spatial resolution as characterized in Fig. 3b. The negative phase bias arises due to the lack of sensitivity to the slowly varying phase when imaging with partially coherent illumination (see Discussion). We also observed both effects in our phase image of the simulated bead in Extended Data Fig. 4b. The mean permittivity of the whole bead and differential permittivity at the edge dropped linearly as the RI of the immersion oil approached the RI of the bead. Plotting the measured permittivity against the known permittivity of the beads relative to oils (${n}_{{{{\rm{beads}}}}}^{2}-{n}_{{{{\rm{media}}}}}^{2}$) showed a good quantitative match. We also saw that edge permittivity varies linearly with the relative permittivity of the bead, which is in agreement with the measurements of the edge retardance reported previously^46^.

Finally, we compared the above experimental observations with corresponding simulations in Extended Data Fig. 7b by extending the simulations shown in Fig. 2d. We show reconstructed mean and differential permittivity of 1-μm beads of increasing RI versus the permittivity of the bead relative to oil. Notably, our simulation not only shows that the mean permittivity measurements are accurate but also reports the same amount of differential permittivity as in the experiment. As the 3D differential permittivity and the 3D mean permittivity both have the dimensions of the relative permittivity, we can compare them quantitatively. From the above experiment and the simulation, we observe that the differential permittivity of a transparent edge is about an order of magnitude smaller than the mean permittivity of the material.

### Multi-scale analysis of brain tissue architecture with PTI

A key limitation of current polarized light microscopy^27,28,44^ approaches has been that their light paths are not sensitive to the inclination of the 3D anisotropy. As a result, they report anisotropy projected on the microscope’s image plane. Polarization microscopy with scanned illumination aperture^49^, tilted specimen stage^50^ and light-field^51^ detection are sensitive to the inclination but do not have the diffraction-limited resolution, because they do not account for diffraction effects. Here, we report volumetric measurements of the 3D orientation of biological structures with diffraction-limited resolution.

The architectural connectivity of mammalian brains can be inferred from the spatio-angular distribution of myelinated axons. The myelin sheath is composed of multiple lipid bilayers and wraps around axons. MRI can provide measurement of spatio-angular distribution of axon bundles^42^ and myelin fraction^52^ with millimeter resolution; however, inference of the connectivity or pathology frequently requires micro-architectural ground truth^52,53^. Polarization microscopy is emerging as a label-free method for analyzing mesoscale connectivity and the architecture of brain tissue^13–16,27,54^, because the high anisotropy of the myelin sheath enables sensitive detection of distribution and orientation of axon fibers^55,56^ and visible light microscopy can achieve submicron, single-axon resolution across large brains. Quantitative phase microscopy has also enabled imaging of brain architecture^57,58^.

We reasoned that measurement of differential permittivity and 3D orientation at diffraction-limited resolution could reveal the architecture of the brain tissue. As Fig. 1b illustrates, the lipid bilayer has a higher RI perpendicular to the RIs in the plane of the bilayer (it is a positive uniaxial material). When the light scattered by the myelin sheath is integrated around the axon cross-section, the ensemble RI is higher along the length of the axon relative to the RIs in the cross-section of the axon (the whole axon is a negative uniaxial material). The myelination in brain tissue can be measured from the differential permittivity. The 3D orientation measured at the resolution of the diameter of single axons (~1 μm) can enable analysis of the complex connectivity within brain regions. Here, we report measurements of mean permittivity, differential permittivity and 3D orientation at spatial scales ranging from 1 cm to 1 μm in 12-μm thick sections of brain slices. At high resolutions, we acquire volumetric measurements and at low resolutions, we acquire planar measurements.

#### 2D imaging of whole section

First, we report planar (2D) measurements of a section of adult mouse brain tissue. Figure 4a–c show the 2D mean permittivity, 2D differential permittivity and 3D orientation (in-plane orientation and out-of-plane tilt) of an adult mouse brain located at level 51 of the Allen brain reference atlas (https://mouse.brain-map.org/static/atlas). With the imaging and illumination NA of 0.55 (corresponding to the spatial resolution of ~0.5 × 0.5 × 3.2 μm), the imaging system measures anisotropy of myelin sheath averaged over whole axons. As a result, axons behave like a negative uniaxial material (Supplementary Fig. 3b) with 3D orientation (Fig. 4b,c) co-linear with the axon axis^55,56^. Therefore, we assume that all the axons are negative uniaxial material when computing the 3D orientation at this resolution. The 3D orientation is rendered in two separate images with the brightness encoding the 2D differential permittivity and the color encoding the in-plane orientation and out-of-plane tilt as indicated by the color wheel and color map. Mean permittivity shows the overall morphology of the mouse brain, whereas differential permittivity highlights the distribution of myelinated axons. As in other work^27,54^, important anatomical regions such as anterior commissure olfactory limb (aco), corpus callosum (cc), caudoputamen (CP), cortex (CTX) and ventricle (VL) are visible in both 2D mean permittivity and 2D differential permittivity. In Fig. 4b,c, we not only see the in-plane orientation aligned with the axon bundle, but also see that the left and right anterior commissure olfactory limb are inclined relative to the microscope axis (yellow-colored stretches in Fig. 4c indicated by bottom two white arrows in Fig. 4b). The same out-of-plane tilts are also visible in yellow and red colored stretches at aco when the 3D orientation is encoded using the 3D color sphere (Supplementary Fig. 3a).

#### Volumetric, high-resolution imaging of brain regions

Next, we report a high-resolution analysis of the brain tissue. Figure 4d shows *x*–*y* and *x*–*z* sections of the aco region imaged at high-resolution (1.47 NA, spatial resolution of ~0.23 × 0.23 × 0.8 μm) in the section described above. Corresponding scans through *x*–*y*, *x*–*z*, *y*–*z* sections are shown in Supplementary Video 5. At high resolution, PTI measurements resolve myelin sheath around individual axons, which behaves like a positive uniaxial material with 3D orientation normal to the membrane. 3D orientation visualized with the 3D color sphere and the optic sign probability are shown in Supplementary Fig. 3c. In this field of view, longitudinal and cross-sections of axons are visible in both mean and differential permittivity channels, suggesting axons have a wide 3D angular distribution.

**Extended Data Fig. 8:**
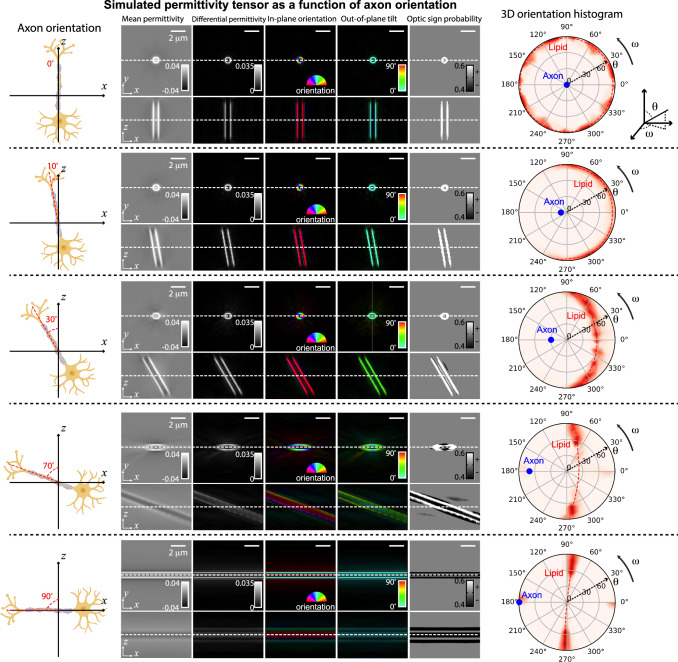
Sensitivity of measured uniaxial permittivity tensor to inclination of anisotropic material. Simulated reconstruction of the uniaxial permittivity tensor components for single axons tilted with 0^∘^, 10^∘^, 30^∘^, 70^∘^, 90^∘^ from *z*-axis to the left. In this simulation, we assume an axon is a perfect cylindrical shell composed of uniform positive uniaxial material with radially arranged 3D orientation around the cylinder axis. The reconstruction (3D mean permittivity, 3D differential permittivity, 3D orientation and optic sign) generally performs better when axons incline slightly from *z*-axis, corresponding to the case when the 3D orientation of lipid molecules in axon boundaries lies within the *x*–*y* plane. The histograms of 3D orientation of the axon boundaries (lipids) shows a circular shape when the axon is oriented along *z*. The 3D orientation of axon boundaries (lipids) shifts gradually towards the pole of the top hemisphere as the axon inclines towards the *x*-*y* plane. The 3D distribution gets distorted when axon inclination is larger than about 70^∘^ or when inclination of axon boundaries (lipids) is less than 20^∘^, because of poorer sensitivity of our method to the anisotropy aligned with the microscope axis.

We check the consistency of the measurements of the 3D orientation of the differential permittivity of lipids by comparing it with the 3D orientation of the structure tensor of the 3D mean permittivity (Supplementary Note 4.3). Figure 4e shows the histograms of the 3D orientation of the differential permittivity of lipids and structure tensor in two subvolumes. The azimuth dimension of the histogram shows the in-plane orientation, *ω* and the radial dimension shows the inclination relative to the imaging axis, *θ*. The green box contains axons mostly inclined at 60° to the left of the *z* axis and the red box contains axons mostly inclined at 20° to the left of the *z* axis. We indicate the axon orientation with blue dots in the histograms of 3D orientation. When the axon is aligned (0°) relative to the *z* axis, we expect 3D orientation of lipids evenly distributed in the focal plane, which will be a distribution around a circle with radius *θ* = 90°. With 20° and 60° inclinations of axons, we expect a gradual rotation of this circle (collective 3D orientation of lipids) to the left side of the histogram, which is what we observed in 3D orientation histograms from both the PT and the structure tensor. At high inclinations of the axon, we notice a gradual reduction in the density of orientations of lipids as lipids align along the *z* axis. This drop in sensitivity is due to the weaker transfer of the differential permittivity to intensity modulations as the material aligns with the *z* axis. We verify these observations further by replicating them in PTI simulations with axons of increasing inclination angles in Extended Data Fig. 8.

Measurement of differential permittivity with PTI reveals anisotropic structures that are oriented toward the imaging axis. In traditional polarization microscopy and our design, QLIPP, the measured retardance reports a mixture of true anisotropy and inclination angle. When the anisotropic material is more aligned to the imaging axis (here, the *z* axis), the projected retardance measurement is smaller. PTI reports the differential permittivity that is independent of the inclination angle. As can be seen from the overlay (Fig. 4f) of the angularly projected differential permittivity (in magenta) and the differential permittivity (in green) of the subvolume in Fig. 4d, measuring the differential permittivity enables more accurate visualization of the distribution of axons.

**Extended Data Fig. 9:**
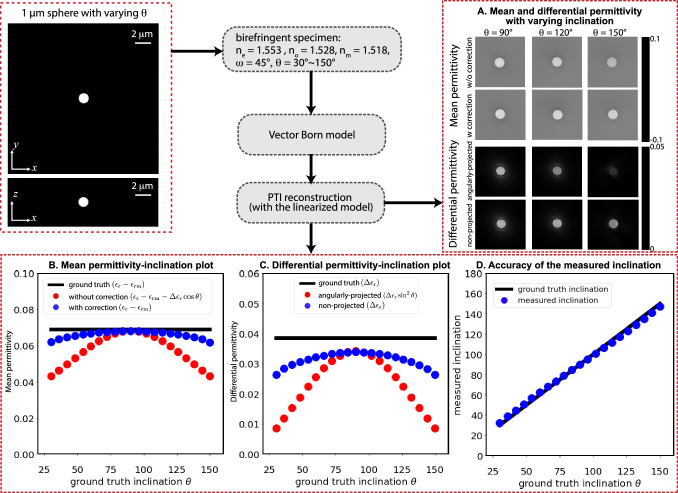
The effect of inclination angles on the accuracy of mean and differential permittivity. PTI reconstruction of simulated data from a 1-μm sphere with extraordinary RI *n*_e_ = 1.553, ordinary RI *n*_o_ = 1.528, in-plane orientation *ω* = 45^∘^, and varying inclination angles *θ* = 30^∘^ ~ 150^∘^ immersed in media with RI of *n*_m_ = 1.518. **(A)** The reconstructed mean and differential permittivity images (with and without inclination correction) of simulated specimens with varying inclination angles. **(B)** Quantitative analysis of the measured mean permittivity with and without the inclination correction compared with the ground truth under various inclination angles. **(C)** Same quantitative analysis for the measured differential permittivity with and without inclination correction. **(D)** The accuracy analysis of the measured inclination angles in these simulations. When correcting inclination effects (enabled by PTI) in mean and differential permittivity, we found better matches of the values to the ground truth mean and differential permittivity.

To further quantify the accuracy of PTI measurements as a function of the inclination of an anisotropic object, we reported simulations of anisotropic 1-μm beads with varying inclination angles. Extended Data Fig. 9a shows the mean and differential permittivity images with and without the correction for inclination angle. Extended Data Fig. 9b,c plots these measurements and the ground truth versus the inclination angles. We also verified the accuracy of the measured inclination angles in Extended Data Fig. 9d. These simulations show that PTI provides more accurate mean and differential permittivity measurements by eliminating the effect of the inclination; however, this correction is less effective as the anisotropic material orients toward the imaging axis. This is because of the effect of the regularization term included in the inverse algorithm. We trade off the accuracy of PTI measurements of the structures oriented along the imaging axis in favor of robustness against noise by choosing a nonzero regularization parameter.

Relative to QLIPP and analogous polarization methods, PTI enables high-resolution imaging of axon networks from their diverse physical properties due to the illumination diversity in measurement, linearized vector Born model and the multi-channel inverse algorithm. As pointed out previously, Supplementary Fig. 4 shows that the projected retardance of the mouse brain section from QLIPP can be obtained through angular and spatial projection of the 3D differential permittivity measurement from PTI. Figure 4g shows QLIPP measurements of the 3D projected retardance and the histogram of corresponding 2D orientation measurements for the green and the red boxes shown in Fig. 4d. Axon boundaries are barely visible in the QLIPP measurements due to lower resolution and contrast. The inclination and optic sign are measurable with PTI, but not with QLIPP.

**Extended Data Fig. 10:**
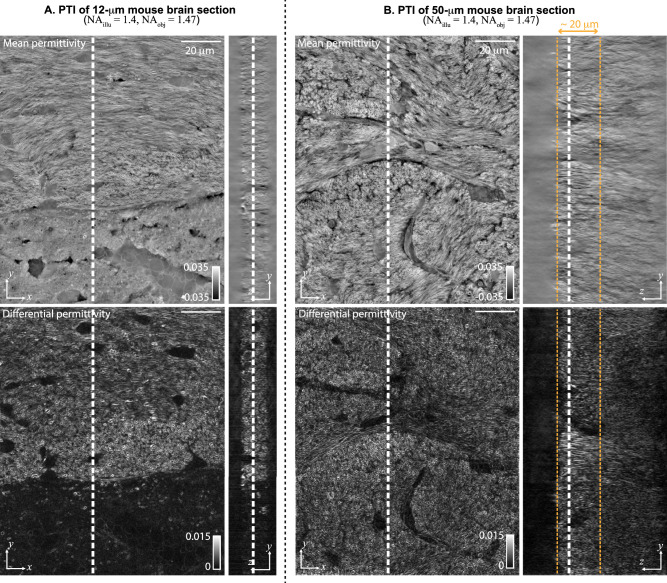
Validity of the first Born approximation in mouse brain tissues. *x*-*y* and *y*-*z* sections of 3D mean permittivity and 3D differential permittivity of **(A)** 12-μm and **(B)** 50-μm mouse brain sections. Weak- and single-scattering (first Born) assumptions gradually fail as 3D mean permittivity and 3D differential permittivity become dimmer and blurrier beyond the imaging depth of 20 μm in a 50-μm thick mouse brain tissue slice. Source data

We also characterized the imaging depth of PTI at high resolution (NA_illu_ = 1.4 and NA_obj_ = 1.47) using mouse brain sections of two different thicknesses. Extended Data Fig. 10a,b shows the 3D mean permittivity and 3D differential permittivity images of a 12-μm (from Fig. 4d) and a 50-μm mouse brain section, respectively. From the *y*–*z* section of Extended Data Fig. 10b, we observe that the mean and differential permittivity images start to become blurrier and dimmer beyond the imaging depth of 20 μm in the 50-μm tissue. This is because the multiply scattered photons dominate in the thick tissue. The contrast introduced by these photons is not captured by our linearized vector Born model. This not only demonstrates the limit of our vector Born model in handling multiple-scattering specimens but also illustrates that the reconstruction quality degrades gracefully as the first Born approximation becomes inaccurate. The data reported from other experiments in this paper do not suffer from degradation of image quality due to multiple scattering.

#### Multi-resolution analysis

Finally, we automated multi-scale PTI imaging of millimeter-sized tissue sections with submicrometer 3D resolution. We automated tiled acquisition using Micro-Manager (https://github.com/micro-manager), a Python bridge to Micro-Manager (https://github.com/czbiohub-sf/mm2python) and a GPU-accelerated computational pipeline implemented on a compute cluster as described in Methods (‘Multi-scale imaging and analysis’). We designed the analysis pipeline to enable robust reconstruction of PT at any scale spanned by the acquisition. Measurements at larger scales (lower resolution) were computed by a spatially filtering approach (Supplementary Note 4.1). Results of one such multi-scale analysis of the right corpus callosum region are shown in Supplementary Video 4. At spatial scales larger than the typical size of axons, we computed the 3D orientation assuming a negative uniaxial material. When axon cross-sections were resolved, we visualized complex axon networks by displaying the mean and differential permittivity through focus and at multiple locations.

We verified the quantitative correspondence between 3D orientation distributions measured with low-resolution (×20, 0.55 NA) and high-resolution (×63, 1.47 NA) acquisitions. We imaged the 3D orientation in Fig. 4d at high-resolution, low-pass filtered the high-resolution data to have a similar spatial resolution as the low-resolution data and computed the 3D orientation histogram within two subregions as shown in Supplementary Fig. 5. The histograms of 3D orientation of axon bundles in the low-resolution data and the smoothed 3D orientation computed from high-resolution data agreed well, confirming that our pipeline provides physically meaningful measurements across spatial scales. These results also indicate that PTI enables the sensitive measurement of 3D anisotropy that cannot be resolved from the spatial architecture. As a result, PTI with 1-μm resolution can be used for rapid, quantitative analysis of the distribution of axons in different regions of the brain.

### Correlative PTI and fluorescence imaging of infected cells

Quantitative label-free imaging provides unbiased and consistent readouts of the physical architecture of diverse cell types, including human cells and tissues. Immunolabeling, on the other hand, provides complementary information about the distribution of specific molecules. To map the both the physical and molecular architecture of cells at the confocal-like 3D resolution, we designed and implemented PTI as a module that is easily multiplexed with other wide-field imaging methods. PTI’s design permits the use of the highest NA illumination and imaging lenses, which allows us to achieve diffraction-limited 3D resolution. We used PTI multiplexed with fluorescence to analyze cytopathic effects in two cellular models of infection, SARS-CoV-2-infected iPS cell-derived CMs (discussed in this section) and RSV-infected A549 cells (discussed in Supplementary Note 7). We demonstrate that PTI can reveal impacts of perturbations such as infection at cellular and organelle scales.

iPS cell-derived CMs have emerged as genetically editable models of cardiac diseases and drug screening^59^. CMs are highly specialized contractile cells. Studying the architecture of the myofibril and its building blocks, the sarcomeres, is of critical importance to characterize their function^59^. Polarized light imaging has played an important role in understanding architecture and activity of sarcomeres^60^ (Fig. 5b). In fact, the A- and I-bands of sarcomeres were named after the anisotropic and isotropic bands first observed in muscle tissue with polarized light microscopy^61^.

Figure 5a and the corresponding through-focus video Supplementary Video 6 show label-free (3D mean permittivity and 3D differential permittivity) and fluorescence images of fixed iPS cell-derived CMs acquired with a 1.4-NA (NA_c_) oil immersion condenser and a 1.47-NA (NA_o_) oil immersion objective. The mean permittivity image shows nuclei, myofibrils and a crowded meshwork of membranous organelles surrounding sarcomeres. The differential permittivity shows the distinct striated pattern of myofibrils. The CMs are stained with 4,6-diamidino-2-phenylindole (DAPI) (blue) to label chromatin and fluorescent antibody (green) against thin filament marker cardiac Troponin T (cTnT) to label sarcomeres. We see the locations of the nuclei and sarcomeres agree between the label-free and the fluorescence channels; however, the differential permittivity image shows consistent periodic sarcomere organization that is not always captured by cTnT labeling, especially for region of interest (ROI) ②. It is worth noting that the differential permittivity values are about 20 times lower than the mean permittivity values. This suggests that the differential permittivity of the sarcomeres could be masked by the edge retardance of the mean permittivity. To suppress the edge retardance, we applied the orientation continuity map described in Supplementary Note 4.2 and obtained clean measurements, as demonstrated in Supplementary Fig. 1b.

The schematic in Fig. 5b shows sarcomere architecture and its key molecular components to enable interpretation of the images. Each sarcomeric unit is bracketed by two Z-discs, composed of densely packed proteins including *α*-actinin. Between Z-discs, the sarcomere is organized in an I-band (isotropic band) and an A-band (anisotropic band). An I-band is mainly composed of thin actin filaments, while an A-band contains thick myosin filaments. The myosin filaments in the A-bands contain bound electrons more easily polarized (higher RI) along the filaments, resulting in an angular RI distribution of a positive uniaxial material. Supplementary Fig. 6a shows the 3D orientation and optic sign probability of the corresponding field of view. The 3D orientation aligns well along with the orientation of myofibrils. The optic sign probability suggests that thick filaments in the sarcomere behave as a positive uniaxial material, which matches their molecular structure.

We zoomed in on two regions of Fig. 5a to examine sarcomeric structures in Fig. 5c. We displayed the label-free channels of these two ROIs with an overlay of mean permittivity in red and differential permittivity in cyan, from which we can clearly resolve sarcomeric components. The mean permittivity channel emphasizes the electron-dense Z-disc region. In between Z-discs, we saw both strong mean and differential permittivity that arose from the anisotropic thick myosin filaments, which defined the A-band. We further noticed spacing between Z-discs and A-bands. This spacing had lower mean permittivity and almost no differential permittivity (it was less dense and nearly isotropic). Comparing its location and size with the transmission EM images of CMs^62^, it was identified as the I-band. Figure 5c also shows corresponding fluorescence images for the same ROIs. As the cTnT is localized in both I-band and A-band, we saw most of the signal between two Z-discs in ROI (1) of the fluorescence image. cTnT labeling in ROI (2) does not detect sarcomeres, while label-free channels detect sarcomeres. These data suggest that the label is missing due to the inaccessibility of cTnT to antibodies or the mis-localization of cTnT. Here, label-free imaging complements the inconsistent immunostaining by providing consistent physical measurements of sarcomeres.

iPS cell-derived CMs have been shown^62^ to recapitulate cytopathic effects of COVID-19 in the autopsy specimens, even though the virus was not detected in the autopsy sections. The noteworthy phenotypes discovered from these studies are fragmentation of myofibrils and loss of chromatin stain. We multiplexed label-free and fluorescence measurements of the SARS-CoV-2-infected CMs in Fig. 5d and corresponding through-focus videos (Supplementary Video 7 and Supplementary Video 8). The infected cells are recognized by immunostaining double-stranded RNA (dsRNA), a unique signature of a replicating virus. Here we show two distinct fields of view (FOVs) of the infected cells from the same coverslip. In the left FOV of Fig. 5d, we see a substantial reorganization of CMs around the nucleus in both label-free and fluorescence channels. In the fluorescence image, the dsRNA signal is visible in the perinuclear region of this cell, indicating replication of the virus through the endoplasmic reticulum–Golgi system. Multiple fragmented myofibrils are visible in our data, especially from the cTnT label, as also reported previously^62^. In both mean permittivity and differential permittivity, myofibrils are much less visible, indicating the loss of integrity of sarcomere architecture. In particular, a large reduction in differential permittivity suggests the loss of thick filaments in the A-band. Our data agree with a report that outlines myosin cleavage by a SARS-CoV-2 viral protease^63^. Supplementary Fig. 6b shows the 3D orientation and optic sign probability of the corresponding FOVs. The reduced anisotropic signal leads to higher noise in reading 3D orientation and the optic sign prediction; however, we detected pieces of broken sarcomeres with the parallel orientation and small patches of the positive optic sign.

These results show that complementary information can be gained in the architecture of cardiac cells and tissues using PTI multiplexed with fluorescence imaging. The ability to resolve Z-discs and small I-bands further illustrates the high resolution and sensitivity of the PTI, establishing it as a promising method to phenotype sarcomeric structure, maturity and cytopathic effects. The image-based phenotyping can be valuable for modeling sarcomeric cardiomyopathies, screening cardiotoxic or cardioprotective drugs, or developing methods to improve iPS cell CM maturity. It also can be applied to other valuable muscle specimens that are challenging to label, such as primary cardiac or skeletal myocytes or for the non-disruptive imaging of sarcomeric architecture in live muscle cells without the need of engineering fluorescent reporter cell lines. In Supplementary Note 7, we further demonstrate the phenotyping capability of PTI multiplexed with fluorescence imaging in identifying architectural changes of A549 cells due to the RSV infection. Collectively, these results show that new insights can be gained in the architecture of infected cells using PTI multiplexed with fluorescence imaging, which opens new opportunities for image-based disease phenotyping and studies of multiple infectious diseases.

### PTI of H&E-stained histological sections

Microscopic imaging of H&E-stained histological sections has been the gold standard in the diagnosis of many diseases. Because of their utility, pipelines to generate these sections are well established. Using PTI to image already available H&E-stained specimens can enable richer image-based diagnosis and even virtual staining^27^ of unlabeled tissue. We demonstrate this capability by multiplexing PTI with H&E images on two off-the-shelf H&E specimens (Carolina Biological Supply).

For this experiment, we chose the 770 nm wavelength for PTI imaging and imaged the specimens with red (635 nm), green (525 nm), blue (470 nm) light separately to synthesize H&E images with proper white balance^64^ (NA_c_ = NA_o_ = 1.2). The imaging model of PTI assumes weak light-matter interactions to simplify the recovery of the physical properties of the specimens. In the visible spectrum, H&E-stained sections demonstrate strong absorption. We found that imaging at 770 nm avoided the strong absorption and thus avoided any model mismatch.

Figure 6a and the corresponding through-focus videos (Supplementary Videos 9 and 10) show images of mammal cardiac tissue and human uterus tissue (at the myometrium) with PTI and the H&E channels. Similar to Fig. 5, in the cardiac tissue, we observe the electron-dense Z-discs of the sarcomeres and nuclei in the mean permittivity channel and the anisotropic A-bands of the sarcomeres in the differential permittivity channel. Moreover, tissue-scale architecture, unlike cell-scale architecture, shows that bundles of sarcomeres are grouped with the electron-dense cardiac-muscle-specific intercalated discs that show strong mean permittivity but low differential permittivity (arrows). In the uterus section, we observe electron-dense nuclei and the collagen fibers in the mean permittivity channel and, specifically, the anisotropic collagen fibers in the differential permittivity channel. Collagen proteins polymerize to form triple helical fibers. The bound electrons of a collagen fiber are more easily polarized along the fiber direction than the radial direction, resulting in an angular RI distribution of a positive uniaxial material. All these structures are also visible in the H&E images with a blue color referring to the nuclei and the red color referring to protein structures such as sarcomeres and collagen fibers. Each imaging mode provides complementary views of the tissues with molecular and physical specificity. In addition to the mean and differential permittivity, PTI measures 3D orientation and the optic sign (Supplementary Fig. 7a). These measurements show that both the thick myosin filaments (A-band of the sarcomere) and the collagen fibers are positive uniaxial materials with the optic axes aligned with the long axes of the fibers, which match their molecular structures.

To better visualize the 3D orientation, we zoomed in on the orange box regions of the cardiac tissue and the uterus tissue and showed the *x*–*y* and *y*–*z* sections of 3D mean permittivity, 3D differential permittivity, H&E and 3D orientation in Fig. 6b,c. Here, 3D orientation was projected on the plane of viewing and shown by lines. The *y*–*z* sections of mean and the differential permittivity of both tissues showed good sectioning that enabled identification of the tissue layers. The differential permittivity of Fig. 6b shows that the sarcomeres are oriented north–south with a small tilt from the focal plane. The 3D orientation visible from the shape of sarcomeres matched well with the two projected views of the 3D orientation measurements from the PT. For the uterus section, the differential permittivity of Fig. 6c did not provide sufficient resolution to visualize 3D orientation purely from the shape of fibers. Fortunately, in this case, the 3D orientation of the PT reports the collective orientation of subresolution collagen fibers. Supplementary Fig. 7b,c shows the optic sign probability maps for the same zooms on these tissues. The orientation of collagen fibers is an important prognostic indicator of human breast cancer^65^. We envision this information being potentially useful in cancer diagnosis.

We have demonstrated that PTI is compatible with the H&E-stained tissue sections and provides complementary information regarding the physical properties of the tissues.

## Discussion

We have demonstrated that PTI provides a complete measurement of the uniaxial PT of specimens compared to previously reported quantitative phase and polarization microscopy methods. We have systematically verified the accuracy of the imaging model and the inverse algorithm. We have illustrated the broad utility of PTI by analyzing the architecture of laser-written anisotropic glass, isotropic glass beads, mouse brain tissue sections, cells infected with respiratory viruses and H&E-stained histological sections. We also illustrated how the PT can be interpreted in terms of the physical properties of the specimen. We have implemented automated acquisition and analysis to enable multi-resolution analysis of tissue architecture. We have also developed a video abstract (Supplementary Video 13) to illustrate the PT of a specimen, image acquisition process and the inverse algorithm. Next, we described how we chose to balance the trade-offs among spatial resolution, temporal resolution, sensitivity and complexity when designing and implementing PTI. We also discussed the future directions of research enabled by this work.

Volumetric analysis of the architecture of the mouse brain tissue, CMs, A549 cells and H&E tissue sections illustrates that a high-NA implementation of PTI can measure density and 3D anisotropy with confocal-like resolution, which has been challenging previously. The physical architecture accessible with PTI complements the molecular architecture that can be imaged with multiplexed fluorescence. The sensitivity and resolution of our data indicate that the measurements provided by PTI can enable new studies in demyelinating diseases, changes in organelle architectures of infected cells, mechanobiology, pathology and other fields. Measurements of 3D orientation and differential permittivity at high spatial resolution can provide new quantitative insights into the mechanobiology of polymeric cellular assemblies, such as myofibrils. Our measurements provided 3D volumes of mean permittivity, differential permittivity and 3D orientation. We have employed PTI to characterize laser-written anisotropic glass, which is a rapidly emerging high-density optical storage technology. PTI can therefore provide a foundation for developing readers of such optical storage devices. While label-free imaging is particularly suitable for live cell imaging, the speed and sensitivity of PTI need to be improved further to enable live PTI as discussed next.

PTI achieves high transverse and axial resolution using high-NA partially coherent illumination. The partially coherent illumination can provide 2× higher resolution than methods that use coherent illumination. Synthetic aperture imaging with coherent illumination^29,41^ can approach the same resolution; however, the partial coherence makes the measurements robust to speckle noise that commonly affects label-free imaging methods^66^. Our measurements are of high resolution, even in tissues up to 20-μm thick, as shown in Extended Data Fig. 10b, because they are not corrupted by speckle contrast. Relative to the interference-based optical designs, our non-interferometric design achieves better robustness to speckle noise by trading off the sensitivity to low spatial frequencies (large-scale variations) in the mean permittivity channel. PTI has high sensitivity for low spatial frequencies of differential permittivity. PTI detects most of the mid-to-high-spatial frequency features of interest, for example, axons, collagens and cellular organelles as reported by the data in this paper. Another advantage of our non-interferometric design is that PTI is robust to the phase-wrapping issue in interference-based methods. This is because 3D mean permittivity distribution is linearly related to the 3D distribution of the acquired intensity in our model. When the dry mass of the specimen becomes too strong to wrap in a normal interference-based method, our phase reconstruction will still be monotonic but less accurate, ensuring robust analysis. Finally, the partially coherent design simplifies the opto-mechanical implementation. PTI employs simpler hardware relative to the existing label-free microscopy methods that report both density and anisotropy^20,28–30,41,44^, yet provides more complete and quantitative measurements.

The simpler optical design makes it easy to multiplex PTI with other wide-field imaging modalities, such as fluorescence (as demonstrated), H&E staining (as demonstrated) and spatial transcriptomics. PTI can be implemented on a commercial microscope by adding an LCD panel, a circular polarizer and a machine-vision polarization camera. This design eliminates the need to tilt or rotate the specimens. In the present implementation, PTI is too slow to enable live imaging because of the slow refresh rate and the slow software communication of the Adafruit LCD; however, faster electronic control of phase and polarization diversity and a more advanced LCD panel (for example, transmissive spatial light modulator) will enable rapid acquisition that enables imaging of live cells and tissue. PTI detects polarization-sensitive modulations with a compact polarization camera. Our current choice of using a machine-vision polarization camera enabled a simple setup and robust calibration, but it is less sensitive to small changes in retardance as polarization imaging based on elliptical states^25,27^. PTI can be extended to utilize elliptical states, leading to higher sensitivity to small changes in the anisotropy of biological or fabricated specimens.

Label-free channels measured by PTI are affected to varying degrees by noise. In particular, the estimations of the optic sign and the inclination of the 3D orientation become unstable when the symmetry axis is aligned with the imaging axis. This occurs because imaging with a single lens cannot probe the specimen changes in the polarization states aligned with the imaging axis. Nevertheless, we show that the high-NA illumination version of PTI provides sufficient sensitivity to map the inclination of axons in mouse brain tissue and enables estimation of optic signs in 2D or 3D space (Supplementary Fig. 3). As far as we know, estimating the optic sign in 2D or 3D space has not been feasible with earlier methods. The robustness of inclination and the optic sign can be improved using elliptical polarization states that reduce noise and using multiview imaging.

Spatio-angular measurements of biological systems is a rapidly growing field. Spatio-angular measurements akin to PTI are being developed with fluorescence polarization imaging^37,67–69^. We anticipate synergies between the algorithms that we have reported, algorithms developed for DTI and algorithms developed for fluorescence polarization imaging. The key aspects of our current inverse algorithms that we aim to improve are (1) using a calibrated imaging pupil, akin to using calibrated polarization response for accurate imaging in the presence of aberrations; (2) reducing the number of regularization parameters to make the inverse algorithm more user-friendly and make it easier to obtain reproducible reconstructions; and (3) extending the model to enable imaging of thicker biological specimens that multiply scatter the light. All of the reported and proposed improvements in image formation and deconvolution are of value in multiple areas of biological microscopy and clinical imaging.

In conclusion, we report the unique capability of measuring the uniaxial PT of diverse specimens using simple add-on modules on a commercial microscope and an open-source inverse algorithm based on vectorial diffraction theory. PTI has allowed us to image myelination and 3D orientation of axons in mouse brain tissue, the organelle architecture of SARS-CoV-2-infected CMs and RSV-infected A549 cells and 3D anisotropy of the H&E-stained tissues. The comprehensive analysis of architecture enabled by PTI can address open questions of fundamental importance and lead to markers of clinical relevance. Similarly, it can enable quantitative analysis and discovery of new material properties in the material science community.

## Methods

### Imaging model

We need an accurate imaging model that relates the uniaxial PT to measured intensities to develop an inverse algorithm that reconstructs the PT. Our microscope and therefore the model utilizes the concepts of vector wave equation^70,71^, scattering potential of a specimen^72^ and partially coherent imaging^73,74^. We summarize the model and underlying assumptions in this section and provide a detailed derivation in Supplementary Note 1.

To keep the model mathematically tractable, we make two key assumptions in addition to the uniaxial symmetry of molecular distribution discussed before. The first assumption is that the recorded intensity modulations are dominated by the interference of the light scattered only once by a weak-scattering specimen (the first Born approximation^4^ is valid). This assumption allows us to develop an accurate single-scattering vectorial imaging model termed, ‘vector Born model’, which we use for all the forward simulations presented in this paper. Reconstructing components of PT from this model is computationally prohibitive due to the nonlinear relationship between the intensity and the components of PT. The second assumption is that the interference between two singly scattered photons is negligible (the weak-object approximation is valid)^21^. We term this model ‘linearized vector Born model’ and use it to develop our inverse algorithm. The assumptions of weak scattering and weak object are typically valid for ~50-μm thick cells and tissues. These assumptions fundamentally limit the depth of imaging of many single-photon imaging methods.

The linearized vector Born model is a vector diffraction model and consists of several improvements relative to the previously reported models. First, we express the distribution of the scattered electric field vector in terms of the distribution of the Stokes parameters. Stokes parameters are linearly related to recorded intensities and, therefore, can be calibrated accurately (ref. ^27^ and Supplementary Note 2). Second, the model establishes a linear relationship between the 3D Stokes images and the unknown 3D distribution of the uniaxial PT of the specimen, through a set of OTFs parameterized by the size, pattern and polarization of the illumination and detection apertures of the microscope. This multi-channel transfer function model enables the recovery of specimen properties via multi-channel deconvolution.

In the following description, the coordinates $\overrightarrow{r}$ and $\overrightarrow{u}$ represent the 3D spatial and spatial frequency coordinates, respectively, in the object space. The coordinates ${\overrightarrow{r}}_{\perp }$ and ${\overrightarrow{u}}_{\perp }$ represent the 2D spatial and spatial frequency coordinates in the object space. The coordinate ${\overrightarrow{\nu }}_{\perp }$ represents the location of a point source in the illumination pupil, which corresponds to the spatial frequency of the illumination.

#### PT and scattering potential tensor

We measure relative PT of specimens, which is a dimensionless quantity. The relative PT of a uniaxial material oriented with in-plane orientation, *ω* and inclination, *θ*, as shown in Fig. 1b is expressed as

1 $$\begin{array}{l}{\mathop{\epsilon}\limits^{=}}{{}_{{{{\rm{r}}}}}}=\left[\begin{array}{ll}{\epsilon }_{{{{\rm{r}}}}}-\Delta {\epsilon }_{{{{\rm{r}}}}}({\cos }^{2}\theta -{\sin }^{2}\theta \cos 2\omega )&\Delta {\epsilon }_{{{{\rm{r}}}}}{\sin }^{2}\theta \sin 2\omega \\ \Delta {\epsilon }_{{{{\rm{r}}}}}{\sin }^{2}\theta \sin 2\omega &{\epsilon }_{{{{\rm{r}}}}}-\Delta {\epsilon }_{{{{\rm{r}}}}}({\cos }^{2}\theta +{\sin }^{2}\theta \cos 2\omega ) \\ \Delta {\epsilon }_{{{{\rm{r}}}}}\sin 2\theta \cos \omega &\Delta {\epsilon }_{{{{\rm{r}}}}}\sin 2\theta \sin \omega \end{array}\right.\\\qquad\left.\begin{array}{l}\Delta {\epsilon }_{{{{\rm{r}}}}}\sin 2\theta \cos \omega\\\Delta {\epsilon }_{{{{\rm{r}}}}}\sin 2\theta \sin \omega\\{\epsilon }_{{{{\rm{r}}}}}+\Delta {\epsilon }_{{{{\rm{r}}}}}\cos 2\theta\end{array}\right],\end{array}$$

where

2 $$\begin{array}{rcl}{\epsilon }_{{{{\rm{r}}}}}&=&\frac{1}{2}\left({n}_{{{{\rm{e}}}}}^{2}+{n}_{{{{\rm{o}}}}}^{2}\right)\\ \Delta {\epsilon }_{{{{\rm{r}}}}}&=&\frac{1}{2}\left({n}_{{{{\rm{e}}}}}^{2}-{n}_{{{{\rm{o}}}}}^{2}\right),\end{array}$$

*n*_o_ and *n*_e_ are RIs experienced by the ordinary and extraordinary wave, respectively.

Diffraction tomography approaches have relied on the scattering potential^43^ and 2 × 2 scattering potential tensor^28,44^ models to reconstruct volumetric distribution of density and projected anisotropy, respectively. We extend this concept and model 3 × 3 scattering potential tensor to reconstruct volumetric distribution of density, 3D anisotropy and material symmetry. The scattering potential tensor is defined as

3 $$\begin{array}{rcl}&&{\mathop{f}\limits^{=}}={k}_{0}^{2}\left(\mathop{\epsilon }\limits^{=}_{{{{\rm{r}}}}}-{\epsilon }_{{{{\rm{rm}}}}}\right)=\left[\begin{array}{ccc}{f}_{0}+{f}_{1{{{\rm{c}}}}}&{f}_{1{{{\rm{s}}}}}&{f}_{2{{{\rm{c}}}}}\\ {f}_{1{{{\rm{s}}}}}&{f}_{0}-{f}_{1{{{\rm{c}}}}}&{f}_{2{{{\rm{s}}}}}\\ {f}_{2{{{\rm{c}}}}}&{f}_{2{{{\rm{s}}}}}&{f}_{0}+{f}_{3}\end{array}\right],\\ {{{\rm{where}}}}&&\left\{\begin{array}{l}{f}_{0}={k}_{0}^{2}\left({\epsilon }_{{{{\rm{r}}}}}-{\epsilon }_{{{{\rm{rm}}}}}-\Delta {\epsilon }_{{{{\rm{r}}}}}{\cos }^{2}\theta \right)\\ {f}_{1{{{\rm{c}}}}}={k}_{0}^{2}\Delta {\epsilon }_{{{{\rm{r}}}}}{\sin }^{2}\theta \cos 2\omega \\ {f}_{1{{{\rm{s}}}}}={k}_{0}^{2}\Delta {\epsilon }_{{{{\rm{r}}}}}{\sin }^{2}\theta \sin 2\omega \\ {f}_{2{{{\rm{c}}}}}={k}_{0}^{2}\Delta {\epsilon }_{{{{\rm{r}}}}}\sin 2\theta \cos \omega \\ {f}_{2{{{\rm{s}}}}}={k}_{0}^{2}\Delta {\epsilon }_{{{{\rm{r}}}}}\sin 2\theta \sin \omega \\ {f}_{3}={k}_{0}^{2}\Delta {\epsilon }_{{{{\rm{r}}}}}\left(3{\cos }^{2}\theta -1\right)\end{array}\right.,\end{array}$$

*k*_0_ = 2*π*/*λ*_0_ is the free-space wavenumber, *λ*_0_ is the free-space wavelength of the light and *ϵ*_rm_ is the isotropic relative permittivity of the surrounding medium. The scattering potential tensor contains the same information as the PT of the specimen, except that it is relative to the permittivity of the surrounding medium. Note that all the variables in the above equations can be a function of 3D space, $\overrightarrow{r}={[x,y,z]}^{T}$, which are used in the later derivations.

#### Vector Born model

Interaction between the incident light and the scattering potential tensor of the specimen results in the scattered electric field. The field is derived based on the vector wave equation^70,71^ and the first Born approximation^4^ as

4 $$\begin{array}{r}{\overrightarrow{E}}_{{{{\rm{out}}}}}(\overrightarrow{r})\approx {\overrightarrow{E}}_{{{{\rm{inc}}}}}(\overrightarrow{r})+\displaystyle\iiint \mathop{G}\limits^{=}(\overrightarrow{r}-{\overrightarrow{r}}{}^{{\prime} })\mathop{f}\limits^{=}({\overrightarrow{r}}{}^{{\prime}})\,{\overrightarrow{E}}_{{{{\rm{inc}}}}}({\overrightarrow{r}}{}^{{\prime} }){d}^{\,{3}}\overrightarrow{r}.\end{array}$$

where ${\overrightarrow{E}}_{{{{\rm{out}}}}}(\overrightarrow{r})={\left[{E}_{{{{\rm{out}}}},x}(\overrightarrow{r}),{E}_{{{{\rm{out}}}},y}(\overrightarrow{r}),{E}_{{{{\rm{out}}}},z}(\overrightarrow{r})\right]}^{T}$ is the scattered output electric field in 3D space $\overrightarrow{r}={[x,y,z]}^{T},{\overrightarrow{E}}_{{{{\rm{inc}}}}}(\overrightarrow{r})$ is the incident electric field and $\mathop{G}\limits^{=}(\overrightarrow{r})$ is the dyadic Green’s tensor visualized in Fig. 1. This equation describes a single scattering event from a single plane wave incident on the specimen from a specific angle of illumination.

In our experiments, we use partially coherent illumination from large illumination NA to avoid speckle and achieve optical sectioning. In this case, the recorded images are the sum of intensities due to coherent scattering of light at each angle of illumination. Each angle of illumination modulates the specimen with electric field of spatial frequency ${\overrightarrow{\nu }}_{\perp }$. We first define the scattered electric field with incident light of spatial frequency ${\overrightarrow{\nu }}_{\perp }$ to be ${\overrightarrow{E}}_{{{{\rm{out}}}}}(\overrightarrow{r},{\overrightarrow{\nu }}_{\perp })$. The polarization-resolved intensities due to the *α*-th partially coherent illumination patterns are sums of the contribution from individual coherent scattering events. We use a generalized Stokes vector^73,74^ to represent the polarization-resolved images as follows:

5 $$\begin{array}{rcl}&&\left[\begin{array}{c}{S}_{0,\alpha }(\overrightarrow{r})\\ {S}_{1,\alpha }(\overrightarrow{r})\\ {S}_{2,\alpha }(\overrightarrow{r})\\ {S}_{3,\alpha }(\overrightarrow{r})\end{array}\right]=\left[\begin{array}{c}{S}_{xx,\alpha }(\overrightarrow{r})+{S}_{yy,\alpha }(\overrightarrow{r})\\ {S}_{xx,\alpha }(\overrightarrow{r})-{S}_{yy,\alpha }(\overrightarrow{r})\\ {S}_{xy,\alpha }(\overrightarrow{r})+{S}_{yx,\alpha }(\overrightarrow{r})\\ i\left[{S}_{xy,\alpha }(\overrightarrow{r})-{S}_{yx,\alpha }(\overrightarrow{r})\right]\end{array}\right],\\ {{{\rm{where}}}}&&{S}_{pq,\alpha }(\overrightarrow{r})=\displaystyle{\iint }_{{\overrightarrow{\nu }}_{\perp }\in \alpha }{E}_{{{{\rm{out}}}},p}(\overrightarrow{r},{\overrightarrow{\nu }}_{\perp }){E}_{{{{\rm{out}}}},q}^{\,{*}}(\overrightarrow{r},{\overrightarrow{\nu }}_{\perp }){d}^{\,{2}}{\overrightarrow{\nu }}_{\perp }.\end{array}$$

A key advantage of our model is that the Stokes parameters can be directly measured and calibrated as detailed in 2, which allows us to match the model to the microscope, leading to the high-quality reconstructions that we have reported in the paper.

We have now summarized a rigorous single-scattering vectorial imaging model termed, ‘vector Born model’, which we use for all the forward simulations presented in this paper. The Stokes images in the vector Born model have quadratic dependence on the scattering potential tensor.

#### Linearized vector Born model

Recovery of the scattering potential tensor using a rigorous vector Born model requires an iterative, computationally expensive, inverse algorithm. To make the inverse algorithm computationally tractable, we developed a linearized vector Born model. We neglected the nonlinear contribution to the Stokes parameters from the scattering potential tensor, which is usually small for weakly scattering specimens (weak-object approximation^21^), which led to the linearized vector Born model, expressed in the Fourier space:

6 $${\tilde{S}}{}_{m,\alpha}^{{\prime}}(\overrightarrow{u})=\mathop{\sum}\limits_{{\ell=0{{{\rm{r}}}},0{{{\rm{i}}}},1{{{\rm{c}}}},}\atop{1{{{\rm{s}}}},2{{{\rm{c}}}},2{{{\rm{s}}}},3}}{\tilde{H}}_{m,\ell ,\alpha }(\overrightarrow{u})\,{\tilde{f}}_{\ell }(\overrightarrow{u});\quad m=0,1,2,3,$$

where ${\tilde{a}}({\overrightarrow{u}})$ denotes the Fourier transform of a function $a({\overrightarrow{r}})$ at the 3D spatial frequency, $\overrightarrow{u}={[{{\overrightarrow{u}}_{\perp}}{}^{T},{u}_{z}]}^{T},{\tilde{S}}_{m,\alpha}^{{\prime} }(\overrightarrow{u})$ is the DC-subtracted Stokes parameter and ${\tilde{H}}_{m,\ell ,\alpha }({\overrightarrow{u}})$ is the transfer function that maps each scattering potential tensor component *ℓ* to the *m*-th Stokes parameter under illumination pattern *α*.

When the depth of field of the imaging system is larger than the thickness of the specimen, the specimen-scattering potential is a 2D function $\mathop{f}\limits^{=}({\overrightarrow{r}}_{\perp })$ whose Fourier transform is filtered by the 2D transfer functions $\int {\tilde{H}}_{m,\ell ,\alpha }(\overrightarrow{u})d{u}_{z},$ resulting in the following model:

7 $$\begin{array}{rcl}&&{\tilde{S}}_{m,\alpha }^{{\prime} (z = 0)}({\overrightarrow{u}}_{\perp })=\mathop{\sum }\limits_{{\ell = 0{{{\rm{r}}}},0{{{\rm{i}}}},1{{{\rm{c}}}},}\atop {1{{{\rm{s}}}},2{{{\rm{c}}}},2{{{\rm{s}}}},3}}\left[\displaystyle\int{\tilde{H}}_{m,\ell ,\alpha }(\overrightarrow{u})d{u}_{z}\right]{\tilde{f}}_{\ell }({\overrightarrow{u}}_{\perp });\quad m=0,1,2,3,\end{array}$$

### Inverse algorithm and image analysis

Our inverse algorithm takes the Stokes parameters of the scattered light under different illuminations as inputs and reconstructs the physical properties encoded by the PT. The inverse algorithm is structured into three modules to achieve robust estimation and computational efficiency. The first part of the algorithm is a least-square optimization solver that estimates the components of the scattering potential tensor in 3D space. Second, we compute the mean permittivity, differential permittivity and 3D orientation from the entries of the scattering potential tensor assuming that each voxel is a positive and a negative uniaxial material. The last part of the algorithm fits these two solutions to the recorded Stokes volumes via the linearized vector Born model to estimate the optic sign in 3D. We derive the inverse algorithm in Supplementary Note 3. We describe image analysis algorithms for multi-scale analysis, denoising and comparing structure tensor and PT in Supplementary Note 4. The current open-source implementation of the inverse algorithm and image analysis algorithms is maintained on GitHub at waveorder.

### Multi-scale imaging and analysis

We automate the multi-scale imaging shown in Fig. 4 and Supplementary Video 4 by controlling individual devices in Python. This acquisition requires control of three main devices, the LCD panel for switching illumination patterns, the machine-vision polarization camera for collecting images and the microscope stages for scanning in *x*, *y* and *z* directions. First, we control the LCD panel using the built-in APIs from Adafruit with Arduino board. Serial connection is established from the acquisition computer to the Arduino board for software triggering in Python. Second, we control the polarization camera with a Python package, PySpin, developed by camera manufacturer, FLIR. Last, the microscope stage is controlled by Micro-Manager (https://github.com/micro-manager). To build a bridge between the Java-based Micro-Manager and Python, we leverage the mm2python library (https://github.com/czbiohub-sf/mm2python). Collectively, these packages allow us to compose an acquisition script to control each device. For the 2D acquisition shown in Fig. 4a,b, we acquired nine images under different illumination patterns per location for a total of 609 FOVs in a 29 × 21 (*x* × *y*) rectangular grid. The overlap between each location is set to be ~15% in the *x* direction and ~30% in the *y* direction. For the 3D acquisition shown in Fig. 4c,i and Supplementary Video 4, we acquired 9 × 120 (pattern × *z*) images to form a *z*-stack per location for a total of 153 FOVs in a 17 × 9 (*x* × *y*) rectangular grid. The overlap parameter is similar to the previous case.

We implemented our algorithm to be GPU compatible on an IBM Power9 server equipped with four GPUs (Tesla V100-SXM2-32GB, NVIDIA) per compute node. One FOV of 2D acquisition (~1,200 × 1,000 pixels) easily fits in the memory of one GPU, so we initiate four instances of computation with four different GPU to process the data. The algorithm takes about 200 s to process one FOV of 2D acquisition and about half an hour to stitch 609 FOVs for one deconvolved channel. Fitting a FOV of 3D acquisition in the memory of one GPU is infeasible, so we broke one 3D acquisition into 30 (or smaller number) small patches for processing. We also initiate four to eight instances of computation with four GPUs to process these small patches in parallel. Each FOV of the 3D acquisition (~1,200 × 1,000 × 120 voxels) takes about 6 h of the processing time and the stitching process of all 153 volumes takes about 3 h for one channel of the reconstruction.

### Specimen preparation

#### Femtosecond laser-written anisotropic glass

The target used in Fig. 3 was written into a fused silica cover glass that was about 0.25-mm thick and 22 × 22 mm on its sides using a polarized femtosecond laser. The star pattern consists of 32 equally spaced birefringent wedges that rotate in steps of 11.25°. The wedges consist of a single line near the center of the star, flanked by additional lines toward larger diameters (one line between 3 and 20 μm in diameter, three lines between 20 and 40 μm and five lines between 40 and 60 μm). While the slow axis of a wedge rotated with the wedge, within a wedge, the slow axis was uniform and was parallel to the lines. Each line was written only once and the scanning direction was parallel to the slow axis. The parameters of laser fabrication were the following: pulse duration of 500 fs, repetition rate of 500 kHz, fabrication speed of 0.01 mm s^−1^, wavelength of 515 nm and focused with a 0.55-NA lens. More details on the fused silica modification through laser writing are documented in Supplementary Note 6.

#### Mouse brain section

The mice were anesthetized by inhalation of isoflurane in a chemical fume hood and then perfused with 25 ml phosphate-buffered saline (PBS) into the left cardiac ventricle and subsequently with 25 ml 4% paraformaldehyde (PFA) in the PBS solution. Thereafter, the brains were post-fixed with 4% PFA for 12–16 h and then transferred to 30% sucrose solution at the temperature of 4 °C for 2–3 days until the tissue sank to the bottom of the container. Then, the brains were embedded in a tissue freezing medium (Tissue-Tek OCT compound 4583, Sakura) and kept at −80 °C. Cryostat-microtome (Leica CM 1850) was used for preparing the tissue sections (12 and 50 μm) at −20 °C and the slides were stored at −20 °C until use. Upon experiment, the OCT on the slides was melted by keeping the slides at 37 °C for 15–30 min. Then, the slides were washed in PBS-T (PBS + Tween-20 (0.1%)) for 5 min and then washed in PBS for 5 min and coversliped by mounting medium (F4680, Fluoromount Aqueous, Sigma).

#### iPS cell CMs

CMs were differentiated from iPS cells (WTc cell line^75^) using a modified Wnt pathway modulation protocol^76^. In brief, cells were maintained in mTesr medium (Stem Cell Technologies) and 3 days before differentiation, they were seeded on 12-well plates. During differentiation, basal medium was RPMI supplemented with B-27 minus insulin (Gibco) for days 0–7 and RPMI with B-27 (Gibco) on days 7 onwards. Cells were treated with 6 μM CHIR99021 (Tocris) for 48 h on day 0 and with 5 μM IWP2 (Tocris) for 48 h on day 3. On day 15, cells were collected and stored on cryovials. When ready for experiments, cell pools were thawed in RPMI with B-27 supplemented with 20% FBS (HyClone) and ROCK inhibitor Y-27632 (10 μM, Selleckchem). CMs were then selected in culture using a metabolic switch method^77^ by treating the cells with 4 mM lactate medium changes every other day for 6 days. Final cultures were >90% ACTN+.

On day 30, CMs were replated into glass coverslips and maintained on RPMI with B-27 for five more days. Then they were fixed using 4% paraformaldehyde for 20 min at room temperature, washed three times with PBS supplemented with Triton X-100 (PBS-T) and blocked and permeabilized with 5% bovine serum albumin in PBS-T. Cells were then stained with an anti-cTnT antibody (Abcam, ab45932, dilution 1:400) in PBS-T overnight and with DAPI for 10 min. After three PBS-T washes, Alexa Fluor 488 anti-rabbit (Thermo Fisher, A-21202, dilution 1:400) in PBS-T was used as secondary antibody, followed by three more PBS-T washes. Then, a drop of Prolong Antifade (without DAPI) (Thermo Fisher) was added to coverslips for mounting into a glass slide.

#### RSV infection in A549 cells

A549 cells were maintained in Dulbecco’s modified Eagle medium (DMEM) and supplemented with 10% FBS and 1% pen/strep and cells were seeded to 70% confluency in ibidi eight-well glass bottom chamber slides (cat. no. 80841). At 24 h after seeding, the cells were inoculated with RSV at 0.1 multiplicity of infection in serum-free DMEM for 90 min at 37 °C. The inoculating medium was replaced with DMEM supplemented with 10% FBS and 1% pen/strep and returned to the 37 °C incubator. At 24 h and 48 h post-infection, the cells were fixed with 4% paraformaldehyde in PBS for 15 min, washed with PBS and stained with 1 μM DAPI. Glass coverslips were mounted to the slides with Invitrogen Prolong Diamond Antifade Mountant (Thermo Fisher Scientific, cat. no. P36961).

### Statistics and reproducibility

The experiment was repeated once (Figs. 3b and 4a–c and Extended Data Fig. 7), twice (Figs. 2, 3a,c, 5 and 6, Extended Data Fig. 10 and Supplementary Figs. 2 and 5), three times (Fig. 1c,d), seven times (Fig. 4d–g and Supplementary Fig. 4) and eight times (Extended Data Fig. 5).

## Availability of biological materials

All unique biological materials classified at Biosafety Level 1 and 2 are available from authors upon reasonable request or from commercial sources. Mouse brain tissue sections can be requested from the Han laboratory (Stanford University), iPS cell-derived CMs can be requested from the Conklin laboratory (Gladstone Institutes), the A549 cell line is available from ATCC and the RSV–GFP virus is available from ViraTree. SARS-CoV-2 infection experiments were conducted in a Biosafety Level 3 laboratory of Gladstone Institutes and are not accessible readily.

### Reporting summary

Further information on research design is available in the Nature Portfolio Reporting Summary linked to this article.

## Online content

Any methods, additional references, Nature Portfolio reporting summaries, source data, extended data, supplementary information, acknowledgements, peer review information; details of author contributions and competing interests; and statements of data and code availability are available at 10.1038/s41592-024-02291-w.

### Supplementary information

Supplementary Information Supplementary Figs. 1–7, Supplementary Notes 1–7, references and captions for Videos 1–13.

Reporting Summary

Supplementary Video 1 The build video demonstrating how to set up PTI on a working microscope.

Supplementary Video 2 z-stacks of 3D mean permittivity, 3D differential permittivity, 3D orientation (rendered separately in in-plane orientation and out-of-plane tilt) and optic sign probability images of the laser-written anisotropic target shown in Fig. 3.

Supplementary Video 3 z-stacks of 3D mean permittivity, 3D differential permittivity, 3D orientation (rendered separately in in-plane orientation and out-of-plane tilt) and optic sign probability images of the laser-written anisotropic target shown in Extended Data Fig. 5.

Supplementary Video 4 Visualization of high-resolution (1.47 NA) 3D mean permittivity and 3D differential permittivity of the adult mouse brain section.

Supplementary Video 5 z-stacks, *x*–*z* section and *y*–*z* section of 3D mean permittivity, 3D differential permittivity, 3D orientation (rendered separately in in-plane orientation and out-of-plane tilt) and optic sign probability images of FOV (1) of the mouse brain section shown in Fig. 4.

Supplementary Video 6 z-stacks of 3D mean permittivity, 3D differential permittivity, 3D orientation (rendered separately in in-plane orientation and out-of-plane tilt), optic sign probability and fluorescence images of fixed uninfected CMs shown in Fig. 5a.

Supplementary Video 7 z-stacks of 3D mean permittivity, 3D differential permittivity, 3D orientation (rendered separately in in-plane orientation and out-of-plane tilt), optic sign probability and fluorescence images of SARS-CoV-2-infected CMs shown in Fig. 5d (left).

Supplementary Video 8 z-stacks of 3D mean permittivity, 3D differential permittivity, 3D orientation (rendered separately in in-plane orientation and out-of-plane tilt), optic sign probability and fluorescence images of SARS-CoV-2-infected CMs shown in Fig. 5d (right).

Supplementary Video 9 z-stacks of 3D mean permittivity, 3D differential permittivity, 3D orientation (rendered separately in in-plane orientation and out-of-plane tilt), optic sign probability and H&E images of the mammal cardiac tissue shown in Fig. 6a (top).

Supplementary Video 10 z-stacks of 3D mean permittivity, 3D differential permittivity, 3D orientation (rendered separately in in-plane orientation and out-of-plane tilt), optic sign probability and H&E images of the human uterus tissue shown in Fig. 6a (bottom).

Supplementary Video 11 z-stacks of 3D mean permittivity, 3D differential permittivity, 3D orientation (rendered separately in in-plane orientation and out-of-plane tilt), optic sign probability and fluorescence images of fixed uninfected A549 cells shown in Fig. 2.

Supplementary Video 12 z-stacks of 3D mean permittivity, 3D differential permittivity, 3D orientation (rendered separately in in-plane orientation and out-of-plane tilt), optic sign probability and fluorescence images of RSV-infected A549 cells shown in Fig. 3.

Supplementary Video 13 3D animation illustrating the key concepts underlying permittivity tensor imaging: (1) The architecture of a cylindrical lipidbilayer is represented as a positively uniaxial permittivity tensor distribution at high-resolution, but as a negatively uniaxialpermittivity tensor at coarse resolution. (2) The permittivity tensor of an optical section in the brain tissue are encoded inimages. Notice that the structure affects the images, but cannot be interpreted from the images. The field of view shown hereis the same as in fig. 1. (3) The image formation model that maps the isotropic material or anisotropic material to images isillustrated. (4) The inverse algorithm recovers the components of the uniaxial PT from the high-dimensional image data. Thefield of view shown here is the same as in fig. 1. (5) Measurements of the permittivity tensors at the scales ranging from thesingle axon to the corpus callosum are illustrated.

### Source data

Source Data Table Table of data sources for figures and extended figures. Each tab has a table of source data files, their sizes and direct download links to the source data. Figures 3–6 and Extended Figs. 5, 6 and 10 have linked data.

## Data Availability

Experimental data reported in this manuscript are available at the Bioimage Archive (https://www.ebi.ac.uk/biostudies/bioimages/studies/S-BIAD1063). This includes raw data and processed data for Figs. 3a, 4d–f, 5 and 6, Extended Data Fig. 5 and Supplementary Fig. 3. Simulated PTI images and reconstructions (Extended Data Fig. 2) are available as examples documented in our repository (https://github.com/mehta-lab/waveorder). The Allen brain reference atlas (https://mouse.brain-map.org/static/atlas) was used to register the anatomical landmarks of the mouse brain section in Fig. 4a–c. Source data are provided with this paper.

## Code availability

The source code for simulations and reconstructions can be found at waveorder (https://github.com/mehta-lab/waveorder). Release 2.1.0 provides an accessible demonstration of the image formation and inverse algorithms. For the Micro-Manager Python bridge, we used mm2python (https://github.com/czbiohub-sf/mm2python) for the first prototype and then used pycro-manager (https://github.com/micro-manager/pycro-manager) for the automated acquisition.
