# Supplementary material for: Permittivity tensor imaging: modular label-free imaging of 3D dry mass and 3D orientation at high resolution
Source: Nat Methods. 2024 Jun 18;21(7):1257–74. doi: 10.1038/s41592-024-02291-w (PMC11239526; doi:10.1038/s41592-024-02291-w)
Supplement: Supplementary file 1 — Supplementary Figs. 1–7, Supplementary Notes 1–7, references and captions for Videos 1–13. [file 41592_2024_2291_MOESM1_ESM.pdf]

nature methods

Supplementary information

https://doi.org/10.1038/s41592-024-02291-w

Permittivity tensor imaging: modular label-free imaging of 3D dry mass and 3D orientation at high resolution

In the format provided by the authors and unedited

1. DERIVATION OF THE IMAGING MODEL

In this note, we derive the imaging model that expresses the measured intensities in terms of the permittivity tensor of the specimen and the configuration of the microscope. Our model accounts for the following:

- Scattering of the vector electric field by the permittivity tensor [1, 2] of the specimen.
- Illuminating the specimen with incoherent source and finite illumination aperture, i.e., partially coherent illumination [3, 4, 5, 6].
- Polarization of electric field along the imaging axis due to high-NA illumination and imaging.

We assume that the imaging path is free of scalar or vector aberrations. We also assume that the specimens we image are weak scatterers and multiple scattering is negligible.

Some notes on the nomenclature:

- We track the dimensions of physical quantities through the development of the imaging model to clarify the properties of the specimen that we recover from the measured intensities.
- In optics and the following derivation, the term polarization can imply two distinct quantities: polarization of material and polarization of light. The former refers to the induction of electric dipoles in a material as it interacts with the light, and the latter refers to the orientation of the electric field of light. Unless explicitly stated, the word polarization refers to the polarization of light.

1.1. **Permittivity tensor.**

Permittivity is a measure of how much a material is polarized by an incident electric field. The more easily a material is polarized, the more it delays the electromagnetic wave traveling through it, and the higher the permittivity of the material. Polarization of the material induces electric dipoles, which radiate scattered field. Permittivity of an isotropic material such as water is a scalar, i.e., the induced electric dipole moment aligns and scales linearly with the applied electric field. The polarization of an anisotropic material depends on the angle between the electric field and the symmetry axis of the material. As a result, the strength and the orientation of the induced dipole moments may not align with the direction of the incident electric field. The permittivity of an anisotropic material hence takes a form of a second-order tensor [7, 8].

At the spatial scale of visible wavelengths, a material can be considered to be isotropic, uniaxial, or biaxial depending on the symmetry of the molecular distribution [7]. Here we focus on biological structures that are uniaxial, i.e., materials that have one anisotropic axis. The symmetry axes of the material are called optic axes.

If a uniaxial material's symmetry axis is aligned with the z-axis, its relative permittivity tensor is a diagonal matrix:

$$\bar{\bar{\varepsilon}}_{\mathrm{r}} = \begin{bmatrix} n_{\mathrm{o}}^2 & 0 & 0 \\ 0 & n_{\mathrm{o}}^2 & 0 \\ 0 & 0 & n_{\mathrm{e}}^2 \end{bmatrix}, \tag{1}$$

where n_{o} and n_{e} are refractive indices experienced by the ordinary and extraordinary wave, respectively. Refractive indices are generally complex numbers, whose real part represents phase delay and imaginary part represents absorption.

Permittivity tensors can be parameterized in several ways: with independent entries of the matrix representation, with eigenvalues and eigenvectors of the matrix representation, or with parameters of the angular distribution of biomolecules. To facilitate physical interpretation of the material properties, we parameterize the permittivity tensor in terms of angular distribution of biomolecules using mean (ε_{r}) and difference ($\Delta\varepsilon_{\mathrm{r}}$) of the ordinary and extraordinary permittivity. They are related to the refractive indices as follows:

$$\begin{aligned} \varepsilon_{\mathrm{r}} &= \frac{1}{2}\left(n_{\mathrm{e}}^2 + n_{\mathrm{o}}^2\right) \\ \Delta\varepsilon_{\mathrm{r}} &= \frac{1}{2}\left(n_{\mathrm{e}}^2 - n_{\mathrm{o}}^2\right) \end{aligned} \tag{2}$$

Permittivity tensors have the same dimensions as the scalar permittivity of vacuum, ε_0. Microscopes are sensitive only to the relative permittivity tensor, which is the ratio of the material's permittivity to the vacuum's permittivity. The relative permittivity and the refractive indices in (1) and (2) are dimensionless quantities.

In terms of the mean and differential permittivities, the permittivity tensor in (1) is expressed as

$$\overline{\overline{\varepsilon}}_{\mathrm{r}} = \begin{bmatrix} \varepsilon_{\mathrm{r}} - \Delta\varepsilon_{\mathrm{r}} & 0 & 0 \\ 0 & \varepsilon_{\mathrm{r}} - \Delta\varepsilon_{\mathrm{r}} & 0 \\ 0 & 0 & \varepsilon_{\mathrm{r}} + \Delta\varepsilon_{\mathrm{r}} \end{bmatrix} \tag{3}$$

A uniaxial material can have arbitrary 3D orientation. We represent the 3D orientation of the permittivity tensor with in-plane orientation, ω, and inclination, θ, as shown in fig. 1B. The permittivity tensor in the microscope's frame of reference is computed by applying two rotational transformations [9] to $\overline{\overline{\varepsilon}}_{\mathrm{r}}$ and is expressed as

$$\begin{aligned} \overline{\overline{\varepsilon}}_{\mathrm{r}}(\omega,\theta) &= \overline{\overline{T}}(\omega,\theta)^{-1} \cdot \overline{\overline{\varepsilon}}_{\mathrm{r}} \cdot \overline{\overline{T}}(\omega,\theta) \\ &= \begin{bmatrix} \varepsilon_{\mathrm{r}} - \Delta\varepsilon_{\mathrm{r}}(\cos^2\theta - \sin^2\theta\cos 2\omega) & \Delta\varepsilon_{\mathrm{r}}\sin^2\theta\sin 2\omega & \Delta\varepsilon_{\mathrm{r}}\sin 2\theta\cos\omega \\ \Delta\varepsilon_{\mathrm{r}}\sin^2\theta\sin 2\omega & \varepsilon_{\mathrm{r}} - \Delta\varepsilon_{\mathrm{r}}(\cos^2\theta + \sin^2\theta\cos 2\omega) & \Delta\varepsilon_{\mathrm{r}}\sin 2\theta\sin\omega \\ \Delta\varepsilon_{\mathrm{r}}\sin 2\theta\cos\omega & \Delta\varepsilon_{\mathrm{r}}\sin 2\theta\sin\omega & \varepsilon_{\mathrm{r}} + \Delta\varepsilon_{\mathrm{r}}\cos 2\theta \end{bmatrix} \end{aligned} \tag{4}$$

where $\overline{\overline{T}}(\omega,\theta)$ is the overall coordinate rotation matrix that is composed of two consecutive rotations around z and y axes as

$$\overline{\overline{T}}(\omega,\theta) = \begin{bmatrix} \cos\theta & 0 & -\sin\theta \\ 0 & 1 & 0 \\ \sin\theta & 0 & \cos\theta \end{bmatrix} \cdot \begin{bmatrix} \cos\omega & \sin\omega & 0 \\ -\sin\omega & \cos\omega & 0 \\ 0 & 0 & 1 \end{bmatrix} \tag{5}$$

Note that all the variables in the above equations can be a function of 3D space, $\vec{r} = [x,y,z]^T$.

1.2. **Vector wave equation and scattering potential tensor.** We will now discuss how the permittivity tensor is encoded by the scattered electric field. The scalar diffraction theory [7] has enabled development of the optical diffraction tomography methods that measure complex refractive index (RI). The key insight that enables optical diffraction tomography is that the diffracted field is a 3D Fourier transform of the scattering potential of the material [10].

We extend the scalar scattering potential formalism to *scattering potential tensors*.

We start by considering the vector wave equation derived from Maxwell's equations as per [1, 2]

$$\nabla \times \nabla \times \vec{E}(\vec{r}) = k_0^2 \overline{\overline{\varepsilon}}_{\mathrm{r}}(\vec{r})\vec{E}(\vec{r}), \tag{6}$$

where $\vec{E}(\vec{r}) = [E_x(\vec{r}), E_y(\vec{r}), E_z(\vec{r})]^T$ is the electric field in 3D space $\vec{r} = [x,y,z]^T$. The dimensions of x, y, and z are $[L]$, where L means length. $k_0 = 2\pi/\lambda_0$ is the free-space wavenumber with the dimension of $[L^{-1}]$, and λ_0 is the free-space wavelength of the light with the dimension of $[L]$. The dimensions of the ∇ operator are $[L^{-1}]$.

We now consider that the material is surrounded by a medium with scalar permittivity, ε. The vector wave equation in the presence of the surrounding medium is given by

$$\nabla \times \nabla \times \vec{E}(\vec{r}) - k_0^2 \varepsilon_{\mathrm{rm}} \vec{E} = k_0^2 \left[\overline{\overline{\varepsilon}}_{\mathrm{r}}(\vec{r}) - \varepsilon_{\mathrm{rm}}\right] \vec{E}(\vec{r}) = \overline{\overline{f}}(\vec{r})\vec{E}(\vec{r}), \tag{7}$$

where $\varepsilon_{\mathrm{rm}}$ is the relative permittivity of the surrounding media and is a dimensionless scalar. $\overline{\overline{f}}(\vec{r}) = k_0^2 \left[\overline{\overline{\varepsilon}}_{\mathrm{r}}(\vec{r}) - \varepsilon_{\mathrm{rm}}\right]$ is the scattering potential tensor with the dimension of $[L^{-2}]$ whose components are:

$$\overline{\overline{f}}(\vec{r}) = \begin{bmatrix} f_{xx}(\vec{r}) & f_{xy}(\vec{r}) & f_{xz}(\vec{r}) \\ f_{yx}(\vec{r}) & f_{yy}(\vec{r}) & f_{yz}(\vec{r}) \\ f_{zx}(\vec{r}) & f_{zy}(\vec{r}) & f_{zz}(\vec{r}) \end{bmatrix}, \tag{8}$$

When the field propagates in homogeneous media, i.e., $\overline{\overline{\varepsilon}}_{\mathrm{r}} = \varepsilon_{\mathrm{rm}}$, (7) reduces to the homogeneous wave equation $\nabla \times \nabla \times \vec{E}_{\mathrm{inc}}(\vec{r}) = 0$, whose solution is the incident propagating wave $\vec{E}_{\mathrm{inc}}$.

The total field in the presence of the specimen is the sum of the incident field and the scattered field ($\vec{E}_{\text{scat}}$), which in turn depends on the total field. The total field is expressed as an inhomogeneous vector wave equation:

$$\begin{aligned}\vec{E}(\vec{r}) &= \vec{E}_{\text{inc}}(\vec{r}) + \vec{E}_{\text{scat}}(\vec{r}) \\ &= \vec{E}_{\text{inc}}(\vec{r}) + \iiint \overline{\overline{G}}(\vec{r}-\vec{r}')\overline{\overline{f}}(\vec{r}')\vec{E}(\vec{r}')d^3\vec{r} \\ &= \vec{E}_{\text{inc}}(\vec{r}) + \overline{\overline{G}}(\vec{r}) \otimes \left[\overline{\overline{f}}(\vec{r})\vec{E}(\vec{r})\right]\end{aligned} \tag{9}$$

$\overline{\overline{f}}(\vec{r})\vec{E}(\vec{r})$ represents the dipoles induced in the specimen by the total electric field. The $\overline{\overline{G}}(\vec{r})$ is the Green's tensor that describes the radiation pattern of an anisotropic point scatterer as derived in [11, 2]. Notice that the scattered field is the 3D convolution of the Green's tensor and the induced dipole distribution.

The Green's tensor is related to the scalar Green's function $G(\vec{r})$ [12, 13] that describes radiation due to an isotropic point scatterer.

$$G(\vec{r}) = \frac{e^{i\sqrt{\varepsilon_{\text{r}}}k_0 r}}{4\pi r}, \quad r = \|\vec{r}\|_2 \tag{10}$$

The scalar Green's function is a spherical wave. Typical imaging systems, however, collect only the forward-propagating component [12].

The component G_{pq} of the Green's tensor describes the electric field along the $p = x,y,z$ axes due to a dipole oriented along $q = x,y,z$ axes [11]. These components are expressed [2, Ch. 10] as follows:

$$\begin{aligned}\overline{\overline{G}}(\vec{r}) &= \begin{bmatrix} G_{xx}(\vec{r}) & G_{xy}(\vec{r}) & G_{xz}(\vec{r}) \\ G_{yx}(\vec{r}) & G_{yy}(\vec{r}) & G_{yz}(\vec{r}) \\ G_{zx}(\vec{r}) & G_{zy}(\vec{r}) & G_{zz}(\vec{r}) \end{bmatrix}, \\ \text{where} \quad G_{pq}(\vec{r}) &= \left(\overline{\overline{I}} + \frac{\partial_p \partial_q}{\varepsilon_{\text{r}} k_0^2}\right) G(\vec{r}), \quad p,q = x,y,z\end{aligned} \tag{11}$$

The dimensions of $G(\vec{r})$ and components of $\overline{\overline{G}}(\vec{r})$ are $[L^{-1}]$. In the integral of (9), the dimension of $\overline{\overline{G}}(\vec{r})\overline{\overline{f}}(\vec{r})$ is $[L^{-3}]$, which results in the dimensionless quantity when integrated over the volume.

1.3. **Born approximation.** The inhomogeneous vector wave equation (9) shows that the interaction between the scattering potential tensor ($\overline{\overline{f}}(\vec{r})$) and the total electric field ($\vec{E}(\vec{r})$) results in the scattered field. In turn, the total field is the sum of the incident and the scattered fields. Thus, there is a recursive functional dependence between the total field and the scattered field. Recursive algorithms [14, 15] have been developed to compute the total and scattered fields, but they are computationally expensive. The computation is especially prohibitive when the forward model is used to solve the 3D inverse problem that we have set out to solve.

Here we consider biological specimens that are relatively weakly scattering. The dominant variations in the total field arise from the incident field and the field that has scattered only once. Hence, we can replace $\vec{E}(\vec{r})$ with $\vec{E}_{\text{inc}}(\vec{r})$ in (9) by assuming the first Born approximation [7] and write the total field as

$$\vec{E}(\vec{r}) \approx \vec{E}_{\text{inc}}(\vec{r}) + \overline{\overline{G}}(\vec{r}) \otimes \left[\overline{\overline{f}}(\vec{r})\vec{E}_{\text{inc}}(\vec{r})\right] \tag{12}$$

This equation describes how the material interacts with the incident electric field under single scattering conditions.

1.4. **3D vectorial partially coherent imaging model.** In this section, we derive the relationship between the vector field in the image plane and the permittivity tensor when the specimen is illuminated with a partially coherent source. We express the field in the image plane in terms of directly measurable Stokes parameters [3, 4]. The relationship between the measurable Stokes parameters and the permittivity tensor is nonlinear in general. To develop a computationally efficient inverse algorithm for reconstructing the 3D permittivity tensor, we sacrifice some accuracy by

linearizing the model with the approach similar to [16, 17, 18, 19, 20]. We will show that this linearization leads to a bank of transfer functions that map the components of the scattering potential tensor to the Stokes parameters. We derive 3D transfer functions that describe imaging of 3D specimens that are thicker than the depth of field. At the end, we reduce them to 2D transfer functions that describe imaging of the specimens that are thinner than the depth of field.

1.4.1. *Electric field of an incident plane wave.* Our polarization microscope measures Stokes parameters of scattered light from the specimen illuminated by a quasi-monochromatic light. Köhler illumination [7] with asymmetric source aperture results in a spatially partially coherent illumination. Each point of the source plane (aperture plane of a illumination condenser lens) generates a plane wave with 3D spatial frequency of $\vec{\nu} = [\nu_x, \nu_y, \nu_z]^T$. $\vec{\nu}_\perp = [\nu_x, \nu_y]^T$ is the transverse spatial frequency, which is proportional to the location of the source point off the imaging axis. ν_z is the axial spatial frequency that satisfies the constraint $|\vec{\nu}_\perp|^2 + \nu_z^2 = \frac{\varepsilon_{\mathrm{rm}}}{\lambda_0^2} = \frac{1}{\lambda^2}$. λ is the wavelength of light inside a media with permittivity of $\varepsilon_{\mathrm{rm}}$. The dimensions of ν_x, ν_y, and ν_z are $[L^{-1}]$.

The incident plane wave due to a point of the source is

$$\vec{E}_{\mathrm{inc}}(\vec{r}, \vec{\nu}_\perp) = \vec{E}_0(\vec{\nu}_\perp) e^{i2\pi\vec{\nu}\cdot\vec{r}}, \tag{13}$$

where $\vec{E}_0(\vec{\nu}_\perp) = [E_{0x}(\vec{\nu}_\perp), E_{0y}(\vec{\nu}_\perp), E_{0z}(\vec{\nu}_\perp)]^T$ are the components of the vector electric field. Notice that we have accounted for the axially (z) polarized component that arises due to high-NA illumination.

The electric field at the aperture of the condenser is typically generated with low-NA optics and does not have a component along the z-axis. The electric field at the source plane is: $\vec{E}_s(\vec{\nu}_\perp) = [E_{sx}(\vec{\nu}_\perp), E_{sy}(\vec{\nu}_\perp), 0)]$. We use high aperture illumination to achieve high 3D resolution, which introduces a z-component in the electric field. The components of the incident electric field are [21]:

$$\begin{aligned}
E_{0x}(\vec{\nu}_\perp) &= \frac{1}{\|\vec{\nu}_\perp\|_2^2}\left[E_{sx}(\vec{\nu}_\perp)\left(\nu_x^2\sqrt{1-\lambda^2\|\vec{\nu}_\perp\|_2^2}+\nu_y^2\right)+E_{sy}(\vec{\nu}_\perp)\nu_x\nu_y\left(\sqrt{1-\lambda^2\|\vec{\nu}_\perp\|_2^2}-1\right)\right] \\
E_{0y}(\vec{\nu}_\perp) &= \frac{1}{\|\vec{\nu}_\perp\|_2^2}\left[E_{sx}(\vec{\nu}_\perp)\nu_x\nu_y\left(\sqrt{1-\lambda^2\|\vec{\nu}_\perp\|_2^2}-1\right)+E_{sy}(\vec{\nu}_\perp)\left(\nu_y^2\sqrt{1-\lambda^2\|\vec{\nu}_\perp\|_2^2}+\nu_x^2\right)\right] \\
E_{0z}(\vec{\nu}_\perp) &= -\left[E_{sx}(\vec{\nu}_\perp)\lambda\nu_x + E_{sy}(\vec{\nu}_\perp)\lambda\nu_y\right]
\end{aligned} \tag{14}$$

1.4.2. *Scattered vector field when the specimen is illuminated by a point.* To simplify the math, we assume the imaging system has magnification of 1. Hence, the spatial coordinate $\vec{r}$ represents the coordinate in both the specimen space and the image space.

In a Köhler geometry, each point in the source plane ($\vec{\nu}_\perp$) generates a coherent plane wave that interacts with the specimen and generates scattered field through (12). The camera in the image plane samples a slice of the 3D field that is blurred by the imaging system, i.e., the 3D field is captured by scanning the sample along z axis. The blur due to the imaging system is expressed as a convolution with a 3D coherent point spread function (PSF) $h_c(\vec{r})$ of the imaging path, which accounts for the finite aperture of the imaging system and the defocus.

We can now combine the effects of the illumination, scattering, and imaging as follows.

- Compute the incident field due to the source point of choice in the specimen volume using (14).
- Compute the total field in the specimen volume by setting $\vec{E}_{\mathrm{inc}} = \vec{E}_0$ in (12) and computing the scattered field as a convolution of the Green's tensor and the induced dipole distribution.
- Filter each component of the specimen field with the 3D coherent PSF to account for the blurring by the microscope.

The operations above lead to the following expression for the vector electric field in the image space due to a point at $\vec{\nu}_\perp$ in the source plane:

$$E_{\mathrm{out},p}(\vec{r}, \vec{\nu}_\perp) = \left\{E_{0p}(\vec{\nu}_\perp)e^{i2\pi\vec{\nu}\cdot\vec{r}} + \sum_{q=x,y,z} G_{pq}(\vec{r}) \otimes \left[\left(\sum_{\ell=x,y,z} f_{q\ell}(\vec{r}) E_{0\ell}(\vec{\nu}_\perp)\right) e^{i2\pi\vec{\nu}\cdot\vec{r}}\right]\right\} \otimes h_c(\vec{r}), \tag{15}$$

where $p = x, y, z$, $\otimes$ denotes 3D convolution. Similar to (9) when the product of the Green's tensor (dimensions $[L^{-1}]$) and the scattering potential tensor (dimensions $[L^{-2}]$) are integrated over the volume, the overall integral of the scattered electric field is dimensionless.

The 3D coherent PSF is:

$$
\begin{aligned}
h_c(\vec{r}) = h_c(\vec{r}_\perp, z) &= h_0(\vec{r}_\perp) \otimes_{2D} h_z(\vec{r}_\perp, z), \\
&= h_0(\vec{r}_\perp) \otimes_{2D} \frac{e^{ikr}}{4\pi r}\left[\frac{ikz}{r} - \frac{z}{r^2}\right]
\end{aligned}
\tag{16}
$$

where $h_0(\vec{r}_\perp)$ is the 2D in-focus PSF that accounts for the finite aperture of the imaging system, $h_z(\vec{r}_\perp, z)$ is the 2D "propagation spread function" that models the blur caused by the propagation of the electric field from defocus z to the image plane, and $\otimes_{2D}$ denotes 2D convolution.

To understand the image formation model (15), we take its 3D Fourier transform along $\vec{r}$ dimension:

$$
\tilde{E}_{\text{out},p}(\vec{u}, \vec{v}_\perp) = \left\{ E_{0p}(\vec{v}_\perp)\delta(\vec{u}-\vec{v}) + \sum_{q=x,y,z} \tilde{G}_{pq}(\vec{u}) \left[\sum_{\ell=x,y,z} \tilde{f}_{q\ell}(\vec{u}-\vec{v}) E_{0\ell}(\vec{v}_\perp) \right] \right\} \tilde{h}_c(\vec{u}),
\tag{17}
$$

where, a quantity $\tilde{x}(\vec{u})$ is the 3D Fourier transform of $x(\vec{r})$. Note that the Fourier transform of the 3D coherent PSF, i.e., $\tilde{h}_c(\vec{u})$ is the 3D coherent transfer function (CTF). We visualize this coherent image formation model in Fourier space in Figure 1. It is worth noting that the output electric field has zero bandwidth along the u_z direction. This implies that there is no axial sectioning when the image is formed with a single plane wave from a point source (a coherent mode). The axial resolution of PTI is built up by probing the specimen simultaneously with multiple plane waves generated by the partially coherent illumination source, which we discuss next.

1.4.3. *Scattered vector field when the specimen is illuminated by a source pattern.* We just derived the scattered electric field at the detector plane from a coherent point source at $\vec{v}_\perp$. Different partially coherent source patterns (α) are specified by different sets of $\vec{v}_\perp$. We represent the vector electric field in terms of the Stokes parameters as we did in our earlier work [22, 23]. By integrating the contribution of all the coherent scattered modes from one illumination pattern, we obtain the Stokes parameters [3, 4] as

$$
\begin{aligned}
&\begin{bmatrix} S_{0,\alpha}(\vec{r}) \\ S_{1,\alpha}(\vec{r}) \\ S_{2,\alpha}(\vec{r}) \\ S_{3,\alpha}(\vec{r}) \end{bmatrix} = \begin{bmatrix} S_{xx,\alpha}(\vec{r}) + S_{yy,\alpha}(\vec{r}) \\ S_{xx,\alpha}(\vec{r}) - S_{yy,\alpha}(\vec{r}) \\ S_{xy,\alpha}(\vec{r}) + S_{yx,\alpha}(\vec{r}) \\ i\left[S_{xy,\alpha}(\vec{r}) - S_{yx,\alpha}(\vec{r})\right] \end{bmatrix}, \\
&\text{where} \quad S_{pq,\alpha}(\vec{r}) = \iint_{\vec{v}_\perp \in \alpha} E_{\text{out},p}(\vec{r}, \vec{v}_\perp) E^*_{\text{out},q}(\vec{r}, \vec{v}_\perp) d^2\vec{v}_\perp.
\end{aligned}
\tag{18}
$$

Up to this point, we have arrived at a rigorous vectorial single-scattering imaging model termed, *vector Born model*, which we use to generate all of our simulated data in this paper. Since the Stokes parameters undergo the integration along $\vec{v}_\perp$, the dimension of $S_{pq,\alpha}(\vec{r})$ is $[L^{-2}]$.

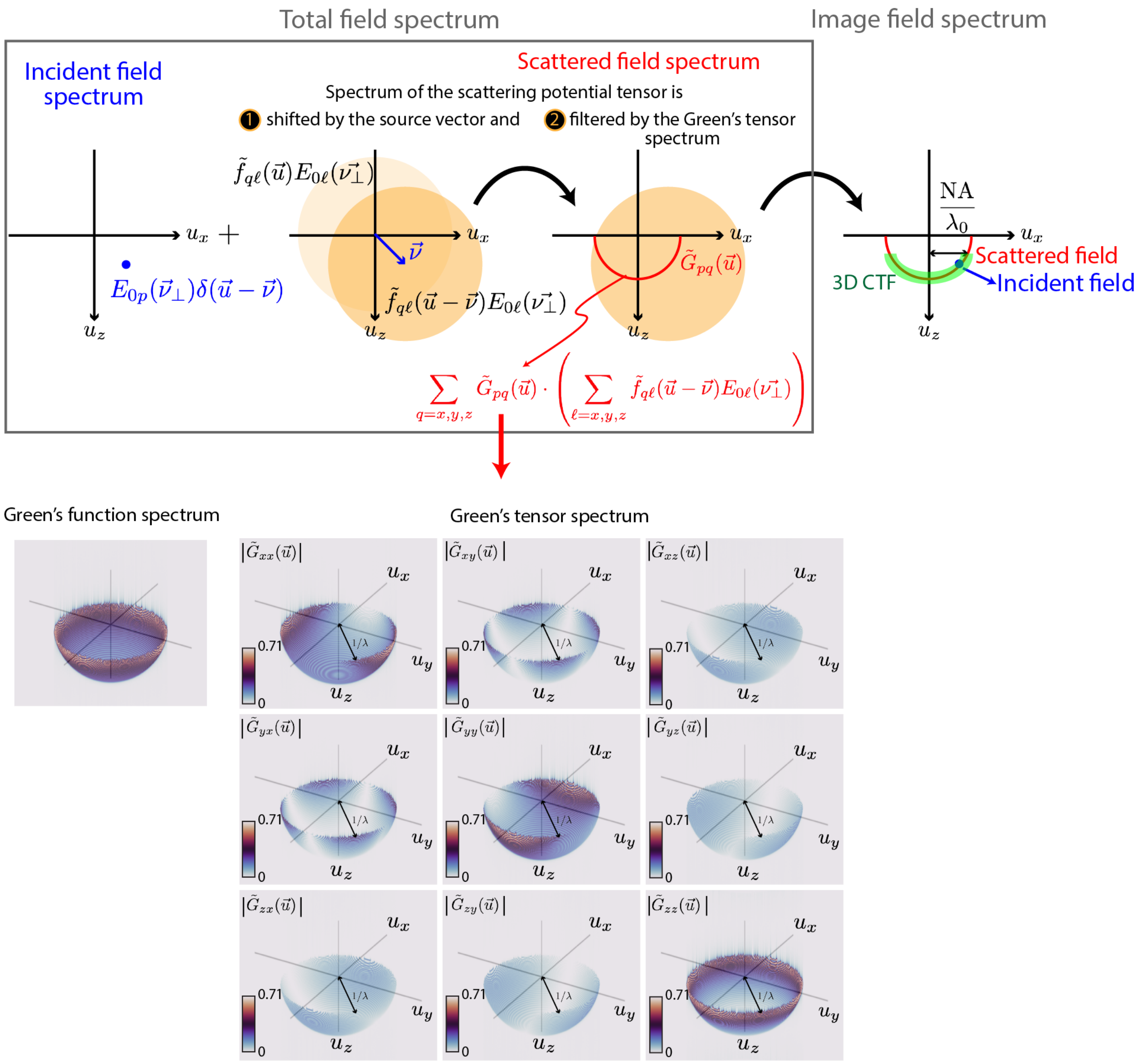

FIGURE 1. **Illustration of the coherent vector imaging model in Fourier space:** The above figure illustrates quantities in (17). The incident field (blue) is a 3D delta function located at the spatial frequency of illumination, $\vec{\nu}$. The scattered field spectrum (red) encodes the information of the scattering potential tensor spectrum (①) localized on the shell of the Ewald sphere defined by the Green's tensor components(②) shown at the bottom. The spectrum of the scattering potential tensor is filtered by the spectrum of Green's tensor. Since we only consider forward scattering, this shell is only half of the Ewald sphere [12]. Summing the incident field and the scattered field, the total field is also located on the half sphere. The imaging path filters the spectrum of the total field with 3D CTF, $\tilde{h}_c(\vec{u})$, which effectively truncates transverse spatial frequencies beyond NA/λ_0.

1.4.4. *Linearized vector Born model.* For the subsequent analysis, we take a closer look at the Fourier transform of $S_{pq,\alpha}(\vec{r})$

$$
\begin{aligned}
\tilde{S}_{pq,\alpha}(\vec{u}) &= \iint_{\vec{v}_\perp \in \alpha} \tilde{E}_{\text{out},p}(\vec{u},\vec{v}_\perp) \otimes \tilde{E}^*_{\text{out},q}(-\vec{u},\vec{v}_\perp) d^2\vec{v}_\perp \\
&= \iint_{\vec{v}_\perp \in \alpha} d^2\vec{v}_\perp \left\{ \left[E_{0p}(\vec{v}_\perp)\delta(\vec{u}-\vec{v}) + \sum_{\beta=x,y,z} \tilde{G}_{p\beta}(\vec{u}) \left(\sum_{\gamma=x,y,z} \tilde{f}_{\beta\gamma}(\vec{u}-\vec{v}) E_{0\gamma}(\vec{v}_\perp) \right) \right] \tilde{h}_c(\vec{u}_\perp) \right\} \\
&\quad \otimes \left\{ \left[E^*_{0q}(\vec{v}_\perp)\delta(-\vec{u}-\vec{v}) + \sum_{\xi=x,y,z} \tilde{G}^*_{q\xi}(-\vec{u}) \left(\sum_{\eta=x,y,z} \tilde{f}^*_{\xi\eta}(-\vec{u}-\vec{v}) E^*_{0\eta}(\vec{v}_\perp) \right) \right] \tilde{h}^*_c(-\vec{u}_\perp) \right\}
\end{aligned} \tag{19}
$$

where $\tilde{a}(\vec{u})$ denotes the Fourier transform of a function $a(\vec{r})$ at the 3D spatial frequency $\vec{u} = [\vec{u}_\perp^T, u_z]^T$. From this equation, we observe that the spatial frequencies of Stokes parameters that we can measure, $\tilde{S}_{pq,\alpha}(\vec{u})$, are non-linearly related to spatial frequencies of the scattering potential tensor components, $\tilde{f}_{pq}(\vec{u})$. Rigorous estimation of the scattering potential tensor from measured Stokes volumes requires an iterative optimization algorithm as in the classic phase retrieval problem [24], which can be time consuming and subject to convergence instability [25]. Instead, we adopt the approach of linearizing the equation for simpler, faster, and more robust solution.

To linearize the relationship between the measured Stokes components and the scattering potential tensor components, we expand (19) into three terms that describe the major contributions of scattered light

$$\tilde{S}_{pq,\alpha}(\vec{u}) = \tilde{S}_{pq,\alpha,\text{DC}}(\vec{u}) + \tilde{S}_{pq,\alpha,\text{L}}(\vec{u}) + \tilde{S}_{pq,\alpha,\text{NL}}(\vec{u}) \tag{20}$$

$\tilde{S}_{pq,\alpha,\text{DC}}$ is the constant component of a Stokes parameters, $\tilde{S}_{pq,\alpha}(\vec{u})$, due to the incident electric field. $\tilde{S}_{pq,\alpha,\text{L}}$ is the term that linearly relates the scattering potential tensor with the Stokes parameters. The linear term is due to the interference of the scattered electric field and the incident electric field. $\tilde{S}_{pq,\alpha,\text{NL}}$ is due to interference between scattered fields, which is the weakest among three terms and non-linearly related to the scattering potential tensor.

When imaging transparent biological specimens, most of the contrast in the Stokes parameters is contributed by the constant and the linear terms. We assume the contribution from the interference of the scattered field with itself is negligible. Neglecting the nonlinear term and subtracting the constant term from $\tilde{S}_{pq,\alpha}(\vec{u})$, we work only with the linear term as

$$\tilde{S}'_{pq,\alpha}(\vec{u}) = \tilde{S}_{pq,\alpha}(\vec{u}) - \tilde{S}_{pq,\alpha,\text{DC}}(\vec{u}) \approx \tilde{S}_{pq,\alpha,\text{L}}(\vec{u}) \tag{21}$$

After this linearization, we find that the Stokes parameters are a sum of filtered scattering potential tensor components. These filters can be thought of as a *filter bank* of weak object transfer functions widely used in the scalar wave scattering theory [16, 17, 18, 19, 20, 26].

We write the scattering potential tensor components in terms of the mean permittivity, differential permittivity, and 3D orientation of the material:

$$
\begin{aligned}
&f_{xx}(\vec{r}) = f_0(\vec{r}) + f_{1\text{c}}(\vec{r}), \quad f_{yy}(\vec{r}) = f_0(\vec{r}) - f_{1\text{c}}(\vec{r}), \quad f_{zz}(\vec{r}) = f_0(\vec{r}) + f_3(\vec{r}), \\
&f_{xy}(\vec{r}) = f_{yx}(\vec{r}) = f_{1\text{s}}(\vec{r}), \quad f_{yz}(\vec{r}) = f_{zy}(\vec{r}) = f_{2\text{s}}(\vec{r}), \quad f_{zx}(\vec{r}) = f_{xz}(\vec{r}) = f_{2\text{c}}(\vec{r}),
\end{aligned}
$$

$$
\text{where} \quad \left\{ \begin{aligned}
f_0(\vec{r}) &= k_0^2 \left[\varepsilon_\text{r}(\vec{r}) - \varepsilon_\text{rm} - \Delta\varepsilon_\text{r}(\vec{r}) \cos^2\theta(\vec{r}) \right] \\
f_{1\text{c}}(\vec{r}) &= k_0^2 \Delta\varepsilon_\text{r}(\vec{r}) \sin^2\theta(\vec{r}) \cos 2\omega(\vec{r}) \\
f_{1\text{s}}(\vec{r}) &= k_0^2 \Delta\varepsilon_\text{r}(\vec{r}) \sin^2\theta(\vec{r}) \sin 2\omega(\vec{r}) \\
f_{2\text{c}}(\vec{r}) &= k_0^2 \Delta\varepsilon_\text{r}(\vec{r}) \sin 2\theta(\vec{r}) \cos\omega(\vec{r}) \\
f_{2\text{s}}(\vec{r}) &= k_0^2 \Delta\varepsilon_\text{r}(\vec{r}) \sin 2\theta(\vec{r}) \sin\omega(\vec{r}) \\
f_3(\vec{r}) &= k_0^2 \Delta\varepsilon_\text{r}(\vec{r}) \left[3\cos^2\theta(\vec{r}) - 1 \right]
\end{aligned} \right. \tag{22}
$$

The interpretation of the above parameterization of the scattering potential tensor is as follows:

- $f_0(\vec{r}) = f_{0\mathrm{r}}(\vec{r}) + i f_{0\mathrm{i}}(\vec{r})$ is a complex function representing density (complex-valued mean permittivity) of the material. The real part (Re $\{\varepsilon_\mathrm{r}(\vec{r}) - \varepsilon_\mathrm{rm}\}$) delays and the imaginary part (Im $\{\varepsilon_\mathrm{r}(\vec{r}) - \varepsilon_\mathrm{rm}\}$) absorbs the light.
- $f_{1\mathrm{c}}(\vec{r}), f_{1\mathrm{s}}(\vec{r})$, $f_{2\mathrm{c}}(\vec{r})$, $f_{2\mathrm{s}}(\vec{r})$, and $f_3(\vec{r})$ are real-valued functions that encode differential permittivity ($\Delta\varepsilon_\mathrm{r}(\vec{r})$), in-plane orientation ($\omega(\vec{r})$), and inclination ($\theta(\vec{r})$). We ignore the effect of diattenuation.

Each scattering potential tensor component is linked to the Stokes components via a set of transfer functions. However, the actual Stokes parameters in (18) are a sum or a difference of the Stokes components.

We summarize the linear relationship between the Stokes parameters and the scattering potential tensor in one equation as

$$\tilde{S}'_{m,\alpha}(\vec{u}) = \sum_{\substack{\ell=0\mathrm{r},0\mathrm{i},1\mathrm{c},\\1\mathrm{s},2\mathrm{c},2\mathrm{s},3}} \tilde{H}_{m,\ell,\alpha}(\vec{u})\tilde{f}_\ell(\vec{u}); \quad m = 0,1,2,3, \tag{23}$$

where $\tilde{S}'_{m,\alpha}(\vec{u})$ is the DC-subtracted Stokes parameter and $\tilde{H}_{m,\ell,\alpha}(\vec{u})$ is the transfer function mapping from each tensor component ℓ to the m-th Stokes parameter under illumination pattern α. The transfer functions are dimensionless. Thus, the dimensions throughout the derivation are consistent because the scattering potential tensor and the Stokes parameters have the same dimensions ($[L^{-2}]$).

The equation above describes the *linearized vector Born model* that we use for reconstruction of the scattering potential tensor.

1.4.5. *Reduction to 2D imaging model.* When the thickness of a specimen is smaller than the depth of field of a certain imaging condition, acquiring data at one plane by changing the partially coherent illumination patterns enables extraction of 2D phase and anisotropic information at that plane. This 2D information extraction requires a slight modification of the transfer functions. In this 2D imaging case, we consider the scattering potential tensor to be concentrated in a single axial layer and is expressed as $\overline{\overline{f}}(\vec{r}_\perp)$ with the Fourier transform $\tilde{f}_\ell(\vec{u}_\perp)$.

We then substitute this expression for $\tilde{f}_\ell(\vec{u})$ in (23) and integrate over the spatial frequency u_z to get a corresponding relationship in the 2D imaging case between 2D Stokes parameters and the 2D scattering potential tensor as

$$\tilde{S}'^{(z=0)}_{m,\alpha}(\vec{u}_\perp) = \int \tilde{S}'_{m,\alpha}(\vec{u})du_z = \sum_{\substack{\ell=0\mathrm{r},0\mathrm{i},1\mathrm{c},\\1\mathrm{s},2\mathrm{c},2\mathrm{s},3}} \left[\int \tilde{H}_{m,\ell,\alpha}(\vec{u})du_z\right] \tilde{f}_\ell(\vec{u}_\perp); \quad m = 0,1,2,3. \tag{24}$$

The transfer functions in 2D imaging case are simply a u_z-integration of the previously established 3D transfer functions.

2. Computing Stokes parameters from intensities

In fig. 1, we have demonstrated that the raw images from the PTI setup contain intensity modulation induced by the specimen's permittivity tensor. In Supplementary Note 1, we established a imaging model to describe the relationship between components of specimen's permittivity tensor and the Stokes parameters of the output scattered light. Here, we relate the Stokes parameters of the scattered light to the measured intensities.

2.1. **Calibration.** Ideally, our polarization sensitive camera perfectly detects four channels of linearly polarized light oriented at 0°, 45°, 90°, and 135°. According to the definition of the Stokes vector [27], the measured intensity is linearly related to the Stokes parameters in the following form

$$\vec{I} = \begin{bmatrix} I_0 \\ I_{45} \\ I_{90} \\ I_{135} \end{bmatrix} = \frac{1}{2}\begin{bmatrix} 1 & 1 & 0 & 0 \\ 1 & 0 & 1 & 0 \\ 1 & -1 & 0 & 0 \\ 1 & 0 & -1 & 0 \end{bmatrix} \cdot \begin{bmatrix} S_0 \\ S_1 \\ S_2 \\ S_3 \end{bmatrix} = \overline{\overline{A}} \cdot \vec{S}, \tag{25}$$

where $\vec{I}$ is the intensity vector of each polarization measurement, $\overline{\overline{A}}$ is the instrument matrix [28] of the optical system, and $\vec{S}$ is Stokes vector of the light. Applying the inverse of the known instrument matrix, $\overline{\overline{A}}$, on the measured intensity vector, $\vec{I}$, allows us to get the first three Stokes parameters for further deconvolution. However, the actual optical setup has multiple components (e.g. beam splitting prisms) with non-ideal polarization effects. These components are modeled by the non-ideal values in the instrument matrix. Thus, calibration of the instrument matrix is essential. To calibrate the unknown instrument matrix, we adopt the approach in [28, 29]. We place a linear polarizer mounted on a rotational stage at the specimen plane and rotate it to collect corresponding intensity variations in four polarization channels. We model the intensities collected at angle ω_{LP} of the linear polarizer and retrieve the instrument matrix using Mueller matrix calculus. In addition, we calibrate the polarization state of the incident light by extending the calibration process described by earlier methods.

Assuming the incident light has the Stokes parameters of $\vec{S}_s = [S_{s0}, S_{s1}, S_{s2}, S_{s3}]^T$, the generated polarization state of light after the linear polarizer aligned with angle ω_{LP} in Stokes parameters is calculated as

$$\begin{aligned} \vec{S}_{\mathrm{LP}}(\omega_{\mathrm{LP}}) &= \overline{\overline{M}}_{\mathrm{LP}}(\omega_{\mathrm{LP}}) \cdot \vec{S}_s = S_{\mathrm{LP},0}(\omega_{\mathrm{LP}}) \cdot \begin{bmatrix} 1 \\ \cos 2\omega_{\mathrm{LP}} \\ \sin 2\omega_{\mathrm{LP}} \\ 0 \end{bmatrix}, \\ \text{where } S_{\mathrm{LP},0}(\omega_{\mathrm{LP}}) &= S_{s0} + S_{s1} \cdot \cos 2\omega_{\mathrm{LP}} + S_{s2} \cdot \sin 2\omega_{\mathrm{LP}} \end{aligned} \tag{26}$$

$\overline{\overline{M}}_{\mathrm{LP}}(\alpha)$ is the Mueller matrix of a linear polarizer aligned at angle ω_{LP}. This formula shows that the total energy of the generated state, $S_{\mathrm{LP},0}(\omega_{\mathrm{LP}})$, will be a constant along ω_{LP} if the input light is unpolarized or circularly polarized. This total energy can vary if our input light has some linear polarization components. We estimate the total energy of the generated light by summing over intensities of four polarization channel. Through a Fourier series fitting, we obtain the Stokes parameters of the incident light and use it for later deconvolution.

The above polarization state is analyzed by the detection path. The measured intensity is a matrix multiplication of the instrument matrix and the generated Stokes parameters

$$\vec{I}(\omega_{\mathrm{LP}}) = \overline{\overline{A}}\vec{S}_{\mathrm{LP}}(\omega_{\mathrm{LP}}) = S_{\mathrm{LP},0}(\omega_{\mathrm{LP}}) \cdot (\vec{a}_0 + \vec{a}_1 \cdot \cos 2\omega_{\mathrm{LP}} + \vec{a}_2 \cdot \sin 2\omega_{\mathrm{LP}}), \tag{27}$$

where $\vec{a}_i$ is the $i+1$-th column of the instrument matrix $\overline{\overline{A}}$. If we normalize the measured intensity with the total energy of the generated light, $S_{\mathrm{LP},0}(\omega_{\mathrm{LP}})$, we observe that the first three columns of instrument matrix are the Fourier series coefficients of the normalized intensities along ω_{LP}. We use this model to compute the instrument matrix by curve fitting.

With the first three columns of the instrument matrix, we obtain the first three Stokes parameters of the light as

$$\begin{bmatrix} S_0 \\ S_1 \\ S_2 \end{bmatrix} = \begin{bmatrix} \vec{a}_0 & \vec{a}_1 & \vec{a}_2 \end{bmatrix}^+ \vec{I}, \tag{28}$$

where A^+ denotes a pseudo-inverse of the matrix A.

2.2. **Background correction.** The Stokes parameters estimated with the instrument matrix calibration may still be affected by the residual polarization effects in the light path that introduce slowly varying background. In addition, the deconvolution starts with the DC-subtracted Stokes parameters according to (6).

We image an empty field of view to characterize the slowly varying background. We correct the background and obtain the DC-subtracted Stokes parameters as follows.

$$\begin{bmatrix} S'_0 \\ S'_1 \\ S'_2 \end{bmatrix} = \begin{bmatrix} S_0 \\ S_1 \\ S_2 \end{bmatrix} \cdot \frac{1}{S_{\mathrm{bg},0}} - \begin{bmatrix} 0 \\ S_{\mathrm{bg},1} \\ S_{\mathrm{bg},2} \end{bmatrix} \cdot \frac{S_0}{S_{\mathrm{bg},0}^2} - \begin{bmatrix} 1 \\ 0 \\ 0 \end{bmatrix}, \tag{29}$$

where $S_{\text{bg,i}}$ is the i-th background Stokes parameters obtained when imaging at an empty field of view. When the transmission modulation of the specimen is relatively weak, $S_0/S_{\text{bg},0} \approx 1$ and this is simply removing the DC components of the Stokes parameters. These operations are done to all sets of polarization acquisitions with different illumination patterns and then the deconvolution takes these DC-subtracted Stokes parameters for the retrieval of uniaxial permittivity tensor components.

2.3. **Derivation of the Stokes background correction.** Since background variation is usually slowly varying, we adopt a non-diffraction-based Mueller calculus to develop our background removal scheme. An anisotropic sample with transmittance $t_s = |e^{\mu+i\phi}|^2$, in-plane orientation of ω, out-of-plane orientation of θ, apparent retardance $\rho_{\text{ap}}(\omega,\theta)$ can be modeled with an Mueller matrix as

$$\overline{\overline{M}}_s(t_s,\omega,\theta,\rho_{\text{ap}}) \approx t_s \begin{bmatrix} 1 & 0 & 0 & 0 \\ 0 & 1 & 0 & -\sin 2\omega \cdot \rho_{\text{ap}}(\omega,\theta) \\ 0 & 0 & 1 & \cos 2\omega \cdot \rho_{\text{ap}}(\omega,\theta) \\ 0 & \sin 2\omega \cdot \rho_{\text{ap}}(\omega,\theta) & -\cos 2\omega \cdot \rho_{\text{ap}}(\omega,\theta) & 1 \end{bmatrix}, \text{ when } \rho_{\text{ap}}(\omega,\theta) \ll 1 \tag{30}$$

Illuminating with the circularly polarized light with the Stokes parameter of $\vec{S}_s = S_{s0}\cdot[1,0,0,1]^T$, where S_{s0} is the energy of the incident light, the output Stokes parameters are obtained as

$$\vec{S}_{s,\text{out}} = \overline{\overline{M}}_s(t_s,\omega,\theta,\rho_{\text{ap}})\cdot\vec{S}_s = S_{s0}\cdot \begin{bmatrix} t_s \\ -t_s\cdot\sin 2\omega\cdot\rho_{\text{ap}}(\omega,\theta) \\ t_s\cdot\cos 2\omega\cdot\rho_{\text{ap}}(\omega,\theta) \\ t_s \end{bmatrix} \tag{31}$$

These are the Stokes parameters we get assuming we do not have additional background polarization effects from the optics in the microscope. When the optical path is not perfectly free of polarization effects, slowly varying polarization modulation can mask the weak anisotropy signal. To remove this effect, we model the background polarization effect as an additional transparent retarder with the Mueller matrix of $\overline{\overline{M}}_{\text{bg}}(t_{\text{bg}},\omega_{\text{bg}},\theta_{\text{bg}},\rho_{\text{bg}})$. With this background Mueller matrix, the overall output Stokes parameters are

$$\begin{aligned} \vec{S}_{\text{out}} = \begin{bmatrix} S_{\text{out},0} \\ S_{\text{out},1} \\ S_{\text{out},2} \\ S_{\text{out},3} \end{bmatrix} &= \overline{\overline{M}}_{\text{bg}}(t_{\text{bg}},\omega_{\text{bg}},\theta_{\text{bg}},\rho_{\text{bg}})\cdot\overline{\overline{M}}_s(t_s,\omega,\theta,\rho_{\text{ap}})\cdot\vec{S}_s \\ &\approx t_{\text{bg}}\cdot\vec{S}_{s,\text{out}} + S_{s0}\cdot \begin{bmatrix} 0 \\ -t_s\cdot t_{\text{bg}}\cdot\sin 2\omega_{\text{bg}}\cdot\rho_{\text{bg}} \\ t_s\cdot t_{\text{bg}}\cdot\cos 2\omega_{\text{bg}}\cdot\rho_{\text{bg}} \\ 0 \end{bmatrix} \end{aligned} \tag{32}$$

These overall Stokes parameters contain contributions from the specimen and the background. To separate these effects, we acquire the background Stokes parameters ($\vec{S}_{\text{bg}}$) by imaging an area without the specimen,

$$\vec{S}_{\text{bg}} = \begin{bmatrix} S_{\text{bg},0} \\ S_{\text{bg},1} \\ S_{\text{bg},2} \\ S_{\text{bg},3} \end{bmatrix} = \overline{\overline{M}}_{\text{bg}}(t_{\text{bg}},\omega_{\text{bg}},\theta_{\text{bg}},\rho_{\text{bg}})\cdot\vec{S}_s = S_{s0}\cdot \begin{bmatrix} t_{\text{bg}} \\ -t_{\text{bg}}\cdot\sin 2\omega_{\text{bg}}\cdot\rho_{\text{bg}} \\ t_{\text{bg}}\cdot\cos 2\omega_{\text{bg}}\cdot\rho_{\text{bg}} \\ t_{\text{bg}} \end{bmatrix} \tag{33}$$

Comparing (32) with (33), we can compute corrected Stokes parameters as

$$\vec{S}_c = \frac{\vec{S}_{s,\text{out}}}{S_{s0}} = \frac{\vec{S}_{\text{out}}}{S_{\text{bg},0}} - \begin{bmatrix} 0 \\ S_{\text{bg},1} \\ S_{\text{bg},2} \\ 0 \end{bmatrix}\cdot\frac{S_{\text{out},0}}{S_{\text{bg},0}^2} \tag{34}$$

With this correction, we also remove the DC components of the Stokes parameters in $S_{\text{out},1}$ and $S_{\text{out},2}$. The only remaining Stokes parameters with the DC-component is the S_0 component of $\vec{S}_c$. With the additional subtraction, we arrive at the final DC-subtracted Stokes parameters that we used for the deconvolution

$$\vec{S}' = \vec{S}_c - \begin{bmatrix} 1 \\ 0 \\ 0 \\ 1 \end{bmatrix} \tag{35}$$

We apply these corrections on all sets of intensities from each illumination patterns before deconvolving the data.

3. INVERSE ALGORITHM

3.1. **Solving for scattering potential tensor.** According to the imaging model (6), the DC-subtracted (background subtracted) Stokes parameters of the scattered light, $\tilde{S}'_{m,\alpha}(\vec{u})$, under different illuminations are linearly related to the entries of the scattering potential tensors. Thus, we can write down an inverse problem to retrieve the entries of the scattering potential tensor as

$$\min_{\tilde{f}_\ell(\vec{u})} \sum_\alpha \sum_{m=0}^{2} \left\| \tilde{S}'_{m,\alpha}(\vec{u}) - \sum_{\substack{\ell=0\mathrm{r},0\mathrm{i},1\mathrm{c},\\ 1\mathrm{s},2\mathrm{c},2\mathrm{s},3}} \tilde{H}_{m,\ell,\alpha}(\vec{u})\tilde{f}_\ell(\vec{u}) \right\|_2^2 + \sum_{\substack{\ell=0\mathrm{r},0\mathrm{i},1\mathrm{c},\\ 1\mathrm{s},2\mathrm{c},2\mathrm{s},3}} \tau_\ell \left\| \tilde{f}_\ell(\vec{u}) \right\|_2^2, \tag{36}$$

where τ_ℓ is the Tikhonov regularization parameter for each independent term in the scattering potential tensor. In our implementation, this optimization is done in the Fourier space, where each voxel (or pixel) in Fourier space is independent from other voxels (or pixels).

We speed up computation of (36) by solving the point-wise optimization problems at all spatial frequencies $\vec{u}$ in parallel. The least square solution of this one-point optimization can be obtained by solving the fully determined inverse problem of

$$\left[\left(\sum_\alpha \overline{\overline{\tilde{H}}}_\alpha^\dagger \overline{\overline{\tilde{H}}}_\alpha \right) + \mathrm{diag}(\vec{\tau}) \right] \vec{\tilde{f}} = \overline{\overline{\mathscr{H}}}\vec{\tilde{f}} = \sum_\alpha \overline{\overline{\tilde{H}}}_\alpha^\dagger \vec{\tilde{S}}'_\alpha, \tag{37}$$

where

$$\begin{aligned}
\overline{\overline{\tilde{H}}}_\alpha &= \begin{bmatrix} \tilde{H}_{0,0\mathrm{r},\alpha} & \tilde{H}_{0,0\mathrm{i},\alpha} & \tilde{H}_{0,1\mathrm{c},\alpha} & \tilde{H}_{0,1\mathrm{s},\alpha} & \tilde{H}_{0,2\mathrm{c},\alpha} & \tilde{H}_{0,2\mathrm{s},\alpha} & \tilde{H}_{0,3,\alpha} \\ \tilde{H}_{1,0\mathrm{r},\alpha} & \tilde{H}_{1,0\mathrm{i},\alpha} & \tilde{H}_{1,1\mathrm{c},\alpha} & \tilde{H}_{1,1\mathrm{s},\alpha} & \tilde{H}_{1,2\mathrm{c},\alpha} & \tilde{H}_{1,2\mathrm{s},\alpha} & \tilde{H}_{1,3,\alpha} \\ \tilde{H}_{2,0\mathrm{r},\alpha} & \tilde{H}_{2,0\mathrm{i},\alpha} & \tilde{H}_{2,1\mathrm{c},\alpha} & \tilde{H}_{2,1\mathrm{s},\alpha} & \tilde{H}_{2,2\mathrm{c},\alpha} & \tilde{H}_{2,2\mathrm{s},\alpha} & \tilde{H}_{2,3,\alpha} \end{bmatrix}, \\
\vec{\tau} &= \begin{bmatrix} \tau_{0\mathrm{r}} & \tau_{0\mathrm{i}} & \tau_{1\mathrm{c}} & \tau_{1\mathrm{s}} & \tau_{2\mathrm{c}} & \tau_{2\mathrm{s}} & \tau_3 \end{bmatrix}^T, \\
\vec{\tilde{f}} &= \begin{bmatrix} \tilde{f}_{0\mathrm{r}} & \tilde{f}_{0\mathrm{i}} & \tilde{f}_{1\mathrm{c}} & \tilde{f}_{1\mathrm{s}} & \tilde{f}_{2\mathrm{c}} & \tilde{f}_{2\mathrm{s}} & \tilde{f}_3 \end{bmatrix}^T, \\
\vec{\tilde{S}}'_\alpha &= \begin{bmatrix} \tilde{S}'_{0,\alpha} & \tilde{S}'_{1,\alpha} & \tilde{S}'_{2,\alpha} \end{bmatrix}^T, \\
\overline{\overline{\mathscr{H}}} &= \left(\sum_\alpha \overline{\overline{\tilde{H}}}_\alpha^\dagger \overline{\overline{\tilde{H}}}_\alpha \right) + \mathrm{diag}(\vec{\tau}),
\end{aligned} \tag{38}$$

$\overline{\overline{\tilde{H}}}_\alpha^\dagger$ denotes the Hermitian of the matrix $\overline{\overline{\tilde{H}}}_\alpha$. With this fully determined inverse problem, we apply the Cramer's rule on it to obtain the analytical solution as

$$\tilde{f}_\ell = \det\left(\overline{\overline{\mathscr{H}}}_\ell\right) \Big/ \det\left(\overline{\overline{\mathscr{H}}}\right), \tag{39}$$

where $\overline{\overline{\mathscr{H}}}_\ell$ denotes that the ℓ-th column of the $\overline{\overline{\mathscr{H}}}$ matrix is replaced with $\sum_\alpha \overline{\overline{\tilde{H}}}_\alpha^\dagger \vec{\tilde{S}}'_\alpha$.

We set the regularization τ_ℓ as a multiple of the mean absolute value of the ℓ-th diagonal entry of $\overline{\overline{\mathscr{H}}}$ over all spatial frequency $\vec{u}$ to reduce its dependency on different imaging parameters. Usually the multiple factor of τ_ℓ is ranging from 1 to 100 (smaller for less noisy data to get sharper reconstruction and larger for more noisy data to smooth out the noise). It should also be noted that, for simplicity of the math, we only show the single point solution of this inverse problem. In the actual implementation, we vectorize the whole 3D array of the data and the transfer functions for fast parallel processing with GPU.

3.2. **Mean permittivity, differential permittivity and 3D orientation from scattering potential tensor.** After solving (36), we obtain components of scattering potential tensor as shown in (3). We report the 2D or 3D mean and differential permittivities in terms of the angular average of permittivity $(\varepsilon_\mathrm{r} - \varepsilon_\mathrm{rm})$ and the angular difference of the

permittivity ($\Delta\varepsilon_r$), respectively. Because of this difference in 2D and 3D imaging, we present solutions for both situations.

We start this step of the reconstruction by estimating the differential permittivity and 3D orientation from the 1c, 1s, 2c, 2s terms in the scattering potential tensor. This step requires information of the optic sign ($n_e > n_o$ or $n_e < n_o$). Here, we first compute two analytical solutions for differential permittivity and 3D orientation assuming two optic signs. These two sets of solutions will be used to estimate the probability of the optic signs in the next part of the algorithm. Using trigonometry relations, we express these two solutions of differential permittivity and 3D orientation in 2D and 3D imaging modes as

$$
\begin{array}{ll}
\text{2D}: & \\
\omega_{\pm}(\vec{r}_{\perp}) = \frac{1}{2}\tan^{-1}\left[\frac{\pm g_{1s}(\vec{r}_{\perp})}{\pm g_{1c}(\vec{r}_{\perp})}\right], & \text{when } n_e \gtrless n_o \\
\theta_{\pm}(\vec{r}_{\perp}) = \tan^{-1}\left\{\frac{\pm 2[g_{1s}(\vec{r}_{\perp})\sin 2\omega_{\pm}(\vec{r}_{\perp})+g_{1c}(\vec{r}_{\perp})\cos 2\omega_{\pm}(\vec{r}_{\perp})]}{\pm[g_{2s}(\vec{r}_{\perp})\sin\omega_{\pm}(\vec{r}_{\perp})+g_{2c}(\vec{r}_{\perp})\cos\omega_{\pm}(\vec{r}_{\perp})]}\right\}, & \text{when } n_e \gtrless n_o \\
\rho_{2D,\pm}(\vec{r}_{\perp}) = \frac{1}{\sqrt{\varepsilon_{rm}}}\int \Delta\varepsilon_{r,\pm}(\vec{r})dz = \frac{[g_{1s}(\vec{r}_{\perp})\sin 2\omega_{\pm}(\vec{r}_{\perp})+g_{1c}(\vec{r}_{\perp})\cos 2\omega_{\pm}(\vec{r}_{\perp})]\sin^2\theta_{\pm}(\vec{r}_{\perp})}{k_0^2\sqrt{\varepsilon_{rm}}\left[\sin^4\theta_{\pm}(\vec{r}_{\perp})+\sigma\right]} & \text{when } n_e \gtrless n_o \\
\text{3D}: & \\
\omega_{\pm}(\vec{r}) = \frac{1}{2}\tan^{-1}\left[\frac{\pm f_{1s}(\vec{r})}{\pm f_{1c}(\vec{r})}\right], & \text{when } n_e \gtrless n_o \\
\theta_{\pm}(\vec{r}) = \tan^{-1}\left\{\frac{\pm 2[f_{1s}(\vec{r})\sin 2\omega_{\pm}(\vec{r})+f_{1c}(\vec{r})\cos 2\omega_{\pm}(\vec{r})]}{\pm[f_{2s}(\vec{r})\sin\omega_{\pm}(\vec{r})+f_{2c}(\vec{r})\cos\omega_{\pm}(\vec{r})]}\right\}, & \text{when } n_e \gtrless n_o \\
\rho_{3D,\pm}(\vec{r}) = \Delta\varepsilon_{r,\pm}(\vec{r}) = \frac{[f_{1s}(\vec{r})\sin 2\omega_{\pm}(\vec{r})+f_{1c}(\vec{r})\cos 2\omega_{\pm}(\vec{r})]\sin^2\theta_{\pm}(\vec{r})}{k_0^2\left[\sin^4\theta_{\pm}(\vec{r})+\sigma\right]} & \text{when } n_e \gtrless n_o
\end{array}, \tag{40}
$$

where $\omega_{\pm}$, $\theta_{\pm}$, and $\rho_{\pm}$ are in-plane orientation, inclination, and differential permittivity of the positive or negative uniaxial material. Note that $\rho_{2D,\pm}(\vec{r}_{\perp})$ is a 2D image with a unit of a distance at each pixel (difference of optical path length), while $\rho_{3D,\pm}(\vec{r})$ is a 3D volume without a unit for each voxel. σ is a small number to prevent noise amplification in the estimation of differential permittivity. Typically, $\sigma = 10^{-2} \sim 10^{-3}$ is a good choice to balance between accuracy and noise amplification.

The 0 term in the scattering potential tensor is related to the difference of average permittivity between the specimen and the environment, which corresponds to accumulated optical phase and absorption information. However, this term is coupled with the differential permittivity, $\Delta\varepsilon_r$. Removing the contribution of the differential permittivity, we express two solutions of phase and absorption in 2D and 3D imaging modes as

$$
\begin{array}{ll}
\text{2D}: & \\
\phi_{2D,\pm}(\vec{r}_{\perp}) = \frac{1}{2k_0^2\sqrt{\varepsilon_{rm}}}\mathrm{Re}\left\{g_0(\vec{r}_{\perp}) + k_0^2\left[\int \Delta\varepsilon_{r,\pm}(\vec{r})dz\right]\cos\theta_{\pm}(\vec{r}_{\perp})\right\}, & \text{when } n_e \gtrless n_o \\
\mu_{2D,\pm}(\vec{r}_{\perp}) = -\frac{1}{2k_0^2\sqrt{\varepsilon_{rm}}}\mathrm{Im}\left\{g_0(\vec{r}_{\perp}) + k_0^2\left[\int \Delta\varepsilon_{r,\pm}(\vec{r})dz\right]\cos\theta_{\pm}(\vec{r}_{\perp})\right\}, & \text{when } n_e \gtrless n_o \\
\text{3D}: & \\
\phi_{3D,\pm}(\vec{r}) = \frac{1}{k_0^2}\mathrm{Re}\left\{f_0(\vec{r}) + k_0^2\Delta\varepsilon_{r,\pm}(\vec{r})\cos\theta_{\pm}(\vec{r})\right\}, & \text{when } n_e \gtrless n_o \\
\mu_{3D,\pm}(\vec{r}) = -\frac{1}{k_0^2}\cdot\mathrm{Im}\left\{f_0(\vec{r}) + k_0^2\Delta\varepsilon_{r,\pm}(\vec{r})\cos\theta_{\pm}(\vec{r})\right\}, & \text{when } n_e \gtrless n_o
\end{array}, \tag{41}
$$

where $\phi_{\pm}$ and $\mu_{\pm}$ are phase and absorption of the positive or negative uniaxial material. Note that $\phi_{2D,\pm}(\vec{r}_{\perp})$ is a 2D image with a unit of a distance at each pixel (optical path length), while $\phi_{3D,\pm}(\vec{r})$ is a 3D volume without a unit for each voxel.

At the end of this computation, the range of the in-plane orientation is $\omega \in [0,\pi)$ and the range of the inclination is $\theta \in [0,\pi)$. These ranges correspond to the front half of the hemisphere of unit sphere ($y \geq 0$). Depending on how we visualize the 3D orientation, we can also transform (ω,θ) coordinates to span the range of the top hemisphere (Extended Data Fig. 2B, $z \geq 0$).

3.3. **Optic sign from scattering potential tensor.** The optic sign reports symmetry of underlying structure. If we know of the type of the material imaged, we pick one set of solutions from $\omega_{\pm}$, $\theta_{\pm}$, and $\rho_{\pm}$. More often, the optic sign of a biological structure is not known and can be spatially variable.

For cases where the optic sign is unknown, we employ an algorithm that models the scattering potential as a mixture of positive uniaxial and negative uniaxial material. The algorithm starts with constructing the scattering potential tensor components with positive (ω_+, θ_+, ρ_+) and negative (ω_-, θ_-, ρ_-) set of solutions according to (3). We then want to know which set of solution is more favorable in our data by solving the following optimization problem

$$\min_{w_+(\vec{r}),w_-(\vec{r})} \sum_{\alpha} \sum_{m=0}^{2} \left\| \tilde{S}'_{m,\alpha}(\vec{u}) - \sum_{\substack{\ell=0\text{r},0\text{i},1\text{c},\\ 1\text{s},2\text{c},2\text{s},3}} \tilde{H}_{m,\ell,\alpha}(\vec{u}) \mathscr{F}\left\{ w_+(\vec{r}) f_{\ell,+}(\vec{r}) + w_-(\vec{r}) f_{\ell,-}(\vec{r}) \right\} \right\|_2^2, \tag{42}$$

where $w_+(\vec{r})$ and $w_-(\vec{r})$ are the weights for positive and negative uniaxial solutions (we only consider positive values of the weight). When the positive material is more favorable (the structure within a voxel is denser along axis of symmetry), $w_+(\vec{r})$ is larger than $w_-(\vec{r})$. On the other hand, $w_-(\vec{r})$ is larger than $w_+(\vec{r})$ when negative material is more favorable (the structure is denser perpendicular to the symmetry axis). When the material is isotropic, $w_+(\vec{r}) \approx w_-(\vec{r})$. We implement a gradient descent iterative algorithm to solve this optimization. To identify material type with these two weights, we define the probability of being positive material to be

$$p_+(\vec{r}) = \frac{\max\left[\frac{w_+(\vec{r})}{|w_+(\vec{r})|+|w_-(\vec{r})|}, w_c\right]}{\max\left[\frac{w_+(\vec{r})}{|w_+(\vec{r})|+|w_-(\vec{r})|}, w_c\right] + \max\left[\frac{w_-(\vec{r})}{|w_+(\vec{r})|+|w_-(\vec{r})|}, w_c\right]}, \tag{43}$$

where w_c is a cut-off weight to remove noisy weight estimate below this value for smooth probability reconstruction. w_c is usually set around $0.05 \sim 0.2$. The higher the value, the stronger we suppress the noisy estimate.

4. Image processing: multi-scale analysis, denoising, structure tensor analysis

4.1. **Multi-scale analysis of optical properties by averaging the permittivity tensor.** In fig. 4, we describe measurements of optical properties of the mouse brain tissue at multiple scales. Computing physically meaningful measurements of mean permittivity, differential permittivity, 3D orientation, and optic sign at lower spatial resolution from the high-resolution acquisition requires a specific filtering approach. The reconstruction steps that lead to components of the scattering potential tensor (39) are linear. However, the computation of optical properties from the scattering potential tensor components involves non-linear operations ((40) and (43)). Therefore, the low resolution measurements of optical properties are computed by linear filtering of the high resolution volumes of the scattering potential tensor, which are transformed into mean permittivity, differential permittivity, 3D orientation, and optic sign according to (40) and (43). This approach ensures that the computed optical properties at lower resolution are representative of an acquisition with a light path of lower spatial resolution.

4.2. **Improving the sensitivity of PTI through averaging and denoising.** PTI converts the intensity modulation from the density and anisotropy of the specimens into physical properties as shown in fig. 1C and 1D. These intensity modulations are built upon a constant intensity background that defines the transmission of the surrounding media. The ratio between the strength of the intensity modulation and the shot noise created by the constant background intensity defines the signal to noise ratio (SNR) of our measurements. Biological specimens that we work with generally have strong phase (average RI) and relatively weak anisotropy. Hence, SNR is typically lower for anisotropy measurements. In the following, we take two approaches to improve the SNR of our measurements.

The first approach is to reduce the shot noise in the raw intensity through averaging multiple frames. Since we cannot change the strength of anisotropy from a biological specimen, we average our raw intensity by N_{avg} frames to improve our SNR by $\sqrt{N_{\text{avg}}}$ times. N_{avg} varies by experiments. For strongly anisotropic structures such as the anisotropic glass target in fig. 3 and myelin sheaths in fig. 4 (differential permittivity $\gtrsim 0.01$ with the high-NA objective), $N_{\text{avg}} = 1$ already gives satisfying results. For weaker anisotropic structures such as iPSC-derived cardiomyocytes in fig. 5 and A549 cells in Figure 2 and 3, we average 32 frames and 100 frames per intensity acquisition, respectively, to increase the SNR.

The second approach is to apply an additional wavelet denoising on the differential permittivity after the inverse algorithm (36). Tikhonov regularizer in the existing inverse algorithm prevents over-amplification of noise in the reconstructed physical properties. Total-variation or wavelet-based denoising algorithms that leverage the continuity of the images could provide additional improvements in the SNR of the image. However, a proper implementation of these algorithms with the deconvolution requires an iterative approach to solve the optimization problem, further increasing the currently heavy computation load. We then adopt a compromised solution, directly performing wavelet denoising algorithm on the differential permittivity images, to balance the need of SNR improvement and computation load. The denoising algorithm is a single-step soft-thresholding operation in the wavelet space on the images. This operation produces a solution of the following the optimization formalism [30]

$$\min_{\mathbf{x}} \frac{1}{2} \|\hat{\mathbf{x}} - \mathbf{x}\|_2^2 + \tau_{\text{w}} \|\mathbf{W}\mathbf{x}\|_1 , \tag{44}$$

where $\hat{\mathbf{x}}$ is the input image, τ_{w} is the wavelet regularization parameter, and $\mathbf{W}$ is the operator of the wavelet transform. The solution of this optimization problem is analytical and is expressed as

$$\begin{aligned} &\mathbf{x}_{\text{sol}} = \mathbf{W}^{-1}\mathrm{T}_{\tau_{\text{w}}}(\mathbf{W}\hat{\mathbf{x}}), \\ &\text{where} \quad [\mathrm{T}_{\tau_{\text{w}}}(\mathbf{a})]_i = \begin{cases} 0, & |a_i| \le \tau_{\text{w}} \\ \frac{|a_i| - \tau_{\text{w}}}{|a_i|} \cdot a_i, & |a_i| > \tau_{\text{w}} \end{cases} \end{aligned} \tag{45}$$

To denoise the differential permittivity images, τ_{w} is generally chosen to be 10% to 20% of the average signal level.

In addition to low SNR of the differential permittivity measurements, strong variations in mean permittivity can mask the true anisotropy by introducing edge birefringence. A distinguishing feature of the edge birefringence is two

maxima [31] around the edge with orthogonal orientations. The form birefringence of a true anisotropic structure (not the edge birefringence) shows a single maxima of a consistent orientation. To emphasize true anisotropy in the differential permittivity channel, we suppress edge retardance by computing an orientation consistency map. Using this map, we reject features with fast-varying orientation. We apply this additional filtering on the cardiomyocyte dataset we show in fig. 5.

The orientation continuity map is computed through the following steps. First, we compute the scattering potential tensor components and filter these components with uniform filters, $U(\vec{r})$, of kernel size N_k

(46)
$$\begin{cases} f_{1c,\text{avg}}(\vec{r}) &= \left[\sin^2\theta(\vec{r})\cos 2\omega(\vec{r})\right]\otimes U_{N_k}(\vec{r}) \\ f_{1s,\text{avg}}(\vec{r}) &= \left[\sin^2\theta(\vec{r})\sin 2\omega(\vec{r})\right]\otimes U_{N_k}(\vec{r}) \\ f_{2c,\text{avg}}(\vec{r}) &= \left[\sin 2\theta(\vec{r})\cos\omega(\vec{r})\right]\otimes U_{N_k}(\vec{r}) \\ f_{2s,\text{avg}}(\vec{r}) &= \left[\sin 2\theta(\vec{r})\sin\omega(\vec{r})\right]\otimes U_{N_k}(\vec{r}) \end{cases}.$$

Second, we use these averaged scattering potential tensor components to compute the differential permittivity according to (40). The averaged differential permittivity is higher when the orientation is more continuous along spatial dimensions and lower when the orientation is variable. The orientation continuity map is computed from the average differential permittivity normalized by its maximum. Multiplying this map with the original differential permittivity suppresses edge retardance. This method is effective when the orientation of the structures is consistent (fig. 5). Potential artifacts may occur when the orientation of the structure varies fast, e.g., lipids in fig. 4D).

4.3. **Computation of 3D orientations of axons and their cross-sections from structure tensor of phase image.** To obtain the structure tensor for analysis of axons' morphological orientation, we modify the vessel segmentation algorithm from the X-ray angiography community [32] for our data shown in fig. 4D. The algorithm first computes the structure tensor, the second-order gradient, of the mean permittivity $\phi(\vec{r})$, as

(47)
$$\mathscr{H}(\vec{r},s) = \begin{bmatrix} \partial^2_{x,s} & \partial_{y,s}\partial_{x,s} & \partial_{z,s}\partial_{x,s} \\ \partial_{x,s}\partial_{y,s} & \partial^2_{y,s} & \partial_{z,s}\partial_{y,s} \\ \partial_{x,s}\partial_{z,s} & \partial_{y,s}\partial_{z,s} & \partial^2_{z,s} \end{bmatrix}\phi(\vec{r}),$$

where s is the scale parameter and scale-dependent gradient is defined as a convolution between the scaled intensity and the derivative of a Gaussian function written as

(48)
$$\partial_{i,s}I(\vec{r}) = [sI(\vec{r})]\otimes_{\vec{r}}\left\{\partial_i\left[(2\pi s^2)^{-3/2}\cdot e^{-\|\vec{r}\|^2/2s^2}\right]\right\}.$$

Depending on the geometry of the interested feature (a shell-like, tubular-like, or blob-like structure), the eigenvalues of the structure tensor may be used in different ways to construct the segmentation map. In our case, we selected a shell-like structure and constructed the segmentation map as

(49)
$$\mathscr{V}(\vec{r}) = \begin{cases} \left(1-e^{-\mathscr{R}(\vec{r})/2a^2}\right)\cdot\left(1-e^{-\mathscr{S}(\vec{r})/2b^2}\right) \\ 0, \quad \zeta_2(\vec{r})>0 \end{cases},$$

where

(50)
$$\mathscr{R}(\vec{r}) = \frac{|\zeta_2(\vec{r})|}{\sqrt{\zeta_0(\vec{r})\cdot\zeta_1(\vec{r})}} \qquad \mathscr{S}(\vec{r}) = \sqrt{\sum_{i\le 2}\zeta_i^2(\vec{r})},$$

$|\zeta_2(\vec{r})| > |\zeta_1(\vec{r})| > |\zeta_0(\vec{r})|$ are eigenvalues of the structure tensor at position $\vec{r}$, a and b are parameters to adjust the level of sharpness of the segmentation map. The eigenvectors corresponding to the largest eigenvalue of the structure tensor on every voxel within this map contain the normal orientation of the shell-like structures. We then use these eigenvectors as a different measurement of lipids' 3D orientation to verify our results from 3D anisotropy.

5. TRANSFER FUNCTIONS AND CHOICE OF ILLUMINATION PATTERN

Our choice of the illumination patterns for our experiment is different from past literature of 3D differential phase contrast (3D DPC) microscopy [20] and 3D polarized light imaging (3D PLI) [33, 34, 35], informed by the following considerations.

To get 3D phase information, 3D DPC exploits illumination patterns of 4 rotating half-circles and the illumination NA matched with imaging NA, which is an extension from 2D DPC [17]. In 3D PLI, 3D orientation and the differential permittivity of the optically anisotropic material (axons in human brain) are extracted with circular pattern, polarization-sensitive detection, and a tilting stage. 3D PLI relies on one untilted acquisition followed by four acquisitions with tilts spaced at 90° orientations. The 4 tilted conditions with circular pattern is analogous to keeping the stage level, but illuminating the sample with 4 off-axis illuminations at 90° orientations as has been done in 3D DPC. The one untilted polarization measurement plays an important role for retrieving the 3D orientation retrieval shown in [33]. From these observations, we chose to use combination of circular illumination pattern plus several off-axis illumination patterns with a polarization-sensitive imaging path to encode 3D mean permittivity, differential permittivity, and 3D orientation information.

There are a variety of illumination patterns that could be used to encode permittivity tensor. Different illumination patterns result in different shapes of the transfer functions in (6), which affects robustness to noise at different 3D spatial frequencies. By visualizing these transfer functions, we visualize the strength with which the specimen information is transferred for chosen illumination pattern.

Extended Data Fig. 3 compares the absolute sums of the transfer functions over illumination patterns for two possible combinations of illumination patterns that are deduced from past literature. These summed transfer functions describe how individual scattering potential tensor components couple into the measured intensities as a function of their spatial frequencies. In summary, S_0 carries phase ($\ell = 0\text{r}$) and absorption information ($\ell = 0\text{i}$), while S_1 and S_2 carry anisotropy information ($\ell = 1\text{c}, 1\text{s}, 2\text{c}, 2\text{s}, 3$). For both sets of illumination patterns, phase, absorption, and projected anisotropy information ($\ell = 1\text{c}, 1\text{s}$) transfer relatively well compared to the last three components ($\ell = 2\text{c}, 2\text{s}, 3$). The last component of the scattering potential tensor, which contains information of optic sign, is the most poorly-transferred in both cases. This is why the estimation of the optic sign is the noisiest . Comparing the energy of the transfer function with these two sets of illumination, we find that the second set of illumination patterns generates more uniform transfer function for all the scattering potential components. Off-axis illumination with more orientations tends to transfer the specimen information better and, therefore, be more robust to noise across different spatial frequencies. However, larger number of off-axis illumination patterns require longer acquisition. To balance the trade-off between the noise performance and the acquisition time, we choose the second set of illumination patterns for our experiment, which consists of 8 off-axis sector patterns at the highest NA, followed by 1 on-axis circular pattern. Note that these are not the only choices for proper permittivity tensor retrieval. Other combinations of patterns are possible, but it would require further investigation to determine the optimal one as that is done in phase imaging community [36, 37].

6. THE FORMATION OF LASER-INDUCED MODIFICATIONS IN FUSED SILICA

In this note, we discuss the origin of the various density and anisotropy patterns seen in the photolithographically written anisotropic glass targets. These targets are written with the technology used to store digital information in glass, which provides the advantages of high density and long-term stability.

When an intense femtosecond laser pulse is focused into transparent material, e.g., silica glass, high-order nonlinear absorption allows the energy to be deposited predominantly within the focal volume, producing a local permanent refractive-index modification. Although the process of energy absorption is now well understood, the optical properties of the induced structures are an exciting area of research.

Depending on the level of laser intensity, one can induce any of four qualitatively different types of material modification: positive refractive-index change (type 1) at relatively low intensity; birefringent regions with the evidence

of nanograting formation (type 2) [38, 39] at intermediate intensity and voids (type 3) at high intensity. Recently new type of birefringent modification has been observed as random arrangement of prolate-shaped nanopores at transition from the type 1 to type 2 and coined as type X modification [40]. The transition threshold from one kind of structure to another depends on the laser writing parameters such as pulse duration, wavelength and irradiation time. The nanograting (type 2) modification is observed under SEM as periodic subwavelength arrangement of nanoplanes with features as small as 20 nm. The orientation of both nanogratings and elongated nanopores can be controlled by the polarization of the writing laser and results in form birefringence with slow axis oriented perpendicular to the polarization of writing laser and along the nanoplanes or long axis of prolate-shape nanopores. Thus, the optic sign of the induced birefringence is negative with the birefringence for nanograting modification close to the birefringence of the quartz crystal, and the birefringence of nanopore modification is about 4 times lower.

Birefringent structures produced with nanograting modification reveal ultra- low scattering loss and were observed when writing with infrared 1030 nm laser wavelength. Writing with shorter wavelength, such as 515 nm is advantageous for increasing the spatial resolution. With a commercial quantitative polarization microscope, Abrio, only nanograting structures were revealed, since Abrio can only measure the average change of birefringence along axial direction. The structure of imprinted birefringent modification, e. g. the possible coexistence of different types of modification along the axial direction shown in fig. 3C, in principle can be characterized by destructive SEM. However non-destructive method of 3D birefringence imaging, such as PTI, is more convenient and resulted in the observation of two distinct modifications across the depth. This data shows that PTI can be used for reading 3D information storged in these glass storage devices.

7. Cytopathic effects of RSV infection on A549 cells

In this note, we use PTI multiplexed with fluorescence deconvolution microscopy to analyze cytopathic effects of another model of infection: A549 cells infected by respiratory syncytial virus (RSV). RSV is a leading cause of serious lower respiratory tract infection in infants and elderly [41]. Although it is known that RSV replicates in cytoplasm [42], many aspects of its impact on cellular architecture remain to be studied. As a lung cell line, A549 cells are commonly used to study RSV infection [43]. Many studies have immunostained A549 cells to investigate the organelle rearrangement [44, 45] during RSV infection. Quantitative measurements of physical properties of RSV-infected cells can clarify the cytopathic effects and enable image-based staging of the infection cycle.

Figure 2A and the corresponding through-focus video Supplementary Video 11 show images of fixed A549 cells with the label-free channels (mean permittivity, differential permittivity, in-plane orientation, out-of-plane tilt, and optic sign probability) and the fluorescence channels. PT measurements reported in this result are acquired with an 1.2 NA (NA_c) oil immersion condenser and 1.2NA (NA_o) water immersion objective. The mean permittivity shows nuclei, nucleoli, and a mesh of membranous structures surrounding nuclei. The differential permittivity identifies anisotropic fibers around the nucleus and the membranous structures near the the cell-cell junctions. One may interpret birefringence at cell-cell junctions to be simply edge birefringence. However, these structures have high differential permittivity relative to the mean permittivity and are not present at every edge of the cells. Therefore, we think that these structures are true anisotropic structures. The A549 cells are stained with DAPI (blue) to label DNA and GFP to label RSV. We see co-localization of density, anisotropy, and fluorescence of nuclei. Since these are the uninfected A549 cells, we observe no GFP signal.

Figure 2B and 2C zoom in on two anisotropic structures indicated by the blue box and the orange box in Figure 2A. Figure 2B shows that the anisotropic structure is positive uniaxial and the 3D orientation of the symmetry axis of PT is aligned with the symmetry axis of the structure throughout the volume. The alignment of the symmetry axes of the microscale PT and the mesoscale structure suggests that this is a polymeric fiber assembly with the bound electrons more easily polarized in the fiber axis, analogous to myofibrils in sarcomeres. From the projected 2D orientation in the side view, we find that the fiber is slightly inclined out of the focal plane. Figure 2C shows the orthogonal sections of the anisotropic structure at the cell-cell junction along its tangential and normal directions. The orthogonal sections suggest this positive uniaxial structure has negative phase and almost $3\times$ stronger differential permittivity than the fibers around the nucleus. From the projected 2D orientation in both views, we see the anisotropy is perpendicular to the symmetry axis. This orthogonality between the symmetry axes of the microscale PT and mesoscale structure suggests this structure is a membrane structure with the bound electrons more polarized perpendicular to the sheet, analogous to lipid bi-layers in the myelin sheaths. A549 cells are epithelial cells that form tight junctions between cells when the confluence of cells is high, which can align the membranes of neighboring cells to give rise to high anisotropy.

Now we compare above phenotypes with the phenotypes of the RSV infected A549 cells in Figure 3A and corresponding through-focus videos (Supplementary Video 12). In the fluorescence image, GFP distribution is visible in few cells, indicating they are infected. We observed three consistent phenotypes across multiple cells. First, the highly anisotropic membranes at the cell-cell junctions that are visible in the uninfected cells disappear in the infected cells, suggesting the reorganization of the cellular functions due to RSV infection suppresses formation of these membranes. Second, the perinuclear fibers seen in the uninfected cells are still visible in zoom-in fields of view shown in Figure 3B. Third, the phase or electron density of nuclei and nucleoli in the infected A549 cells are generally larger than the phase of the uninfected A549 cells. Using the DAPI images, we conduct a segmentation of the nuclei for both uninfected and infected A549 cells. Figure 3C shows the histogram of the average and standard deviation of phase value in the nuclei. In the infection condition, we observe noticeably higher average and standard deviation of phase in the nuclei. This suggests that the infection causes the cells to condense themselves. The optic sign probability estimation of the infected condition is noisier than the estimation of the uninfected condition because the cells in the infection condition are thicker and may introduce more unwanted scattering artifacts. Further, we find that phenotypic changes observed

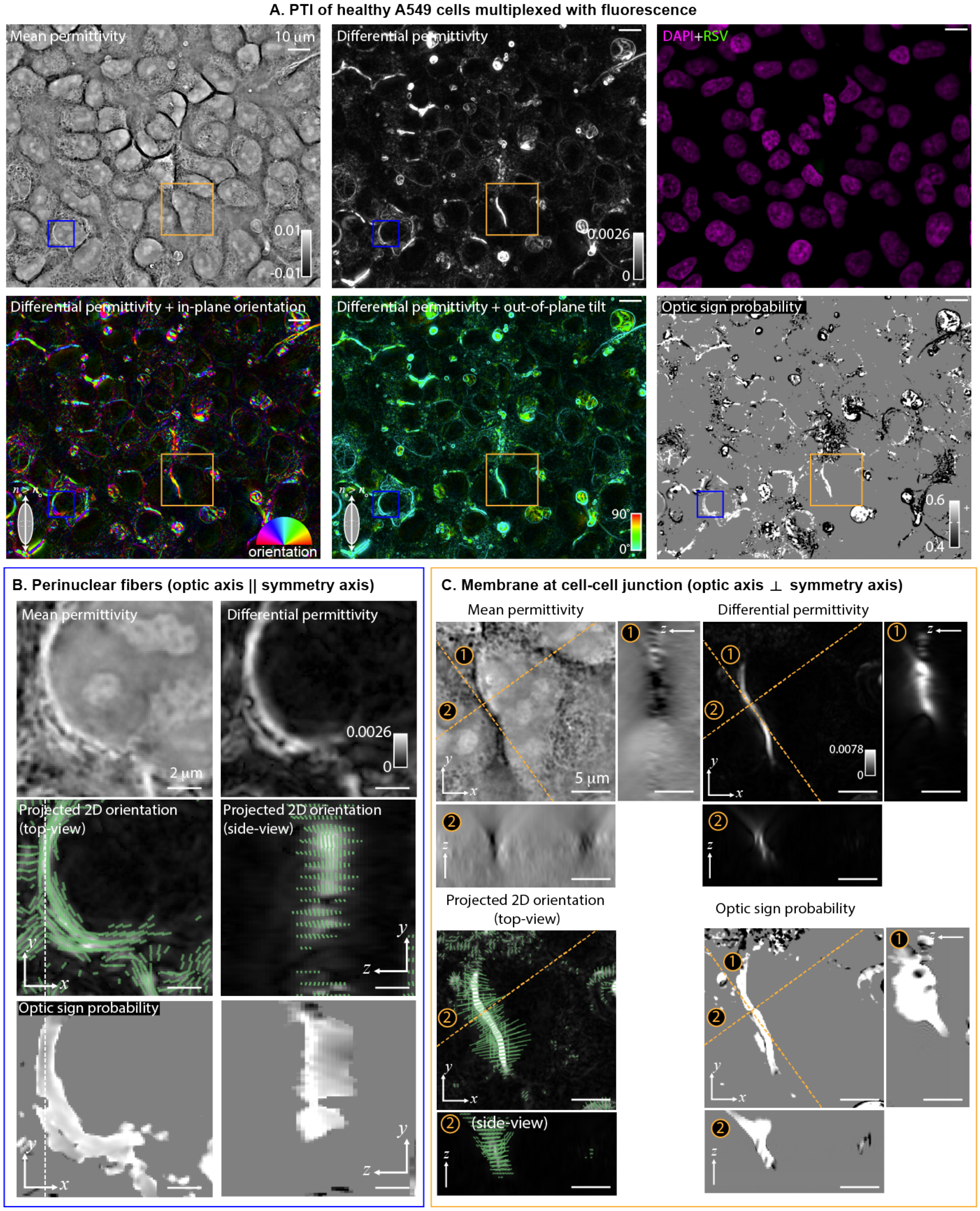

FIGURE 2. **Imaging physical and molecular features in healthy A549 cells:** **(A)** 3D mean permittivity, 3D differential permittivity, fluorescence, in-plane orientation, out-of-plane tilt, and optic sign probability images of the healthy A549 cells. The fluorescence overlay shows DAPI stain in magenta and RSV-GFP signal in green. **(B)** *xy* slice of mean permittivity and differential permittivity from the volume in blue-square region in (A) shows dense, anisotropic perinuclear structures. We show orthogonal slices (*x*-*y*,*y*-*z*) of differential permittivity, optic sign probability, and orientation projected onto the plane of viewing. **(C)** The orthogonal slices of 3D mean permittivity, 3D differential permittivity, projected orientation, and optic sign probability over the field of view indicated by the orange box in (A) show highly anisotropic structures at the cell-cell junction with a positive optic sign. The orange dashed lines cut at two axes (labeled with ① and ②) tangent and normal to the structure to show its cross-sections.

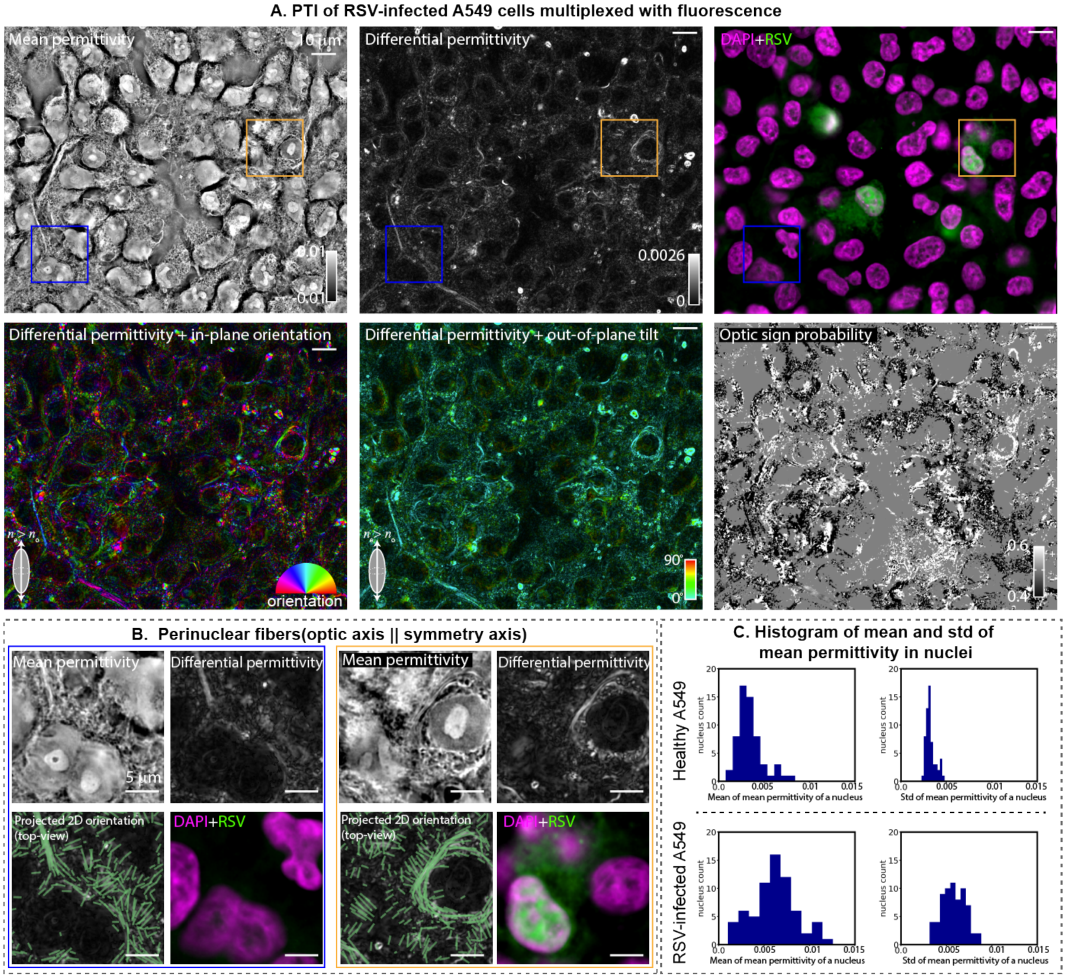

FIGURE 3. **Imaging physical and molecular changes in A549 cells due to infection by respiratory syncytial virus (RSV): (A)** 3D mean permittivity, 3D differential permittivity, fluorescence, in-plane orientation, out-of-plane tilt, and optic sign probability images of the A549 cells infected with RSV. **(B)** Two fields of view chosen from (A) show the perinuclear fibers observed in Figure 2 with label-free channels. We do not observe the highly anisotropic membranes at cell-cell junctions in the infected condition. **(C)** Histogram of mean and standard deviation of mean permittivity of about 60 nuclei for the uninfected and infected A549 cells show significant increase in density and variability in density of nuclei in infected cells. These statistics agree with the images of mean permittivity shown in Figure 2, and this figure.

in label-free images are present across the field of view, even when the infected cells are only a small fraction of the cell population. This suggests that the infected cells modulate the architecture and activity of neighboring cells through paracrine signaling.

References

[1] Yaghjian, A. D. *A Direct Approach to the Derivation of Electric Dyadic Green's Functions* (Nat. Bur. Stand. (U.S.), Tech. Note 1000, 1978).

[2] Paknys, R. in *Applied Frequency-Domain Electromagnetics* 335–354 (John Wiley & Sons, Ltd, 2016).

[3] Emil Wolf. Unified theory of coherence and polarization of random electromagnetic beams. *Physics Letters A* **312**, 263–267 (2003) .

[4] Korotkova, O. & Wolf, E. Generalized Stokes parameters of random electromagnetic beams. *Optics Letters* **30** (2), 198 (2005). doi:10.1364/OL.30.000198 .

[5] Joseph Goodman. *Statistical Optics* (Wiley, 2015).

[6] Mehta, S. B. & Oldenbourg, R. Image simulation for biological microscopy: Microlith. *Biomedical Optics Express* **5** (6), 1822–1838 (2014) .

[7] Born, M. & Wolf, E. *Principles of Optics: Electromagnetic Theory of Propagation, Interference and Diffraction of Light* (Elsevier, 2013).

[8] Feynman, R. P., Leighton, R. B. & Sands, M. *The Feynman Lectures on Physics, Vol. II* (Basic Books, 2011).

[9] Kong, J. A. Optics of bianisotropic media*. *Journal of the Optical Society of America* **64** (10), 1304 (1974). doi:10.1364/JOSA.64.001304 .

[10] Wolf, E. Three-dimensional structure determination of semi-transparent objects from holographic data. *Optics Communications* **1** (4), 153–156 (1969). doi:10.1016/0030-4018(69)90052-2 .

[11] Loh, Y. L. The Ewald sphere construction for radiation, scattering, and diffraction. *American Journal of Physics* **85** (4), 277–288 (2017). doi:10.1119/1.4973369 .

[12] Sheppard, C. J. R., Kou, S. S. & Lin, J. The Green-function transform and wave propagation. *Frontiers in Physics* **2** (2014) .

[13] Marathay, A. S. Fourier transform of the Green's function for the Helmholtz equation. *Journal of the Optical Society of America* **65** (8), 964 (1975). doi:10.1364/JOSA.65.000964 .

[14] Kamilov, U. S., Liu, D., Mansour, H. & Boufounos, P. T. A Recursive Born Approach to Nonlinear Inverse Scattering. *IEEE Signal Processing Letters* **23** (8), 1052–1056 (2016). doi:10.1109/LSP.2016.2579647 .

[15] Liu, H.-Y. *et al.* SEAGLE: Sparsity-Driven Image Reconstruction Under Multiple Scattering. *IEEE Transactions on Computational Imaging* **4** (1), 73–86 (2018). doi:10.1109/TCI.2017.2764461 .

[16] Streibl, N. Three-dimensional imaging by a microscope. *JOSA A* **2** (2), 121–127 (1985). doi:10.1364/JOSAA.2.000121 .

[17] Tian, L. & Waller, L. Quantitative differential phase contrast imaging in an LED array microscope. *Optics Express* **23** (9), 11394–11403 (2015). doi:10.1364/OE.23.011394 .

[18] Jenkins, M. H. & Gaylord, T. K. Quantitative phase microscopy via optimized inversion of the phase optical transfer function. *Applied Optics* **54** (28), 8566 (2015). doi:10.1364/AO.54.008566 .

[19] Claus, R. A., Naulleau, P. P., Neureuther, A. R. & Waller, L. Quantitative phase retrieval with arbitrary pupil and illumination. *Optics Express* **23** (20), 26672–26682 (2015). doi:10.1364/OE.23.026672 .

[20] Chen, M., Tian, L. & Waller, L. 3D differential phase contrast microscopy. *Biomedical Optics Express* **7** (10), 3940–3950 (2016). doi:10.1364/BOE.7.003940 .

[21] Foreman, M. R. & Török, P. Computational methods in vectorial imaging. *Journal of Modern Optics* **58** (5-6), 339–364 (2011). doi:10.1080/09500340.2010.525668 .

[22] Guo, S.-M. *et al.* Revealing architectural order with quantitative label-free imaging and deep learning. *eLife* **9**, e55502 (2020). doi:10.7554/eLife.55502 .

[23] Ivanov, I. E. *et al.* Correlative imaging of the spatio-angular dynamics of biological systems with multimodal instant polarization microscope. *Biomedical Optics Express* **13** (5), 3102–3119 (2022). doi:10.1364/BOE.455770 .

[24] Fienup, J. R. Phase retrieval algorithms: A comparison. *Applied Optics* **21** (15), 2758–2769 (1982). doi:10.1364/AO.21.002758 .

[25] Yeh, L.-H. *et al.* Experimental robustness of Fourier ptychography phase retrieval algorithms. *Optics Express* **23** (26), 33214–33240 (2015). doi:10.1364/OE.23.033214 .

[26] Mehta, S. B. & Sheppard, C. J. R. Partially coherent image formation in differential interference contrast (DIC) microscope. *Optics Express* **16** (24), 19462–19479 (2008). doi:10.1364/OE.16.019462 .

[27] Chipman, R. A., Lam, W. S. T. & Young, G. *Polarized Light and Optical Systems* 1st edition edn (CRC Press, Boca Raton, 2018).

[28] Azzam, R. M. A. & Lopez, A. G. Accurate calibration of the four-detector photopolarimeter with imperfect polarizing optical elements. *Journal of the Optical Society of America A* **6** (10), 1513 (1989). doi:10.1364/JOSAA.6.001513 .

[29] Mehta, S. B. *et al.* Dissection of molecular assembly dynamics by tracking orientation and position of single molecules in live cells. *Proceedings of the National Academy of Sciences* **113** (42), E6352–E6361 (2016). doi:10.1073/pnas.1607674113 .

[30] Parikh, N. & Boyd, S. Proximal Algorithms. *Foundations and Trends in Optimization* **1** (3), 127–239 (2014). doi:10.1561/2400000003 .

[31] Oldenbourg, R. Analysis of edge birefringence. *Biophysical Journal* **60** (3), 629–641 (1991). doi:10.1016/S0006-3495(91)82092-6 .

[32] Frangi, A. F., Niessen, W. J., Vincken, K. L. & Viergever, M. A. *Multiscale vessel enhancement filtering*, 130–137 (Springer-Verlag, 1998).

[33] Wiese, H. *Enhancing the Signal Interpretation and Microscopical Hardware Concept of 3D Polarized Light Imaging*. Wissenschaftliche Abschlussarbeiten » Dissertation, Universität Wuppertal, Fakultät für Mathematik und Naturwissenschaften » Physik » Dissertationen (2018).

[34] Schmitz, D. *et al.* Derivation of fiber orientations from oblique views through human brain sections in 3D-polarized light imaging. *Frontiers in Neuroanatomy* **12** (2018). doi:10.3389/fnana.2018.00075 .

[35] Schmitz, D., Amunts, K., Lippert, T. & Axer, M. *A least squares approach for the reconstruction of nerve fiber orientations from tiltable specimen experiments in 3D-PLI*, 132–135 (2018).

[36] Kellman, M. R., Bostan, E., Repina, N. A. & Waller, L. Physics-Based Learned Design: Optimized Coded-Illumination for Quantitative Phase Imaging. *IEEE Transactions on Computational Imaging* **5** (3), 344–353 (2019). doi:10.1109/TCI.2019.2905434 .

[37] Fan, Y. *et al.* Optimal illumination scheme for isotropic quantitative differential phase contrast microscopy. *Photonics Research* **7** (8), 890–904 (2019). doi:10.1364/PRJ.7.000890 .

[38] Shimotsuma, Y., Kazansky, P. G., Qiu, J. & Hirao, K. Self-Organized Nanogratings in Glass Irradiated by Ultrashort Light Pulses. *Physical Review Letters* **91** (24), 247405 (2003). doi:10.1103/PhysRevLett.91.247405 .

[39] Bricchi, E., Klappauf, B. G. & Kazansky, P. G. Form birefringence and negative index change created by femtosecond direct writing in transparent materials. *Optics Letters* **29** (1), 119–121 (2004). doi:10.1364/OL.29.000119 .

[40] Sakakura, M., Lei, Y., Wang, L., Yu, Y.-H. & Kazansky, P. G. Ultralow-loss geometric phase and polarization shaping by ultrafast laser writing in silica glass. *Light: Science & Applications* **9** (1), 15 (2020). doi:10.1038/s41377-020-0250-y .

[41] Hall, C. B. *et al.* The Burden of Respiratory Syncytial Virus Infection in Young Children. *New England Journal of Medicine* **360** (6), 588–598 (2009). doi:10.1056/NEJMoa0804877 .

[42] Collins, P. L., Fearns, R. & Graham, B. S. in *Respiratory Syncytial Virus: Virology, Reverse Genetics, and Pathogenesis of Disease* (eds Anderson, L. J. & Graham, B. S.) *Challenges and Opportunities for Respiratory Syncytial Virus Vaccines* Current Topics in Microbiology and Immunology, 3–38 (Springer Berlin Heidelberg, Berlin, Heidelberg, 2013).

[43] Hillyer, P. *et al.* Differential Responses by Human Respiratory Epithelial Cell Lines to Respiratory Syncytial Virus Reflect Distinct Patterns of Infection Control. *Journal of Virology* **92** (15) (2018). doi:10.1128/JVI.02202-17 .

[44] Singh, D., McCann, K. L. & Imani, F. MAPK and heat shock protein 27 activation are associated with respiratory syncytial virus induction of human bronchial epithelial monolayer disruption. *American Journal of Physiology-Lung Cellular and Molecular Physiology* **293** (2), L436–L445 (2007). doi:10.1152/ajplung.00097.2007 .

[45] Hu, M., Li, H.-M., Bogoyevitch, M. A. & Jans, D. A. Mitochondrial protein p32/HAPB1/gC1qR/C1qbp is required for efficient respiratory syncytial virus production. *Biochemical and Biophysical Research Communications* **489** (4), 460–465 (2017). doi:10.1016/j.bbrc.2017.05.171 .

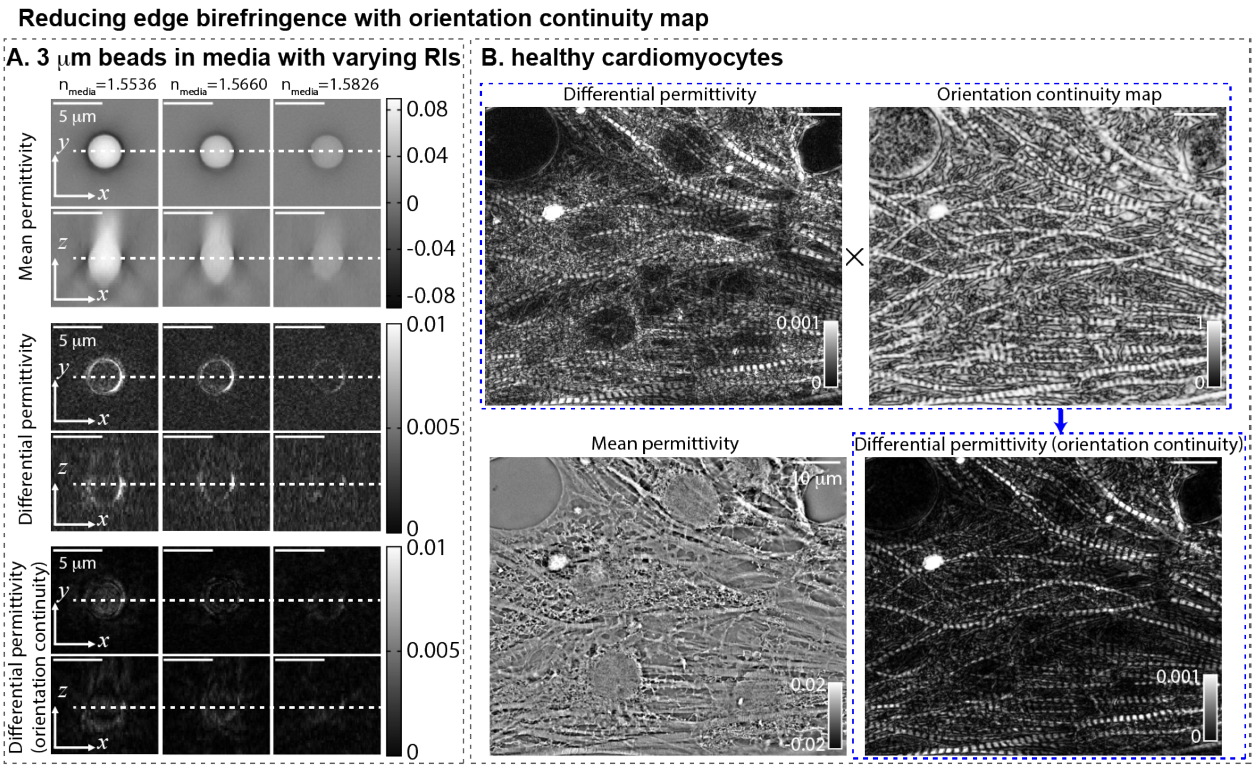

SUPPLEMENTARY FIG. 1. **Emphasizing form birefringence and suppressing edge birefringence using orientation continuity:** 3D mean permittivity, 3D differential permittivity, and 3D differential permittivity filtered with the orientation continuity map of **(A)** 3 μm beads immersed in oils with varying refractive indices and **(B)** the healthy cardiomyocytes. Orientation continuity map of the cardiomyocytes computed through (46) is shown in (B) to demonstrate how it suppresses the edge birefringence. This approach is effective for suppressing edge birefringence and detailed in Supplementary Note 4.2.

SUPPLEMENTARY FIGURES

VIDEOS

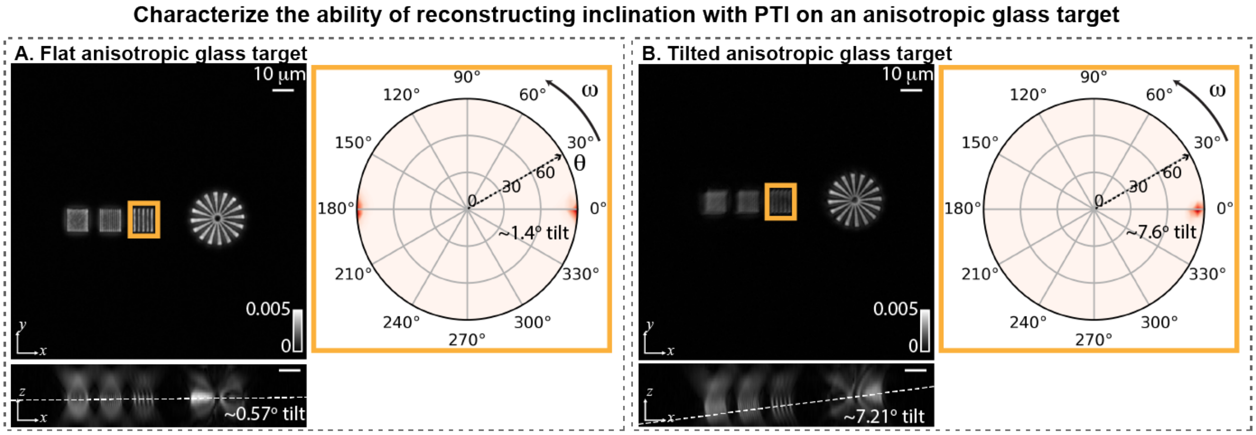

SUPPLEMENTARY FIG. 2. **Experimental verification of the reconstructed tilt angle using a test target:** We imaged **(A)** a flat and **(B)** a tilted anisotropic glass targets with 20x objective. *x*-*y* and *x*-*z* sections of 3D differential permittivity and the 3D orientation histogram of the orange ROI are shown. The *x*-*z* sections of both targets show that the tilt angles are measurable geometrically. As seen from 3D orientation histograms, the 3D orientation measured with PTI reconstruction matches well with the experimental tilt.

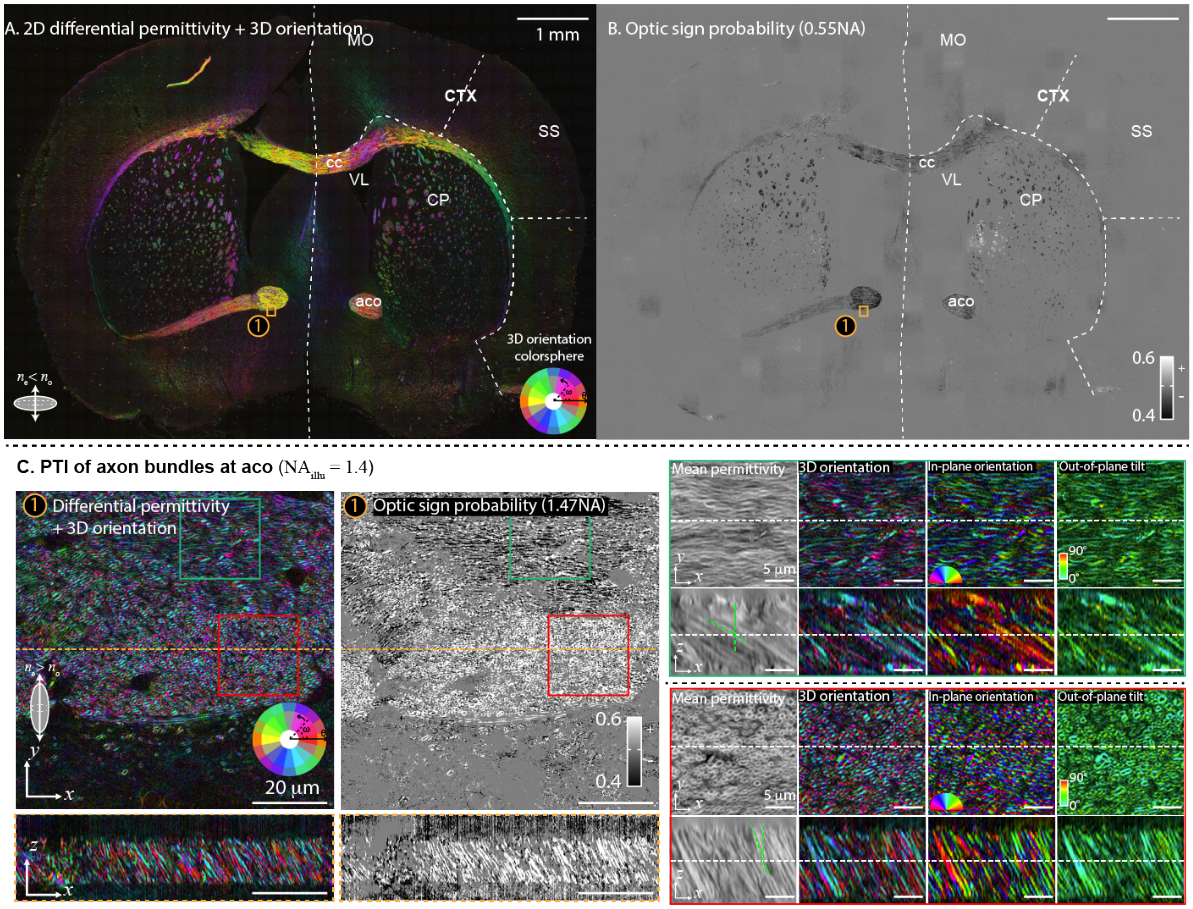

SUPPLEMENTARY FIG. 3. **3D orientation and optic sign probability of an adult mouse brain section in 2D and in 3D: (A)** 2D differential permittivity, 3D orientation and **(B)** optic sign probability images of the whole brain section show key anatomical landmarks. 3D orientation is encoded using a colorsphere (Extended Data Fig. 2). At the imaging and illumination NA of 0.55 (spatial resolution of $\sim 0.5 \times 0.5 \times 3.2$ μm), axons behave like negative uniaxial material as seen in the optic sign probability. Therefore, we assume the negative uniaxial material when displaying 3D orientation over the whole slide. Multiple anatomical landmarks are labeled according to the coronal section at level 51 of the Allen brain reference atlas, aco: anterior commissure (olfactory limb), cc: corpus callosum, CP: caudoputamen, CTX: cortex, MO: motor cortex, SS: somatosensory area, VL: ventricle. **(C, left)** We selected aco area marked with orange boxes labeled with ① in (A) and (B) for 3D high-resolution imaging with imaging NA of 1.47 and illumination NA of 1.4, i.e., with the spatial resolution of $\sim 0.23 \times 0.23 \times 0.8$ μm. The orthogonal sections of 3D differential permittivity, 3D orientation, and the optic sign probability show the complex 3D axon network. **(C, right)** We further zoom into two volumes with distinct tilt angles: green (axons tilted 60° to the left from z-axis) and red (axons tilted 20° to the left from z-axis) boxes. 3D mean permittivity and 3D orientation resolve the boundaries of individual axons, which behave as the positive uniaxial material with 3D orientation perpendicular to the surface of the membrane. The orientation is now rendered using 3D colorsphere and using two distinct colormaps that show the in-plane orientation and out-of-plane tilt to better visualize axon networks.

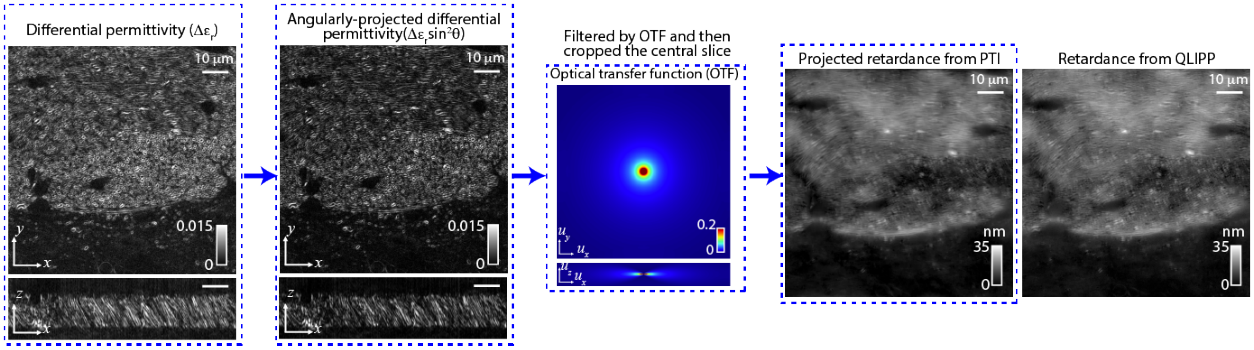

SUPPLEMENTARY FIG. 4. **Comparison of projected retardance computed from PTI and retardance measured with QLIPP:** Above data shows that the projected retardance computed by the angular projection and spatially filtering the PTI differential permittivity matches well with the retardance measurement with QLIPP. The spatial filter is the optical transfer function of the QLIPP.

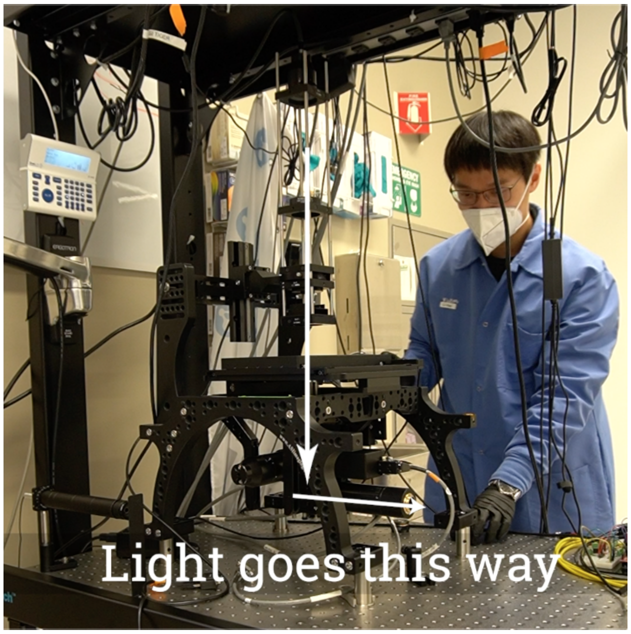

SUPPLEMENTARY VIDEO 1. The build video demonstrating how to setup PTI on a working microscope.

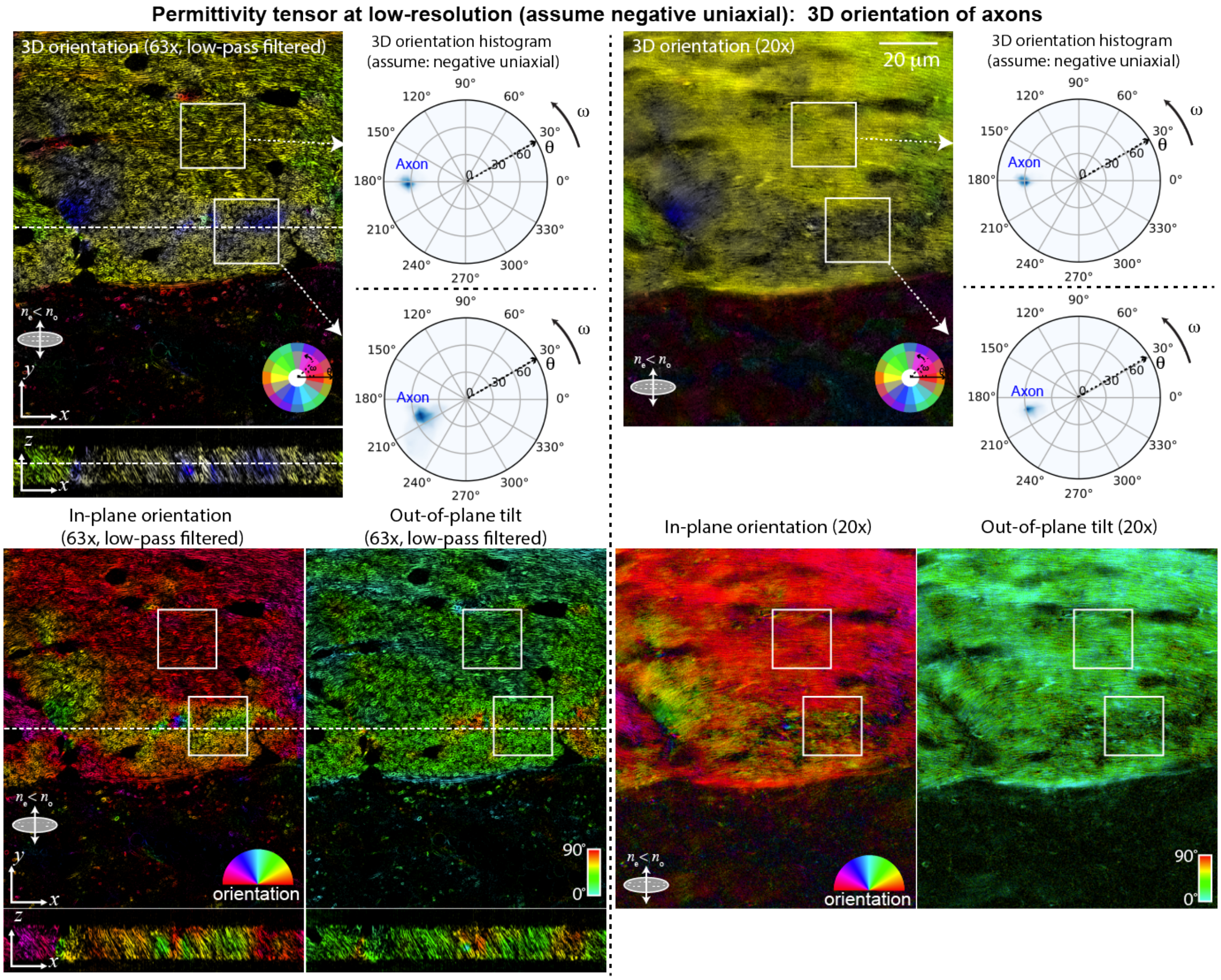

SUPPLEMENTARY FIG. 5. **Interpretation of 3D orientation measurements at low resolution:** The 3D orientation measurements (rendered in a single image with the introduced colorsphere in Extended Data Fig. 2 and in two separate images, in-plane orientation and out-of-plane tilt) of two axon bundles (the same region as shown in fig. 4D①) computed from (left) the low-pass filtered scattering potential tensor acquired at 63× objective (1.47 NA) and (right) the scattering potential tensor acquired at 20× objective (0.55NA) assuming the specimen is negative uniaxial. The averaged orientation measurements acquired at 63× show similar distribution of 3D orientation to the distribution measured at 20×, indicating that PTI provides sensitive measurement of 3D anisotropy across different spatial scales.

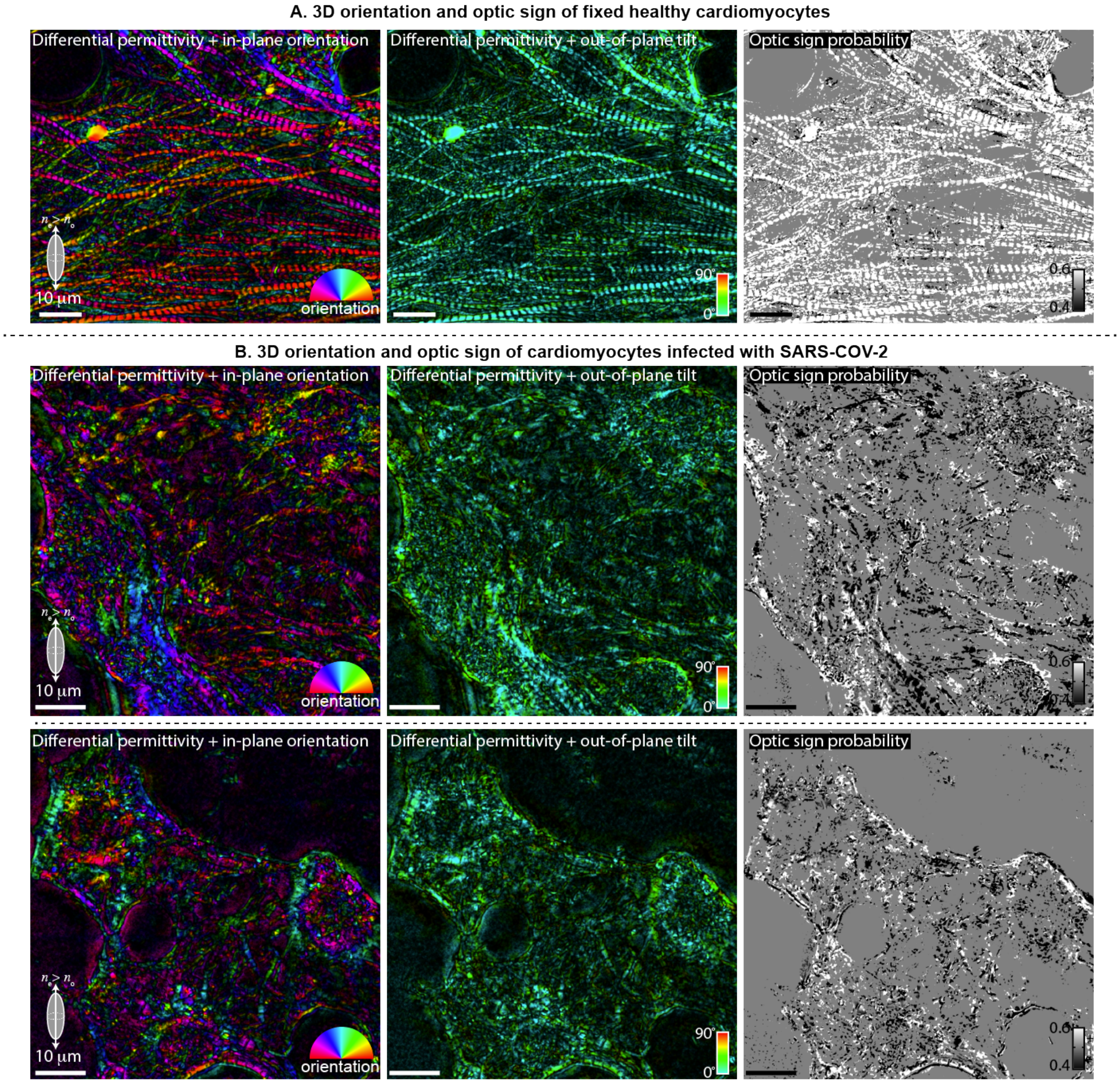

SUPPLEMENTARY FIG. 6. **3D orientation and optic sign probability of the iPSC-derived cardiomyocytes:** The 3D orientation (azimuth and tilt are rendered separately) and optic sign probability of the **(A)** uninfected and **(B)** SARS-COV-2 infected cardiomyocytes shown in fig. 5. In the uninfected cardiomyocytes, the optic sign of the sarcomere structure is found to be a positive and 3D orientation is found to point along the axis of the sarcomere. In the infected cardiomyocytes, the differential permittivity drops as seen in fig. 5D, which makes the optic sign probability and 3D orientation channels noisier. The 3D orientation maps in the infected cells show small stretches of consistent orientation, which are recognized as broken sarcomeres.

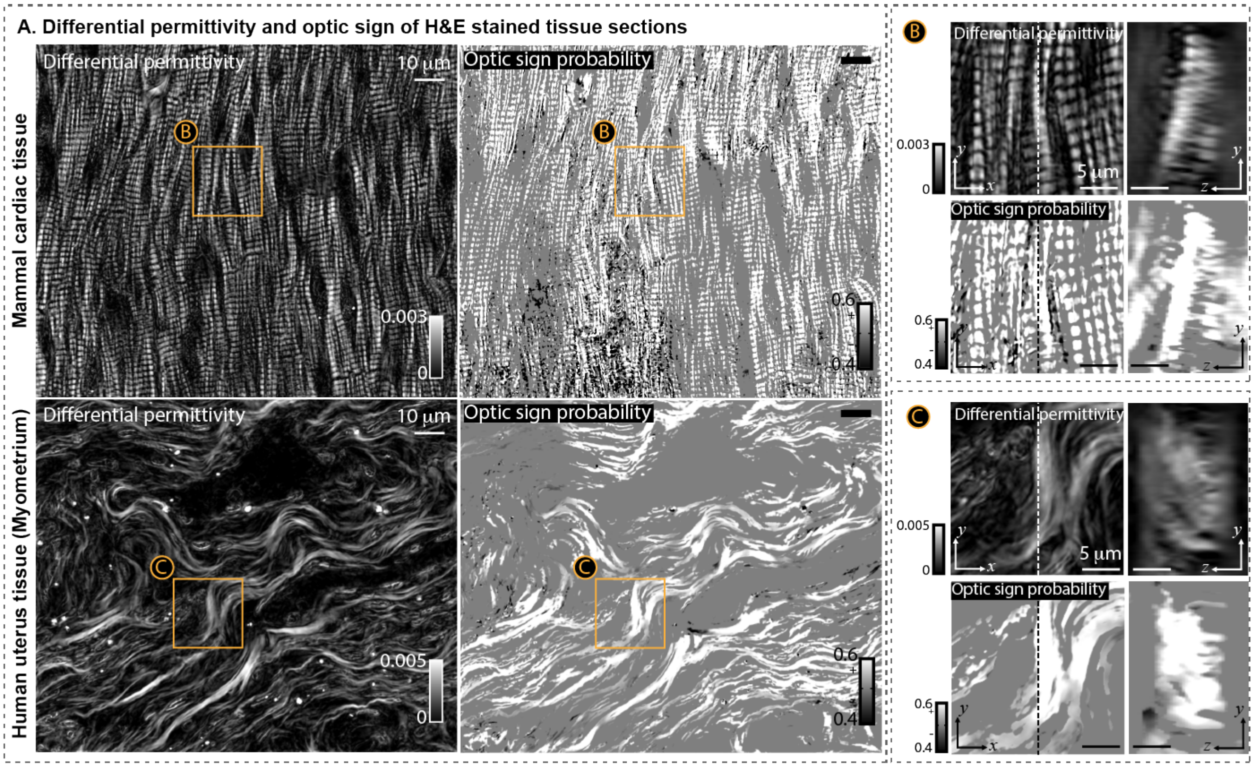

SUPPLEMENTARY FIG. 7. **3D differential permittivity and optic sign probability of the histological sections: (A)** 3D differential permittivity and optic sign probability of (top) the mammal cardiac tissue and (bottom) the myometrium region of the human uterus tissue. **(B)** The orthogonal slices (x-y and y-z) of 3D differential permittivity and optic sign probability of the field of view indicated by the orange box in the cardiac tissue. **(C)** Same information as in (B) is shown for the field of view indicated by the orange box in the uterus tissue. Highly anisotropic structures such as A-bands of sarcomeres in the cardiac muscle tissue and collagen fibers in the uterus tissue show consistent positive optic sign estimation through out the volumes.

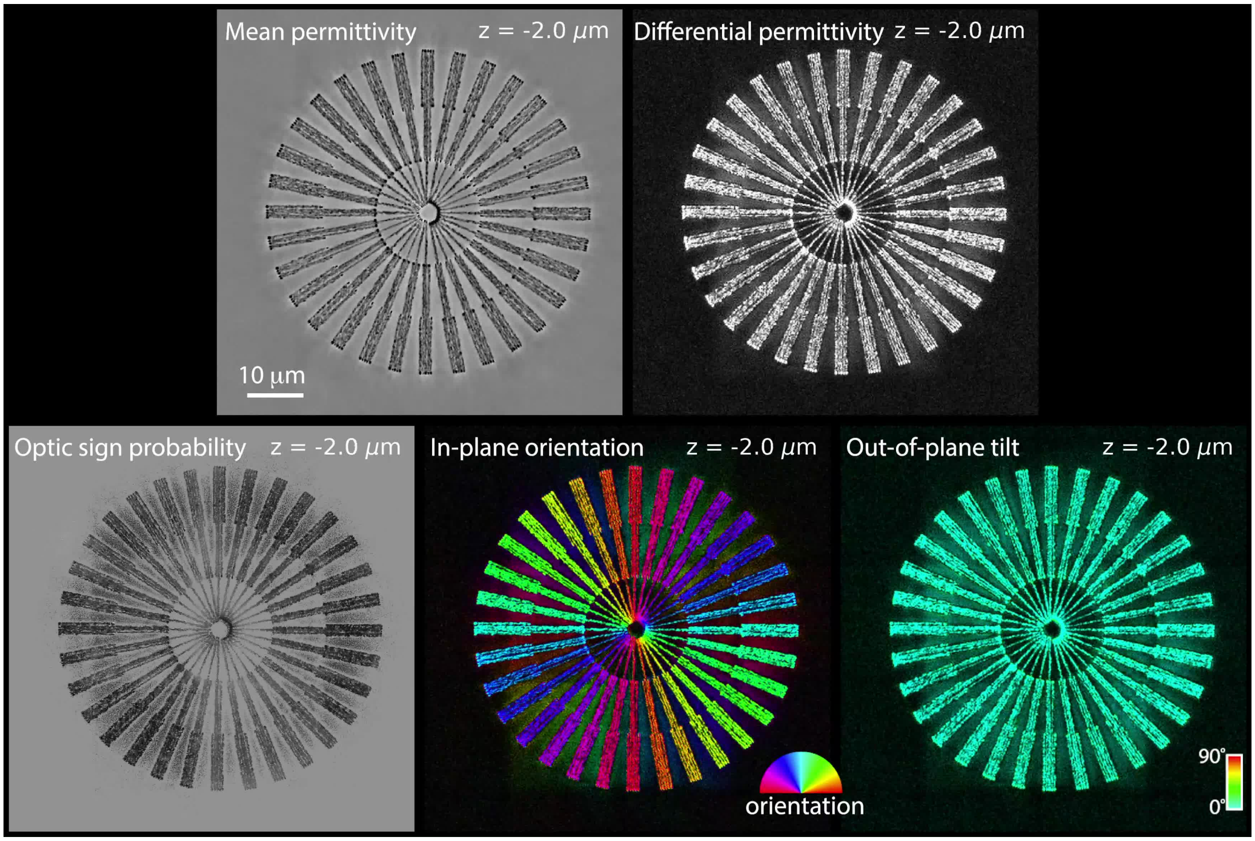

SUPPLEMENTARY VIDEO 2. z-stacks of 3D mean permittivity, 3D differential permittivity, 3D orientation (rendered separately in in-plane orientation and out-of-plane tilt) and optic sign probability images of the laser written anisotropic target shown in fig. 3.

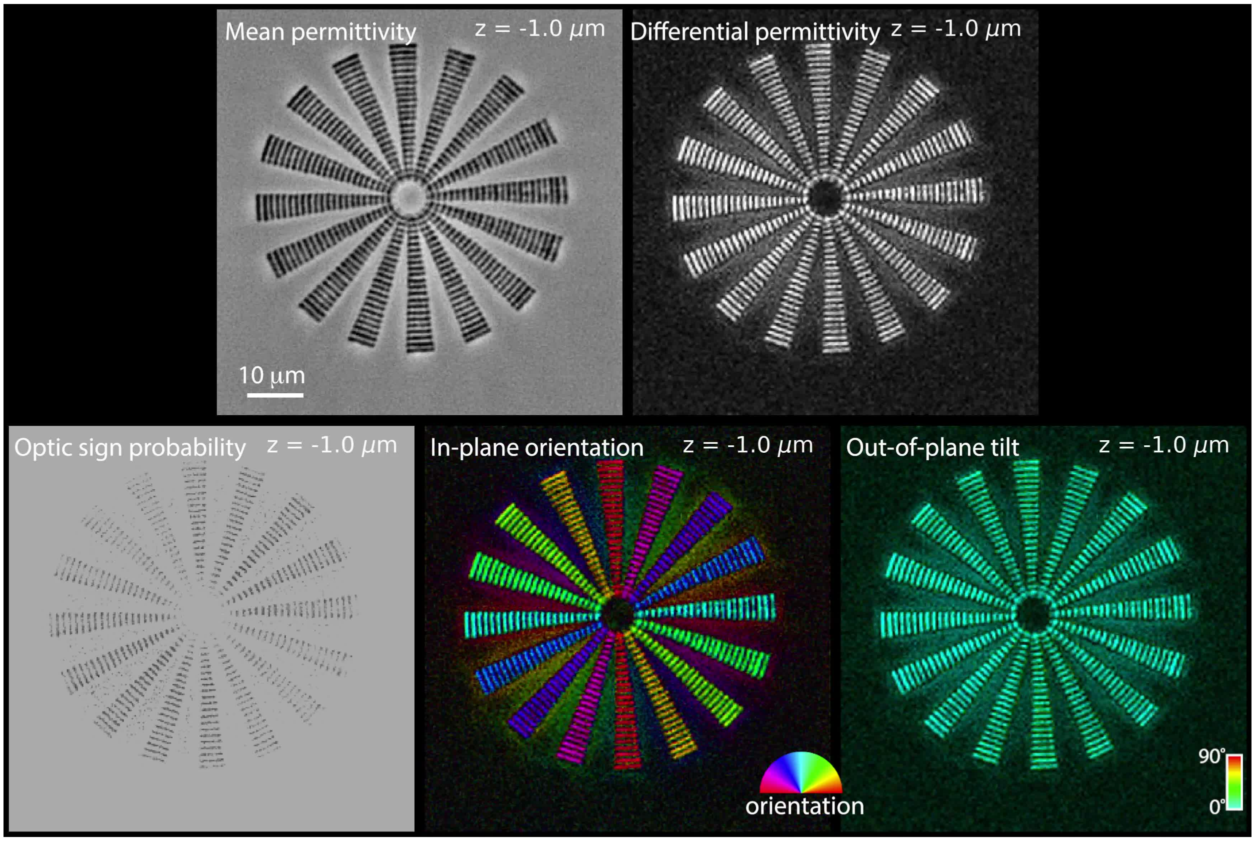

SUPPLEMENTARY VIDEO 3. z-stacks of 3D mean permittivity, 3D differential permittivity, 3D orientation (rendered separately in in-plane orientation and out-of-plane tilt) and optic sign probability images of the laser written anisotropic target shown in Extended Data Fig. 5.

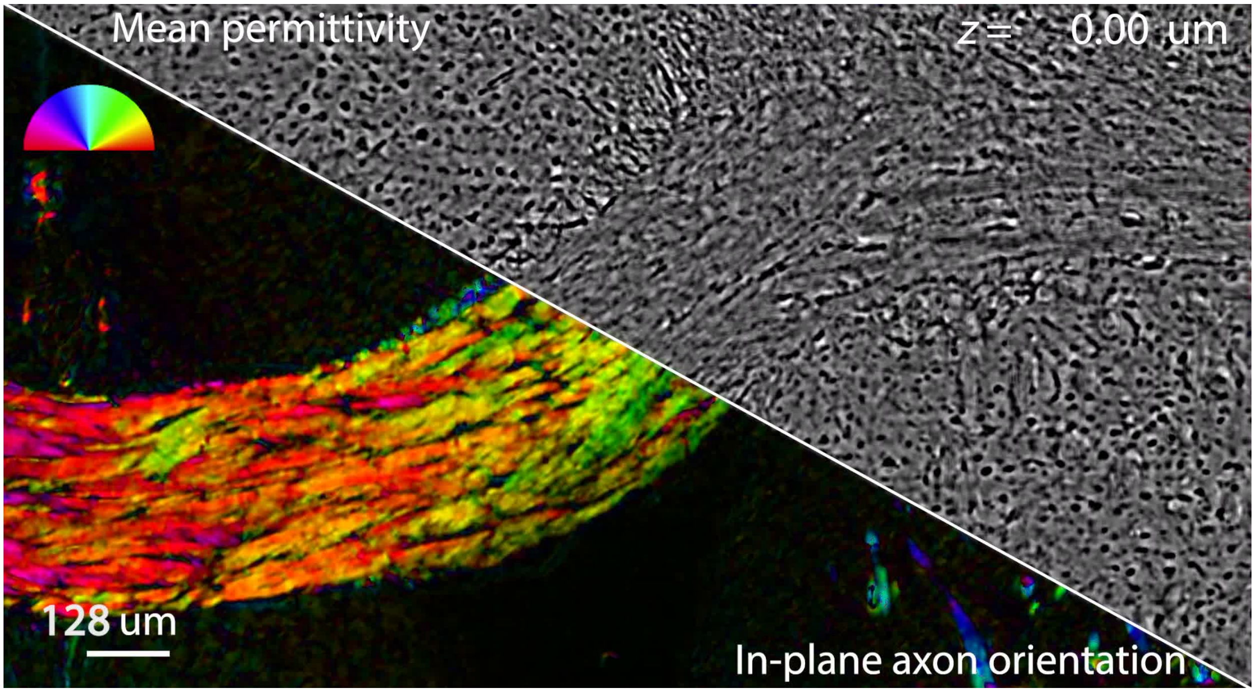

SUPPLEMENTARY VIDEO 4. Visualization of high-resolution (1.47NA) 3D mean permittivity and 3D differential permittivity of the adult mouse brain section.

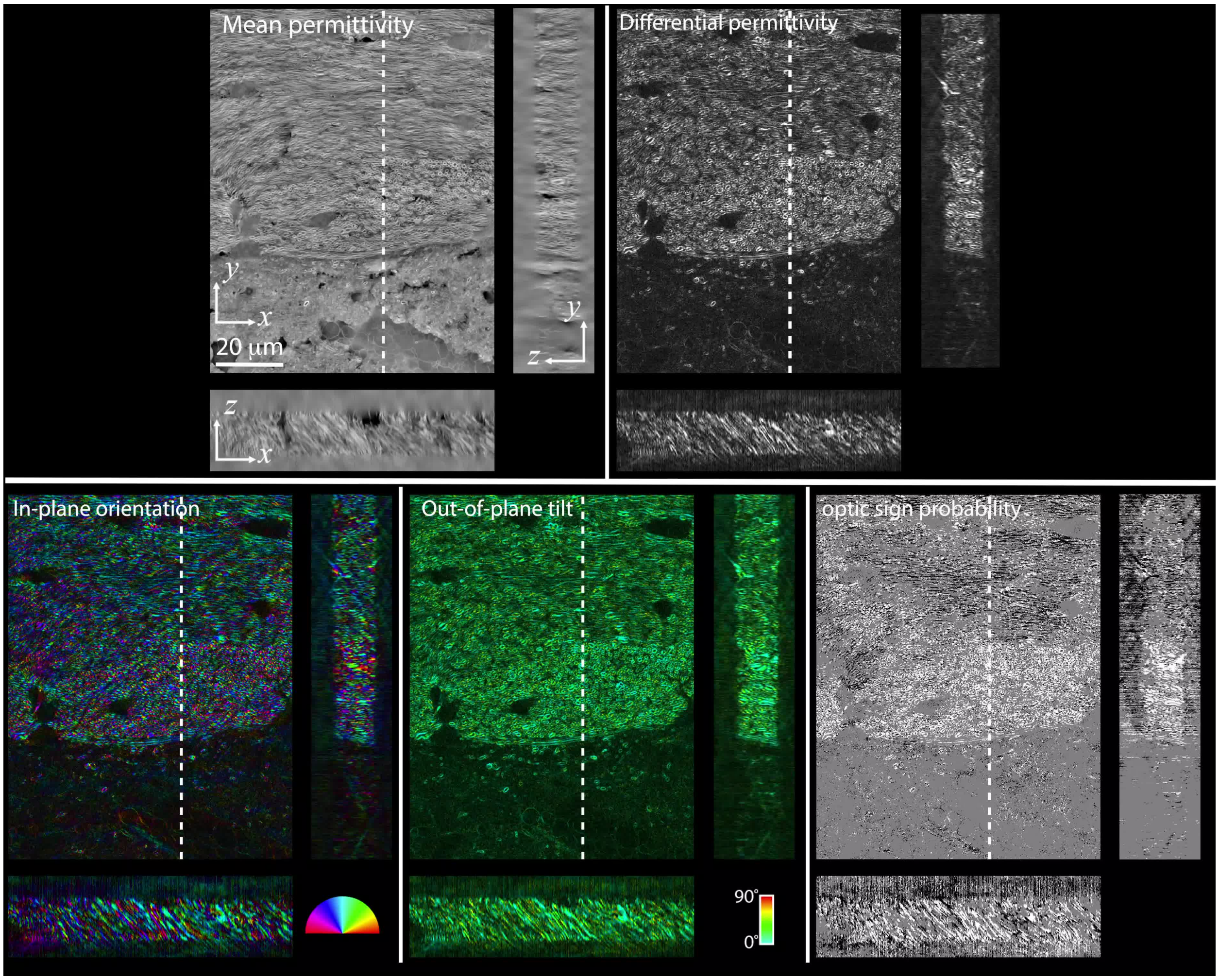

SUPPLEMENTARY VIDEO 5. z-stacks, x-z section, and y-z section of 3D mean permittivity, 3D differential permittivity, 3D orientation (rendered separately in in-plane orientation and out-of-plane tilt) and optic sign probability images of field of view ① of the mouse brain section shown in fig. 4.

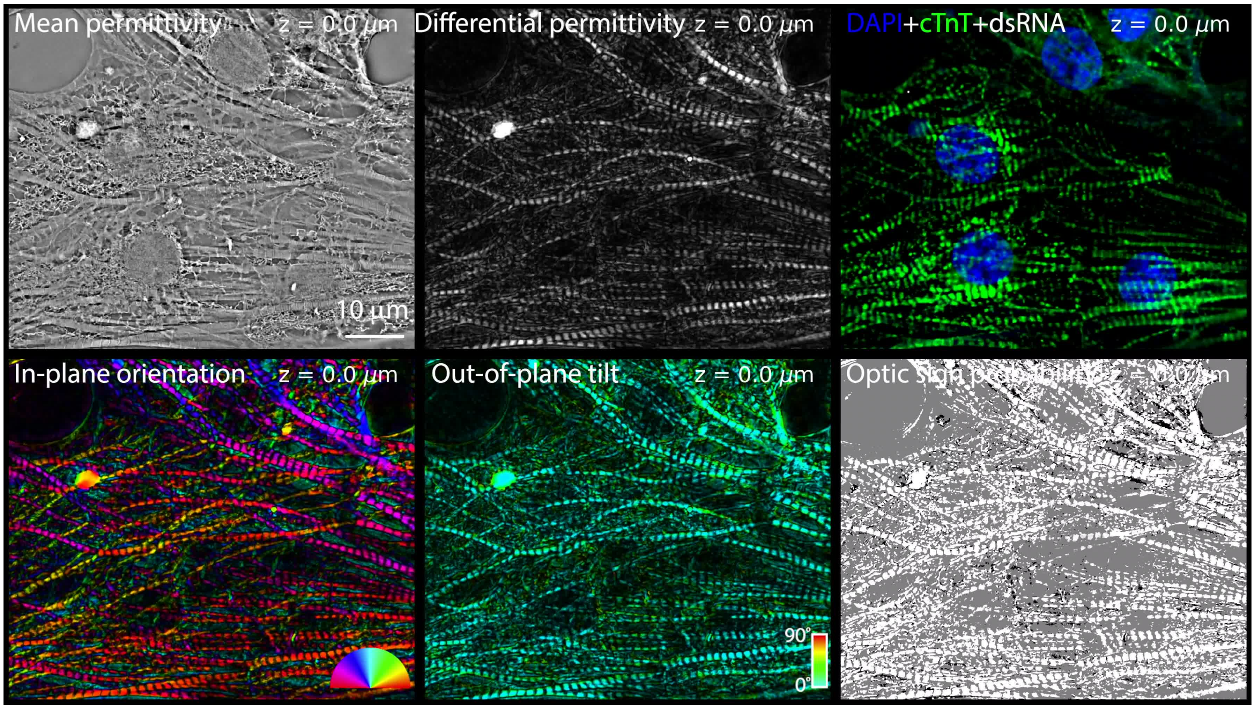

SUPPLEMENTARY VIDEO 6. z-stacks of 3D mean permittivity, 3D differential permittivity, 3D orientation (rendered separately in in-plane orientation and out-of-plane tilt), optic sign probability, and fluorescence images of fixed uninfected cardiomyocytes shown in fig. 5A.

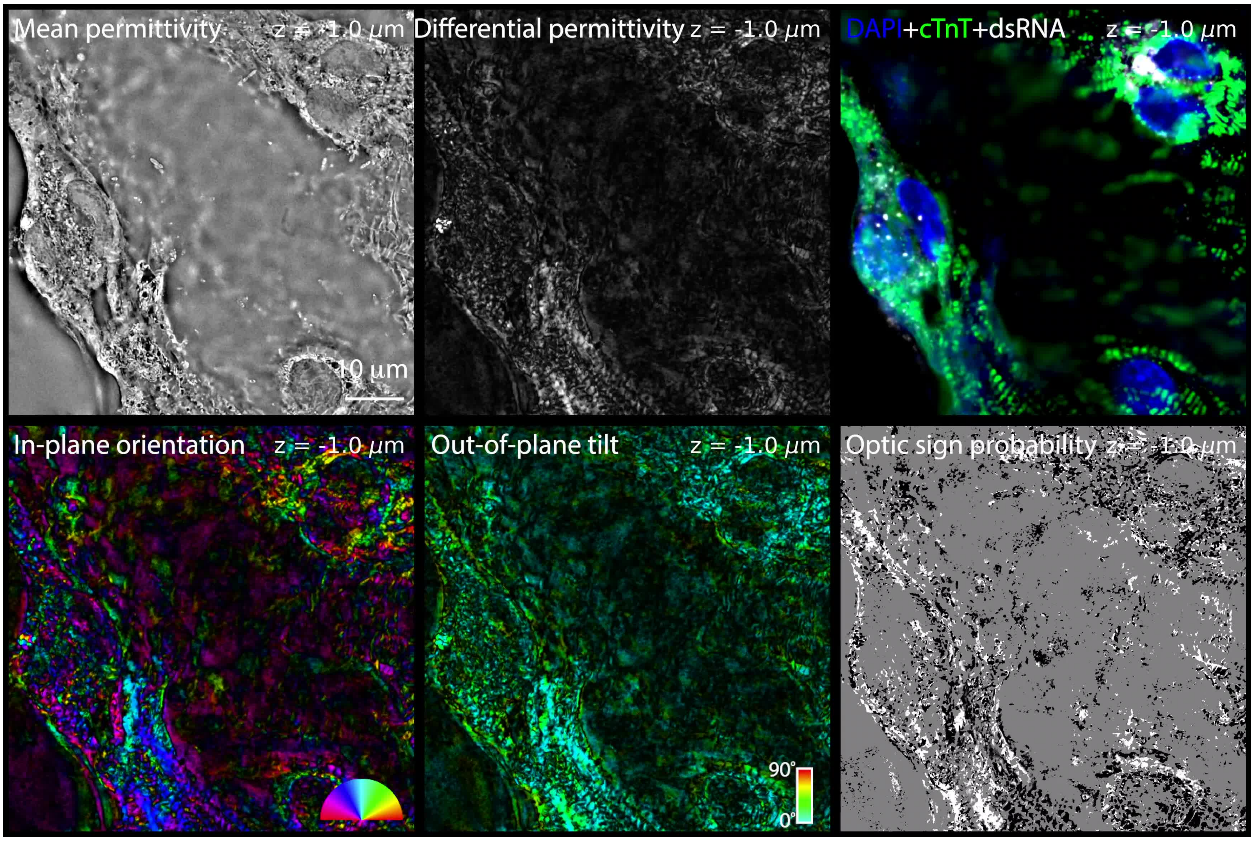

SUPPLEMENTARY VIDEO 7. z-stacks of 3D mean permittivity, 3D differential permittivity, 3D orientation (rendered separately in in-plane orientation and out-of-plane tilt), optic sign probability, and fluorescence images of SARS-COV-2 infected cardiomyocytes shown in fig. 5D (left).

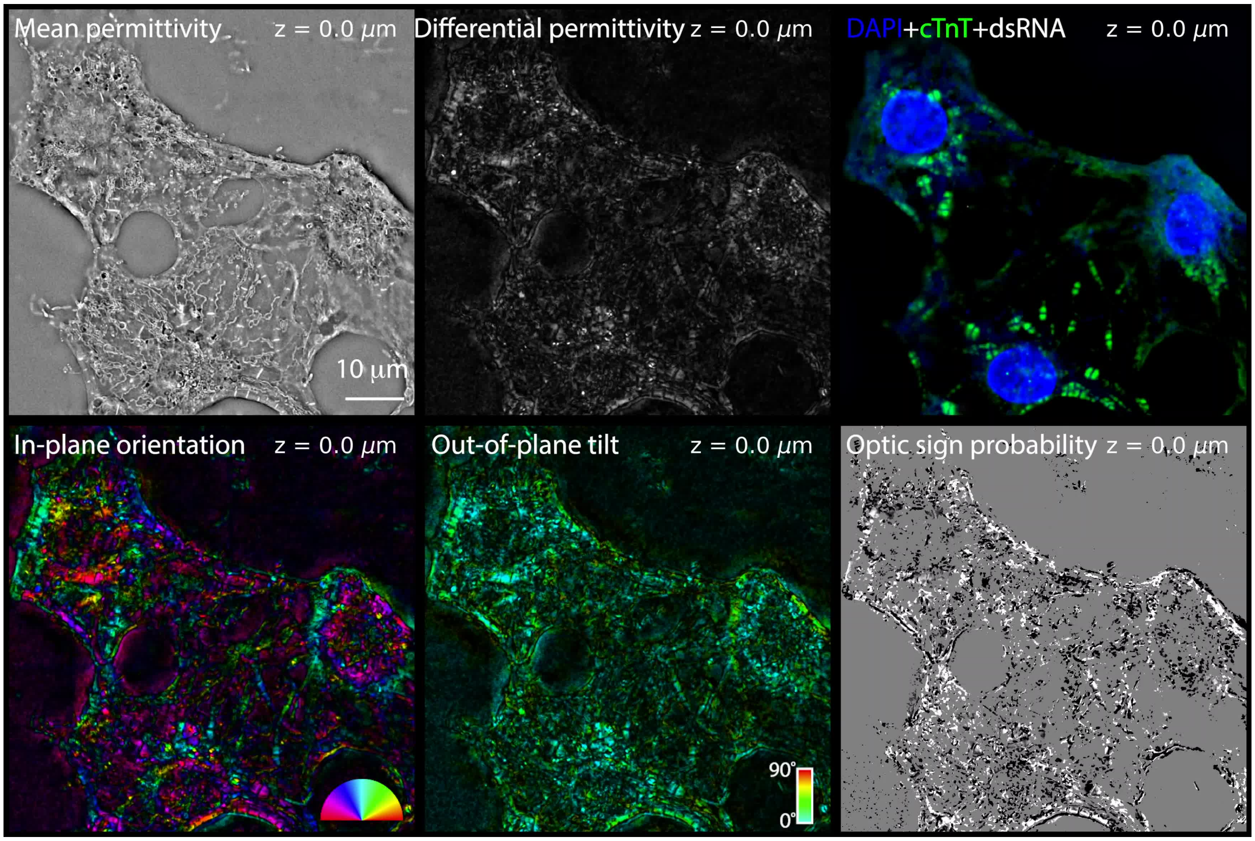

SUPPLEMENTARY VIDEO 8. z-stacks of 3D mean permittivity, 3D differential permittivity, 3D orientation (rendered separately in in-plane orientation and out-of-plane tilt), optic sign probability, and fluorescence images of SARS-COV-2 infected cardiomyocytes shown in fig. 5D (right).

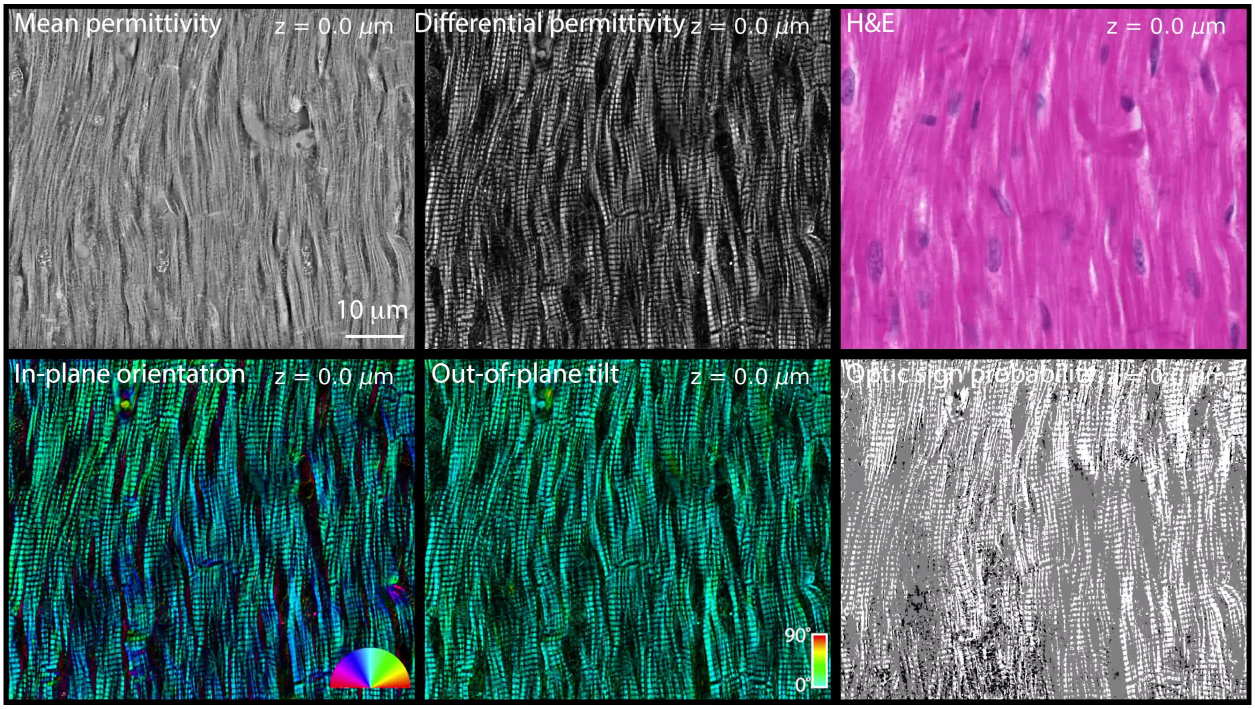

Supplementary Video 9. z-stacks of 3D mean permittivity, 3D differential permittivity, 3D orientation (rendered separately in in-plane orientation and out-of-plane tilt), optic sign probability, and H&E images of the mammal cardiac tissue shown in fig. 6A (top).

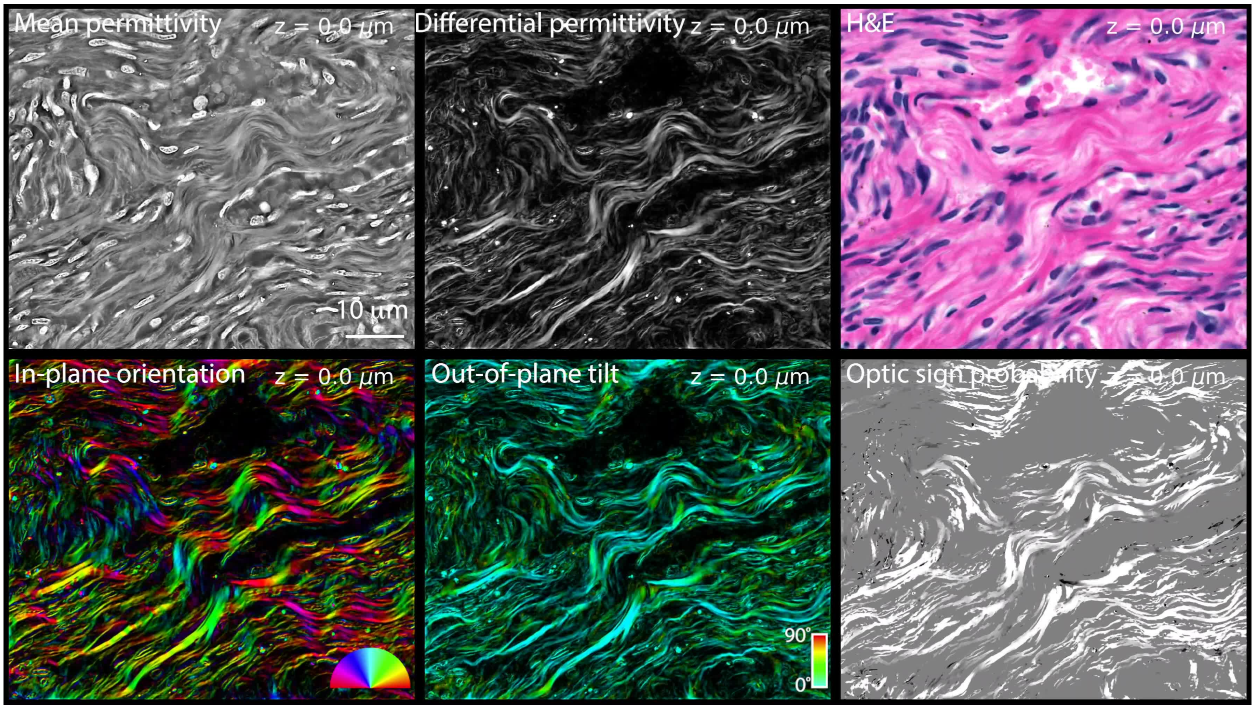

SUPPLEMENTARY VIDEO 10. z-stacks of 3D mean permittivity, 3D differential permittivity, 3D orientation (rendered separately in in-plane orientation and out-of-plane tilt), optic sign probability, and H&E images of the human uterus tissue shown in fig. 6A (bottom).

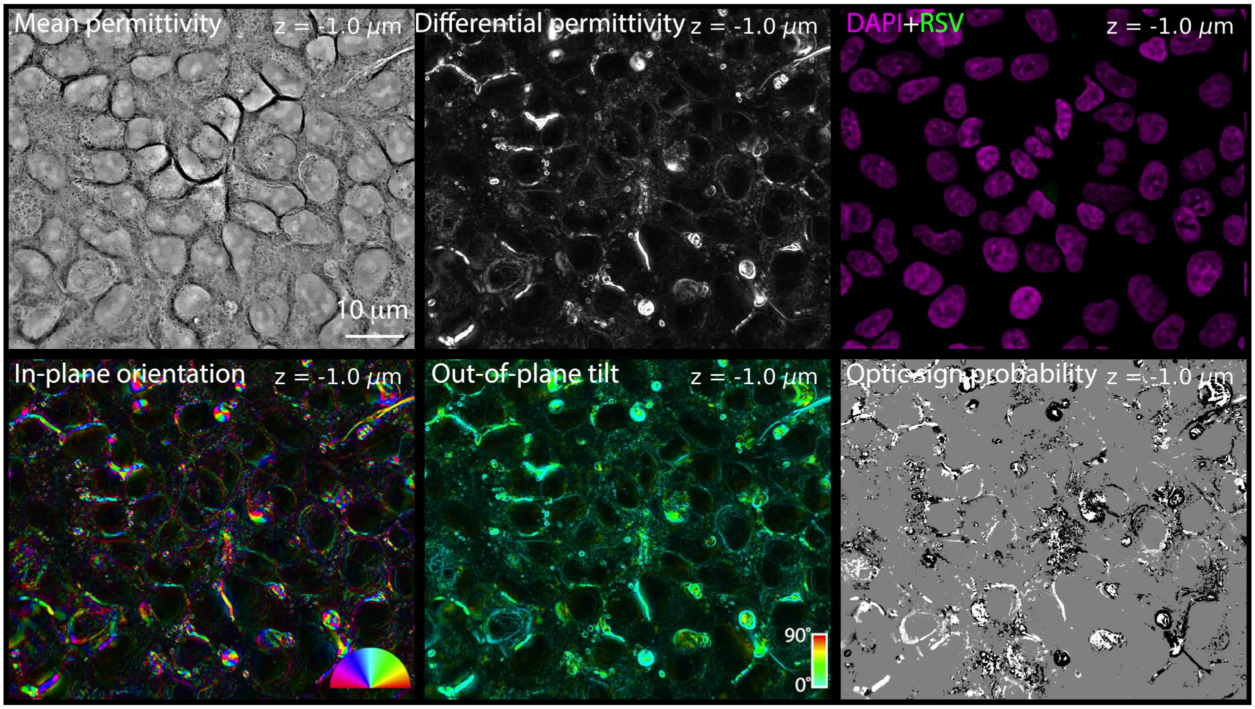

SUPPLEMENTARY VIDEO 11. *z*-stacks of 3D mean permittivity, 3D differential permittivity, 3D orientation (rendered separately in in-plane orientation and out-of-plane tilt), optic sign probability, and fluorescence images of fixed uninfected A549 cells shown in Figure 2

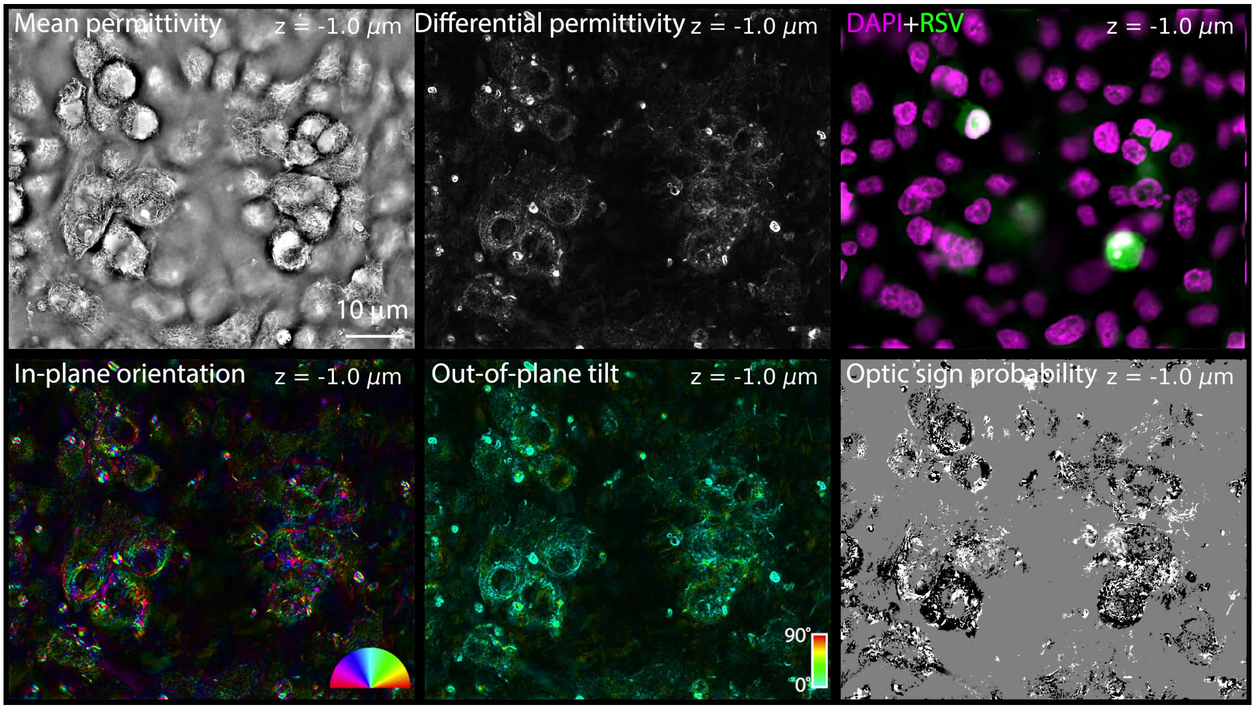

SUPPLEMENTARY VIDEO 12. z-stacks of 3D mean permittivity, 3D differential permittivity, 3D orientation (rendered separately in in-plane orientation and out-of-plane tilt), optic sign probability, and fluorescence images of RSV-infected A549 cells shown in Figure 3.

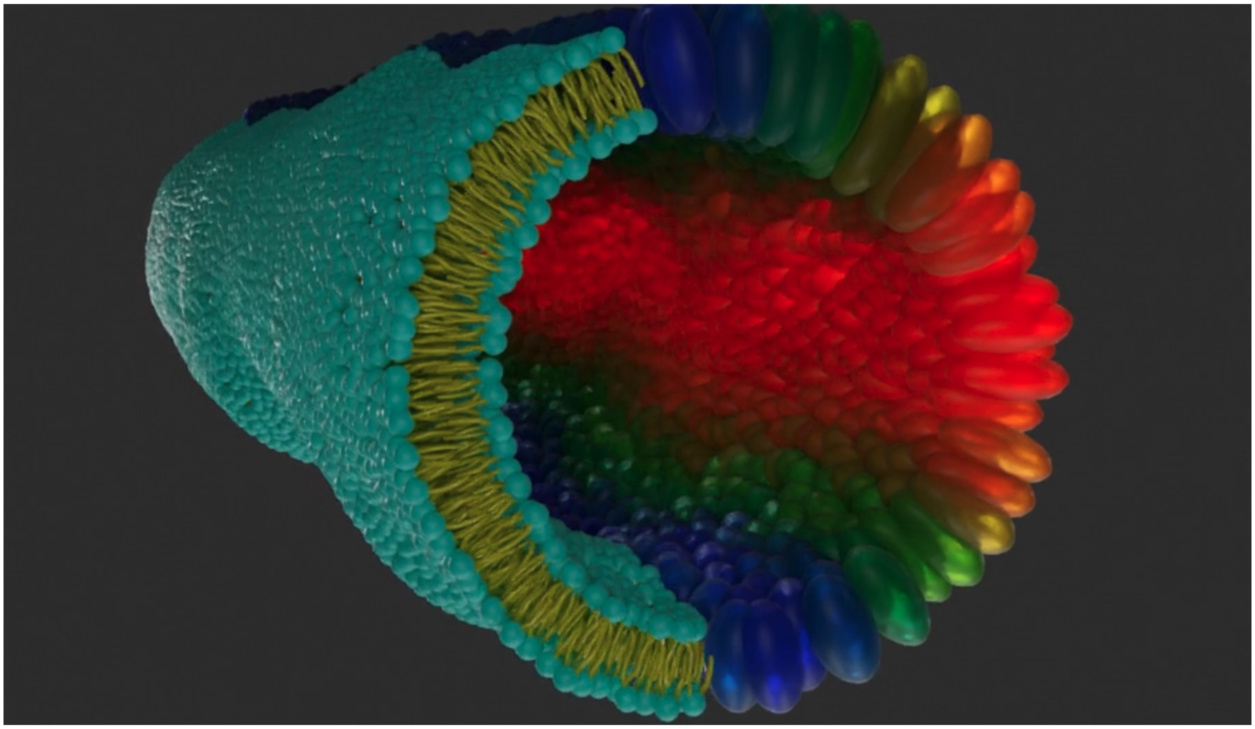

SUPPLEMENTARY VIDEO 13. 3D animation illustrating the key concepts underlying permittivity tensor imaging: (1) The architecture of a cylindrical lipid bilayer is represented as a positively uniaxial permittivity tensor distribution at high-resolution, but as a negatively uniaxial permittivity tensor at coarse resolution. (2) The permittivity tensor of an optical section in the brain tissue are encoded in images. Notice that the structure affects the images, but cannot be interpreted from the images. The field of view shown here is the same as in fig. 1. (3) The image formation model that maps the isotropic material or anisotropic material to images is illustrated. (4) The inverse algorithm recovers the components of the uniaxial PT from the high-dimensional image data. The field of view shown here is the same as in fig. 1. (5) Measurements of the permittivity tensors at the scales ranging from the single axon to the corpus callosum are illustrated.
