# Supplementary material for: Permittivity tensor imaging: modular label-free imaging of 3D dry mass and 3D orientation at high resolution
Source: Nat Methods. 2024 Jun 18;21(7):1257–74. doi: 10.1038/s41592-024-02291-w (PMC11239526; doi:10.1038/s41592-024-02291-w)
Supplement: Supplementary file 2 — Reporting Summary [file 41592_2024_2291_MOESM2_ESM.pdf]

nature research

Corresponding author(s): Shalin B. Mehta

Last updated by author(s): Mar 28, 2024

Reporting Summary

Nature Research wishes to improve the reproducibility of the work that we publish. This form provides structure for consistency and transparency in reporting. For further information on Nature Research policies, see our Editorial Policies and the Editorial Policy Checklist.

Statistics

For all statistical analyses, confirm that the following items are present in the figure legend, table legend, main text, or Methods section.

n/a	Confirmed	
☐	☒	The exact sample size (*n*) for each experimental group/condition, given as a discrete number and unit of measurement
☐	☒	A statement on whether measurements were taken from distinct samples or whether the same sample was measured repeatedly
☒	☐	The statistical test(s) used AND whether they are one- or two-sided<br>*Only common tests should be described solely by name; describe more complex techniques in the Methods section.*
☒	☐	A description of all covariates tested
☒	☐	A description of any assumptions or corrections, such as tests of normality and adjustment for multiple comparisons
☐	☒	A full description of the statistical parameters including central tendency (e.g. means) or other basic estimates (e.g. regression coefficient) AND variation (e.g. standard deviation) or associated estimates of uncertainty (e.g. confidence intervals)
☒	☐	For null hypothesis testing, the test statistic (e.g. *F*, *t*, *r*) with confidence intervals, effect sizes, degrees of freedom and *P* value noted<br>*Give P values as exact values whenever suitable.*
☒	☐	For Bayesian analysis, information on the choice of priors and Markov chain Monte Carlo settings
☒	☐	For hierarchical and complex designs, identification of the appropriate level for tests and full reporting of outcomes
☒	☐	Estimates of effect sizes (e.g. Cohen's *d*, Pearson's *r*), indicating how they were calculated

Our web collection on statistics for biologists contains articles on many of the points above.

Software and code

Policy information about availability of computer code

Data collection	The data acquisition requires the following softwares and packages:<br>1. Micromanager 2.0.0-gamma (https://micro-manager.org/)<br>2. mm2python (https://github.com/bryantChhun/mm2Python)<br>3. PySpin (spinnaker_python-1.25.0.52-cp37-cp37m-win_amd64.whl, https://www.flir.com/products/spinnaker-sdk/)<br>4. Adafruit LCD control (https://learn.adafruit.com/adafruit-1-44-color-tft-with-micro-sd-socket)
Data analysis	1. Algorithms for PTI reconstruction were implemented in python and are available as a GitHub repository (https://github.com/mehta-lab/waveorder).<br>2. FDTD simulation software: Meep (pymeep, 1.21.0, https://meep.readthedocs.io/en/latest/)

For manuscripts utilizing custom algorithms or software that are central to the research but not yet described in published literature, software must be made available to editors and reviewers. We strongly encourage code deposition in a community repository (e.g. GitHub). See the Nature Research guidelines for submitting code & software for further information.

Data

Policy information about availability of data

All manuscripts must include a data availability statement. This statement should provide the following information, where applicable:

- Accession codes, unique identifiers, or web links for publicly available datasets
- A list of figures that have associated raw data
- A description of any restrictions on data availability

Experimental data reported in this manuscript are available at Zenodo (https://doi.org/10.5281/zenodo.5951978).

This includes raw data and processed data for Figure 3A, Figure 3-supplementary 1, Figure 4D, E, F, Figure 4-supplement 1, Figure 5 A, C, D, Figure 6.

Simulated PTI images and reconstructions (Figure 1-supplement 3) are available as examples documented in our open source(https://github.com/mehta-lab/waveorder) repository.

Allen brain reference atlas (https://mouse.brain-map.org/static/atlas) is used to register the anatomical landmarks of the mouse brain section in Figure 4A, B, C.

Field-specific reporting

Please select the one below that is the best fit for your research. If you are not sure, read the appropriate sections before making your selection.

☒ Life sciences ☐ Behavioural & social sciences ☐ Ecological, evolutionary & environmental sciences

For a reference copy of the document with all sections, see nature.com/documents/nr-reporting-summary-flat.pdf

Life sciences study design

All studies must disclose on these points even when the disclosure is negative.

Sample size	Figure 4: We report cell architecture and cytopathic effects on the cell architecture that have been reported with other independent studies. Therefore, we imaged ~4 fields of view to evaluate the phenotypes accessible with PTI. Each field of view provided data from at least 15 cells, whose phenotypes were consistent with phenotypes shown in the figure.<br>Figure 5: Each field of view of uninfected and infected cells provided data from ~60 cells, whose phenotypes were consistent with phenotypes shown in the figure.
Data exclusions	No data were excluded from analysis
Replication	Figure 2: The laser-written target was imaged with PTI at least 3 times.<br>Figure 3: The full mouse brain section is imaged with PTI in 20x objective (29x 21 fields of view stitch together). The same section is then imaged in 3D with 63x objective for 17x9 fields of view.<br>Figure 4: Three technical replicates of Mock and SARS-COV-2-infected cardiomyocytes were analyzed according to reference (55). Two fields of view were acquired in each condition. One field of view of Mock condition and two fields of view of infected condition are shown.<br>Figure 5: Three independent experiments comparing Mock and RSV-infected A549 cells were conducted and examined. From one of the replicates, two fields of view were acquired in mock and infected conditions. One field of view of each condition is shown.<br>Figure 6: Two fields of view of each H&E slides presented were imaged with PTI, and one field of view from each slide is presented.
Randomization	Randomization was not needed as we are evaluating a new imaging technology, rather than predicting a biological mechanism.
Blinding	Blinding was not possible or needed as we are evaluating a new imaging technology, rather than predicting a biological mechanism.

Reporting for specific materials, systems and methods

We require information from authors about some types of materials, experimental systems and methods used in many studies. Here, indicate whether each material, system or method listed is relevant to your study. If you are not sure if a list item applies to your research, read the appropriate section before selecting a response.

Materials & experimental systems

n/a	Involved in the study
☐	☒ Antibodies
☐	☒ Eukaryotic cell lines
☒	☐ Palaeontology and archaeology
☐	☒ Animals and other organisms
☒	☐ Human research participants
☒	☐ Clinical data
☒	☐ Dual use research of concern

Methods

n/a	Involved in the study
☒	☐ ChIP-seq
☒	☐ Flow cytometry
☒	☐ MRI-based neuroimaging

Antibodies

Antibodies used	Figure 4: Troponin T (Abcam, ab45932, dilution 1:400) and Alexa Fluor 488 Donkey anti-mouse IgG (ThermoFisher, A-21202, dilution 1:400) are used as a primary antibody and a secondary antibody, respectively, to stain the sarcomeres of iPSC-cardiomyocytes.<br>Figure 5: No antibodies were used.
Validation	Figure 4: The antibodies have been validated by previous studies conducted by Bruce Conklin Lab.

Eukaryotic cell lines

Policy information about cell lines

Cell line source(s)	1. iPSC-cardiomyocytes (differentiated from WTc cell line)<br>2. A549 cells (https://www.atcc.org/products/ccl-185)
Authentication	The authenticity of cell lines was established by comparing their growth pattern and morphology with widely reported data.
Mycoplasma contamination	All cell lines tested negative for mycoplasma contamination
Commonly misidentified lines<br>(See ICLAC register)	There is no commonly misidentified cell lines used in the study.

Animals and other organisms

Policy information about studies involving animals; ARRIVE guidelines recommended for reporting animal research

Laboratory animals	Mouse brain tissue section (M. musculus): mouse line maintained in M. Han lab
Wild animals	N/A
Field-collected samples	N/A
Ethics oversight	*Identify the organization(s) that approved or provided guidance on the study protocol, OR state that no ethical approval or guidance was required and explain why not.*

Note that full information on the approval of the study protocol must also be provided in the manuscript.
